# Family support and online English learning effectiveness among Chinese university students: indirect associations through self-regulation and positive emotions

**DOI:** 10.3389/fpsyg.2026.1866298

**Published:** 2026-07-29

**Authors:** Muyang Gao

**Affiliations:** School of Foreign Languages, Beijing University of Technology, Beijing, China

**Keywords:** family support, online English learning, online English learning effectiveness, positive emotions, self-regulation

## Abstract

**Background:**

Online English learning requires university students to manage learning activities across flexible and relatively less supervised digital environments. Although family support may represent a relevant distal socio-contextual resource, its associations with students’ self-regulation, positive emotions, and evaluations of online English learning remain insufficiently understood. This study examined how family support was associated with Chinese university students’ self-reported online English learning effectiveness, both directly and indirectly through self-regulation and positive emotions.

**Methods:**

Drawing on social cognitive theory, self-regulated learning theory, and control-value theory of achievement emotions, the study used a cross-sectional online survey design. The analytic sample comprised 430 Chinese university students with prior university-level online English learning experience. The measurement and structural models were evaluated using structural equation modeling, and specific indirect associations were examined using 5,000 bootstrap resamples and bias-corrected 95% confidence intervals.

**Results:**

Family support was positively associated with self-regulation, positive emotions, and self-reported online English learning effectiveness. Self-regulation was positively associated with both positive emotions and online English learning effectiveness, and positive emotions were positively associated with online English learning effectiveness. The indirect association between family support and online English learning effectiveness through self-regulation was statistically significant [estimate = 0.169, 95% BC CI (0.064, 0.315)]. The focused sequential indirect association through self-regulation and positive emotions was also statistically significant [estimate = 0.110, 95% BC CI (0.050, 0.200)], supporting the focused hypothesis. By contrast, the indirect association through positive emotions alone was not statistically significant [estimate = 0.046, 95% BC CI (-0.001, 0.141)]. The direct association between family support and online English learning effectiveness remained statistically significant.

**Conclusion:**

The findings identify self-regulation as a proximal regulatory process within the theory-specified model and indicate that positive emotions are relevant when considered alongside students’ self-regulatory management of online learning. Family support was associated with students’ evaluations of online English learning both directly and through specific regulatory and affective pathways. Because the study used cross-sectional self-report data and a non-probability sample, the findings should be interpreted as theory-consistent associations rather than evidence of temporal ordering or causal mediation.

## 1 Introduction

Digital technologies, online platforms, and artificial-intelligence-enabled tools continue to reshape the conditions under which English is learned. Recent scholarship has increasingly focused on how online English learning environments can be pedagogically organized and optimized rather than merely on whether digital technologies are available (Liu and Yao, 2026). A recent meta-analysis integrating 48 empirical studies further reported an overall moderate positive effect of artificial-intelligence-supported foreign-language learning, although the magnitude of the effects varied across language skills, psychological-affective outcomes, educational levels, and technology types (Wang et al., 2026).

In higher education, online English learning frequently takes place across flexible and distributed environments, including formal course platforms, mobile applications, artificial-intelligence-supported tools, and informal learning settings. Such flexibility expands access to learning resources but also transfers greater responsibility to learners for setting goals, organizing learning activities, monitoring progress, regulating effort, and seeking assistance when difficulties arise. Recent evidence from 413 Chinese university foreign-language learners showed that goal setting, task strategies, and help-seeking strategies were positively associated with online learning engagement, while family economic background contributed to differences in the relationship between environmental management and engagement (Hui and Wang, 2025). These findings indicate that online learning experiences are associated with both learners’ self-regulatory processes and the contextual conditions in which learning occurs.

Recent empirical research has also shown that technology-mediated foreign-language learning involves cognitive-regulatory and affective processes. Artificial-intelligence-mediated interaction and digital language-learning applications have been examined in relation to speaking development, willingness to communicate, and online learning engagement (Fathi et al., 2024; Ouyang et al., 2024; Zhou et al., 2025). Automated and generative feedback tools have also been investigated in relation to writing-feedback literacy, writing engagement, writing performance, and learners’ ideal L2 writing self (Rad et al., 2024; Shi et al., 2025). Current research has additionally focused on learners’ metacognitive awareness, task motivation, and experiences of using artificial-intelligence-supported feedback (Teng, 2025; Zare et al., 2025). Taken together, this evidence suggests that the effectiveness of contemporary online English learning depends not only on the availability of digital tools but also on how learners regulate their activities and experience the learning process.

Family support represents a further socio-contextual resource that may remain relevant when English learning extends beyond formal classroom settings. A recent mixed-methods study showed that parents participated in children’s home-based online English learning by organizing routines, selecting digital tools, and supporting language activities, while also experiencing constraints related to digital literacy, time, and teaching responsibilities (Qiu et al., 2026). Although that study focused on young children rather than university students, it provides current evidence that family resources and practices can shape the conditions under which online language learning occurs. At the university level, however, students are expected to assume substantially greater responsibility for their learning. Family support may therefore operate less through direct instruction and more through emotional encouragement, autonomy-supportive communication, and access to learning-related resources.

The present study consequently examines family support as a distal socio-contextual resource associated with university students’ self-regulation, positive emotions, and self-reported online English learning effectiveness. Teacher and peer support are more directly embedded in particular courses, instructional arrangements, and opportunities for interaction, whereas family support may extend across courses and learning settings. The study does not assume that family support is more important than other forms of support. Rather, it addresses the comparatively underexamined role of perceived family support in students’ regulatory and affective experiences during contemporary online English learning. Because teacher and peer support were not measured, the study does not test comparative or interactive associations among different sources of social support.

Against this background, the study addresses the following research question:

*RQ*: How is family support associated with Chinese university students’ self-reported online English learning effectiveness, both directly and indirectly through self-regulation and positive emotions?

Accordingly, the objective of the study is to evaluate the direct association between family support and online English learning effectiveness, the specific indirect associations involving self-regulation and positive emotions, and, as the focal theoretical test, the sequential indirect association through self-regulation and positive emotions.

The study contributes to educational psychology in three respects. First, it integrates family support, self-regulation, positive emotions, and online English learning effectiveness within an environment–cognition–affect–outcome framework. Second, it conceptualizes family support as a distal socio-contextual resource associated with university students’ autonomous management of online English learning rather than as a form of direct parental instruction or control. Third, it evaluates whether the observed associations are consistent with a focused sequential pathway linking family support, self-regulation, positive emotions, and online English learning effectiveness.

## 2 Literature review and theoretical framework

### 2.1 Online English learning in the higher-education context

Online English learning now encompasses a diverse range of digital environments, including learning-management systems, mobile applications, synchronous and asynchronous courses, automated feedback tools, and artificial-intelligence-supported learning platforms. Recent scholarship has emphasized that the central issue is no longer merely whether digital technologies are available, but how such technologies are pedagogically organized and how learners engage with them (Liu and Yao, 2026).

Current empirical research indicates that digital technologies can support multiple dimensions of foreign-language learning. Artificial-intelligence-mediated interaction has been examined in relation to speaking development and willingness to communicate (Fathi et al., 2024), while AI-integrated language-learning applications have been associated with willingness to communicate and engagement in online classes (Ouyang et al., 2024). AI-chatbot-integrated mobile-assisted learning has also been investigated as a means of supporting English-speaking practice in higher education (Zhou et al., 2025). Automated and generative feedback tools have been examined in relation to writing-feedback literacy, engagement, writing performance, and learners’ ideal L2 writing self (Rad et al., 2024; Shi et al., 2025). These findings demonstrate the expanding pedagogical possibilities of online English learning, but they also indicate that technology-supported outcomes vary across tools, tasks, learner groups, and outcome domains.

Self-regulation is particularly important in this context because learners must organize their activities across flexible and relatively less supervised environments. Recent evidence from Chinese university foreign-language learners showed that goal setting, task strategies, and help-seeking strategies were positively associated with online learning engagement, while configurations combining self-regulatory strategies and contextual conditions were associated with higher engagement (Hui and Wang, 2025). This evidence supports the view that learners’ management of goals, strategies, resources, and assistance is closely related to their participation in online language learning.

Recent research has also examined learners’ metacognitive awareness, task motivation, and experiences of using artificial-intelligence-supported feedback in foreign-language learning (Teng, 2025; Zare et al., 2025). These studies suggest that the educational value of digital tools depends partly on how actively learners monitor, evaluate, and regulate their use of technological support.

Affective experiences are also integral to digital foreign-language learning. A 2026 meta-analysis integrating 48 empirical studies reported that artificial-intelligence-supported foreign-language learning had positive effects on several psychological-affective outcomes, including learning engagement, motivation, enjoyment, willingness to communicate, collaboration, and self-efficacy, although the magnitude of the effects differed substantially across outcomes (Wang et al., 2026). In particular, the reported effect on learning enjoyment was smaller than those on engagement, motivation, and willingness to communicate. This variation indicates that positive emotions are relevant to online English learning but should not be treated as a uniform or isolated route to learning effectiveness.

Taken together, recent evidence positions online English learning as an interaction among digital conditions, self-regulatory processes, and affective experiences. However, current research has concentrated primarily on technological and instructional support. The role of family support as a broader socio-contextual resource in university students’ online English learning has received comparatively less direct empirical attention.

### 2.2 Integrated theoretical framework

This study integrates social cognitive theory, self-regulated learning theory, and control-value theory of achievement emotions to explain how family support may be associated with university students’ online English learning effectiveness. These theories are complementary because they address different components of the proposed process: socio-contextual resources, learners’ regulatory processes, achievement-related emotions, and self-reported online English learning effectiveness.

First, social cognitive theory emphasizes reciprocal relationships among environmental influences, personal processes, and behavior (Bandura, 1989). From this perspective, family support can be understood as a socio-contextual resource associated with students’ learning beliefs, perceived security, motivation, and behavioral engagement. In online English learning, emotional encouragement, learning-related resources, and autonomy-supportive communication may provide conditions compatible with students’ independent management of learning activities.

Second, self-regulated learning theory explains how learners actively manage their learning through goal setting, strategic planning, self-monitoring, effort regulation, and reflection (Zimmerman, 2008). These processes are particularly relevant in online English learning because students often experience greater flexibility but less immediate external supervision. Self-regulation is therefore conceptualized in this study as a proximal cognitive-regulatory process associated with students’ adaptive management of learning under supportive contextual conditions.

Third, control-value theory of achievement emotions explains how students’ emotions are related to their appraisals of control and value in academic activities (Pekrun, 2006). Students who perceive greater control over learning requirements may be more likely to experience positive emotions such as enjoyment, interest, and pride. In the present framework, self-regulation is expected to be associated with positive emotions because effective goal management, progress monitoring, and strategy adjustment may strengthen students’ perceived control over online English learning.

Taken together, the proposed framework positions family support as an environmental resource, self-regulation as a cognitive-regulatory process, positive emotions as an affective process, and online English learning effectiveness as a self-reported learning outcome. The integration of these components provides the conceptual basis for examining how family support is related to students’ regulatory and affective experiences in online English learning.

### 2.3 Theory-specified sequential associations between self-regulation and positive emotions

The proposed ordering from self-regulation to positive emotions is grounded in self-regulated learning theory and control-value theory (Pekrun, 2006; Zimmerman, 2008). Students who can set goals, manage time, monitor learning progress, and adjust strategies may experience a stronger sense of control when engaging in online English learning. Such perceived control may, in turn, be associated with greater enjoyment, interest, and pride.

Positive emotions may also support persistence and strategic engagement in actual learning processes. Recent evidence indicates that affective outcomes such as enjoyment, motivation, willingness to communicate, and engagement are meaningfully implicated in technology-supported foreign-language learning, although their relationships vary across learning conditions and outcomes (Wang et al., 2026). The relationship between self-regulation and positive emotions may therefore be reciprocal. Nevertheless, the present framework gives theoretical priority to the ordering from self-regulation to positive emotions because it focuses on strategic learning management and perceived control as conditions associated with positive achievement emotions.

### 2.4 Research objective and focused hypothesis

The literature reviewed above positions family support as a distal socio-contextual resource, self-regulation as a proximal cognitive-regulatory process, positive emotions as an affective process, and online English learning effectiveness as a self-reported learning outcome. Within this framework, family support may be associated with students’ learning evaluations both directly and through the regulatory and affective processes involved in managing online English learning.

Recent evidence indicates that family-related resources can shape the practical conditions under which online English learning occurs, although current evidence concerning family involvement has focused primarily on younger learners (Qiu et al., 2026). Research involving Chinese university foreign-language learners further shows that goal setting, task strategies, and help-seeking strategies are positively associated with online learning engagement (Hui and Wang, 2025). Recent studies and meta-analytic evidence also indicate that regulatory, motivational, and affective experiences are closely implicated in technology-supported foreign-language learning, although their associations vary across learning contexts and outcome domains (Teng, 2025; Wang et al., 2026; Zare et al., 2025).

In accordance with the research question stated in the Introduction, the objective of the present study is to examine how family support is associated with self-reported online English learning effectiveness, with particular attention to the regulatory and affective processes represented by self-regulation and positive emotions.

Accordingly, the following focused hypothesis is proposed:

*H1*: Family support will show a positive sequential indirect association with self-reported online English learning effectiveness through self-regulation and positive emotions, such that family support is positively associated with self-regulation, self-regulation is positively associated with positive emotions, and positive emotions are positively associated with online English learning effectiveness.

The remaining direct and specific indirect associations are addressed as components of the research question rather than as separate hypotheses.

## 3 Research design and methodology

### 3.1 Study design, sampling, eligibility criteria, and participants

This study used a cross-sectional online survey design. The target population comprised university students in China with prior experience of university-level online English learning. The survey was distributed to students in Beijing, Shanghai, Hunan, Hubei, Jiangsu, and Guangdong. Recruitment across these regions was intended to increase geographical and educational heterogeneity within the sample rather than to produce a nationally representative sample (See Supplementary Material 1).

A non-probability sampling strategy combining convenience sampling and snowball referrals was used because the study focused on geographically dispersed students with relevant online English learning experience. Participants were recruited through online English-learning platforms, university course-management channels, and peer referrals. Initial respondents were invited to share the survey link with potentially eligible classmates or acquaintances.

The inclusion criteria were: (a) being enrolled as a university student in China; (b) being in the second year of university or above; (c) having prior experience of university-level online English learning; and (d) providing electronic informed consent. First-year students were not included because the study focused on students who had accumulated university-level online English learning experience. Students without relevant online English learning experience were outside the target population.

Participation was voluntary. Before completing the questionnaire, participants were informed about the purpose of the study, the procedures used to protect anonymity and confidentiality, and their right to discontinue participation. Electronic informed consent was obtained before the survey began.

A total of 458 questionnaires were collected. Twenty-eight questionnaires were excluded before analysis, leaving a final analytic sample of 430 participants.

Although participants were recruited from several regions of China, the sample should not be regarded as statistically representative of all Chinese university students or all Chinese university students engaged in online English learning. Multi-region recruitment broadened the range of participants included in the study but did not eliminate the potential for self-selection and coverage bias associated with convenience and snowball sampling. Participant flow and the academic and online English learning characteristics of the analytic sample are presented in Table 1.

**TABLE 1:** Participant flow and characteristics of the analytic sample.

Domain	Characteristic	Category	*n*	%
Participant flow	Questionnaires	Initially collected	458	–
		Excluded before analysis	28	–
		Included in the analytic sample	430	–
Academic characteristics	Field of study	Liberal arts	104	24.19
		Science and engineering	17	3.95
		Business-related majors	16	3.72
		Other majors	293	68.14
	Year of study	Second year	176	40.93
		Third year	183	42.56
		Fourth year	71	16.51
Online English learning profile	Self-reported English proficiency	Beginner	251	58.37
		Intermediate	122	28.37
		Upper-intermediate	44	10.23
		Advanced	3	0.70
		Proficient	10	2.33
	Weekly online English study time	Less than 5 h	320	74.42
		5–10 h	76	17.67
		11–15 h	18	4.19
		16 h or more	16	3.72
	Online English learning modes*^a^*	Self-directed learning	384	89.30
		Participation in online courses	132	30.70
		Attendance at online tutorial classes	59	13.72
		Other	7	1.63
	Primary purpose of online English learning	Study abroad	45	10.47
		Career development	123	28.60
		Hobbies and interests	97	22.56
		Academic research	34	7.91
		Other	131	30.47

Percentages for participant characteristics were calculated using the final analytic sample (*N* = 430). Percentages are not reported for participant-flow counts.

*^a^*Multiple responses were permitted for online English learning modes; therefore, percentages within this characteristic do not sum to 100%.

### 3.2 Questionnaire development and measures

The questionnaire was developed in Chinese for the context of university students’ online English learning in China. It contained literature-informed and context-adapted items assessing family support, self-regulation, positive emotions, and online English learning effectiveness. The questionnaire was not intended to reproduce any single standardized instrument in its entirety. Established theoretical and measurement literature guided construct definition and item development, after which the wording was contextualized for online English learning (Boateng et al., 2018).

Before the main survey, the questionnaire was reviewed by two scholars with expertise in English education and educational psychology. Their feedback was used to improve item clarity, relevance, and contextual appropriateness. A pilot test was subsequently conducted with 30 university students who had experience of online English learning. Minor wording refinements were made on the basis of item comprehensibility and response clarity. Pilot responses were not included in the final sample.

All substantive items were rated on a five-point Likert scale ranging from 1 (strongly disagree) to 5 (strongly agree). The initial questionnaire contained 35 substantive items across the four focal constructs. Table 2 presents the initial item pools and their internal-consistency coefficients, while Tables 3, 4 present the reliability and validity evidence for the indicators retained in the final measurement model.

**TABLE 2:** Initial item pools, retained CFA/SEM indicators, and internal consistency.

Construct/scale	Initial item pool (n)	Retained item codes	Retained indicators (n)	Cronbach’s α	Standardized α
Family support	9	Q28, Q29, Q30, Q34	4	0.944	0.944
Self-regulation	12	Q10, Q11, Q13, Q14, Q21	5	0.969	0.967
Positive emotions	3	Q22, Q23, Q24	3	0.772	0.773
Online English learning effectiveness	11	Q49, Q53, Q54, Q58	4	0.967	0.967

The initial questionnaire included 35 substantive items. The overall Kaiser–Meyer–Olkin (KMO) value for the initial questionnaire was 0.970. Cronbach’s alpha coefficients were calculated using the initial item pool for each construct. The retained item codes are listed together with their corresponding constructs or scales. These retained indicators were used in the final CFA/SEM measurement model and are reported in detail in Table 3.

**TABLE 3:** CFA measurement properties of retained indicators in the final measurement model.

Corresponding construct/scale	Item code	Unstandardized estimate	S.E.	C.R.	*p*	Standardized loading	SMC	CR	AVE
Family support	Q28	1.000	–	–	–	0.858	0.736	0.922	0.748
Family support	Q29	1.021	0.040	25.355	< 0.001	0.909	0.826	–	–
Family support	Q30	0.964	0.039	24.802	< 0.001	0.896	0.803	–	–
Family support	Q34	0.865	0.043	20.093	< 0.001	0.791	0.626	–	–
Self-regulation	Q10	1.000	–	–	–	0.793	0.629	0.919	0.696
Self-regulation	Q11	1.069	0.053	20.075	< 0.001	0.857	0.734	–	–
Self-regulation	Q13	1.134	0.054	20.948	< 0.001	0.885	0.783	–	–
Self-regulation	Q14	1.106	0.053	20.882	< 0.001	0.882	0.778	–	–
Self-regulation	Q21	0.904	0.054	16.744	< 0.001	0.745	0.555	–	–
Positive emotions	Q22	1.000	–	–	–	0.901	0.812	0.921	0.797
Positive emotions	Q23	1.070	0.036	29.558	< 0.001	0.956	0.914	–	–
Positive emotions	Q24	0.918	0.040	23.042	< 0.001	0.815	0.664	–	–
Online English learning effectiveness	Q49	1.000	–	–	–	0.790	0.624	0.915	0.730
Online English learning effectiveness	Q53	1.043	0.049	21.310	< 0.001	0.908	0.824	–	–
Online English learning effectiveness	Q54	1.006	0.049	20.620	< 0.001	0.882	0.778	–	–
Online English learning effectiveness	Q58	0.970	0.051	19.146	< 0.001	0.833	0.694	–	–

S.E., standard error; C.R., critical ratio; SMC, squared multiple correlation; CR, composite reliability; AVE, average variance extracted. CR and AVE are construct-level coefficients and are reported once for each construct. Only indicators retained in the final measurement model are included.

**TABLE 4:** Discriminant validity of the measurement model.

Construct	AVE	Self-regulation	Positive emotions	Family support	Online English learning effectiveness
Self-regulation	0.696	0.834	0.893	0.865	0.854
Positive emotions	0.797	0.739			
Family support	0.748	0.573	0.423		
Online English learning effectiveness	0.730	0.670	0.643	0.576	

Diagonal values are the square roots of average variance extracted. Values below the diagonal are correlations between the latent constructs.

Family support. Family support referred to students’ perceived emotional encouragement, autonomy-supportive communication, and learning-related resources provided by family members. The initial nine-item pool was developed with reference to the family-support content of the Multidimensional Scale of Perceived Social Support and contextualized for online English learning (Zimet et al., 1988). Representative content concerned emotional encouragement, understanding, and access to learning-related resources. Four indicators were retained. The initial item pool had a Cronbach’s alpha of 0.944. For the retained indicators, standardized factor loadings ranged from 0.791 to 0.909, composite reliability was 0.922, and average variance extracted was 0.748.

Self-regulation. Self-regulation referred to students’ active management of online English learning through goal setting, planning, progress monitoring, strategy adjustment, persistence, and effort regulation. The initial 12-item pool was informed by Zimmerman’s conceptualization of self-regulated learning and adapted to online English learning activities (Zimmerman, 2008). Representative content concerned goal setting, progress monitoring, strategy adjustment, and persistence when difficulties arose. Five indicators were retained. The initial item pool had a Cronbach’s alpha of 0.969. For the retained indicators, standardized factor loadings ranged from 0.745 to 0.885, composite reliability was 0.919, and average variance extracted was 0.696.

Positive emotions. Positive emotions referred to constructive affective experiences during online English learning, including enjoyment, interest, and pride. The construct was assessed using three learning-context items informed by control-value theory and achievement-emotion research (Pekrun, 2006). All three indicators were retained. The scale had a Cronbach’s alpha of 0.772. Standardized factor loadings ranged from 0.815 to 0.956, composite reliability was 0.921, and average variance extracted was 0.797.

Online English learning effectiveness. Online English learning effectiveness referred to students’ subjective appraisal of the effectiveness of their online English learning. The initial 11-item context-specific measure assessed perceived knowledge acquisition, learning progress, attainment or application of learning goals, and overall satisfaction. The construct should therefore be interpreted as self-reported learning effectiveness rather than objectively verified English proficiency, course grades, or platform-recorded learning behavior. Four indicators were retained. The initial item pool had a Cronbach’s alpha of 0.967. For the retained indicators, standardized factor loadings ranged from 0.790 to 0.908, composite reliability was 0.915, and average variance extracted was 0.730.

The square roots of the average variance extracted values for family support (0.865), self-regulation (0.834), positive emotions (0.893), and online English learning effectiveness (0.854) exceeded their respective correlations with the other constructs. The reliability and validity coefficients therefore provided evidence consistent with the adequacy of the four measures in the present sample. Detailed indicator-level coefficients and inter-construct correlations are reported in Tables 3, 4.

### 3.3 Data analysis

All analyses were based on the final analytic sample of 430 participants. The analysis proceeded from data screening and descriptive analysis to measurement-model evaluation, structural-model estimation, and the assessment of specific indirect associations.

Of the 458 questionnaires collected, 28 were excluded before analysis because information on one or more core study variables was incomplete. All analyses were conducted using the remaining 430 cases; the excluded questionnaires did not contribute to the descriptive, measurement, structural, or bootstrap results. Within the retained sample, item-level missingness ranged from 0.23 to 1.16%. Full information maximum likelihood estimation was used in the confirmatory factor and structural equation modeling analyses conducted in AMOS 26.0 (Enders and Bandalos, 2001). Under the missing-at-random assumption, this approach used the available item-level information from all 430 retained cases without additional listwise deletion or single-value imputation.

Participant characteristics and online English learning profiles were summarized descriptively. Cronbach’s alpha and standardized alpha were used to examine the internal consistency of the initial item pools. The overall Kaiser–Meyer–Olkin value was examined to assess the suitability of the initial item set for factor analysis.

The measurement model was evaluated before the structural model. Confirmatory factor analysis represented family support, self-regulation, positive emotions, and online English learning effectiveness as latent constructs measured by their retained indicators. One reference loading for each latent construct was fixed at 1.000 for model identification. Indicator performance was examined using standardized factor loadings and squared multiple correlations. Composite reliability and average variance extracted were calculated for each construct. Convergent validity was evaluated using the standardized factor loadings, composite reliability, and average variance extracted. Discriminant validity was evaluated using the Fornell–Larcker criterion, under which the square root of the average variance extracted for each construct was compared with its correlations with the other constructs (Fornell and Larcker, 1981).

Structural equation modeling was then used to address the research question by estimating the direct associations among the four latent constructs within a single theory-specified model. SEM permitted the measurement model and the direct and indirect associations to be estimated simultaneously while accounting for measurement error.

Overall model fit was evaluated collectively using the indices retained in the available final-model analysis records: CMIN/DF, RMSEA, GFI, AGFI, IFI, and TLI. CFI, SRMR, chi-square, degrees of freedom, and the associated *p*-value were not available for verification. Because they could not be validly reconstructed without re-estimating the model from the original covariance data, they were not retrospectively inferred or reported.

Unstandardized and standardized structural coefficients, standard errors, critical ratios, and *p*-values were used to report the direct associations. Approximate explained variance was reported for self-regulation, positive emotions, and online English learning effectiveness. The corresponding directly extracted AMOS output had not been retained; therefore, the *R*^2^ values were reconstructed from the rounded standardized structural solution and the model-implied standardized associations. They are identified as approximate values rather than directly extracted software output.

Specific indirect associations were evaluated using 5,000 bootstrap resamples and bias-corrected 95% confidence intervals (Preacher and Hayes, 2008). An indirect association was considered statistically significant when its confidence interval did not include zero. The analyses examined the indirect association between family support and online English learning effectiveness through self-regulation, the indirect association through positive emotions alone, the indirect association between self-regulation and online English learning effectiveness through positive emotions, and the sequential indirect association from family support through self-regulation and positive emotions to online English learning effectiveness. The sequential indirect association constituted the formal test of H1. The remaining direct and indirect associations addressed the broader research question.

Because all substantive variables were measured using self-report questionnaires completed by the same participants at a single time point, common method bias was considered. Procedural safeguards included voluntary and anonymous participation, confidentiality assurances, instructions encouraging responses based on participants’ own experiences, and the absence of connections between participation and course grades or teacher evaluations. The study did not include a marker variable, temporally separated measurements, multi-informant data, Harman’s single-factor test, or a *post hoc* common-latent-factor analysis; consequently, no statistical correction for common method bias was applied (Podsakoff et al., 2003).

The directional paths and indirect associations were interpreted as theory-specified statistical associations. Given the cross-sectional design, they do not establish temporal ordering or causal mediation (Maxwell and Cole, 2007).

## 4 Results

### 4.1 Measurement results

The initial item pools demonstrated acceptable to excellent internal consistency. Cronbach’s alpha coefficients ranged from 0.772 to 0.969, and standardized alpha coefficients ranged from 0.773 to 0.967 (Table 2). The overall Kaiser–Meyer–Olkin value for the initial questionnaire was 0.970.

**FIGURE 1:**
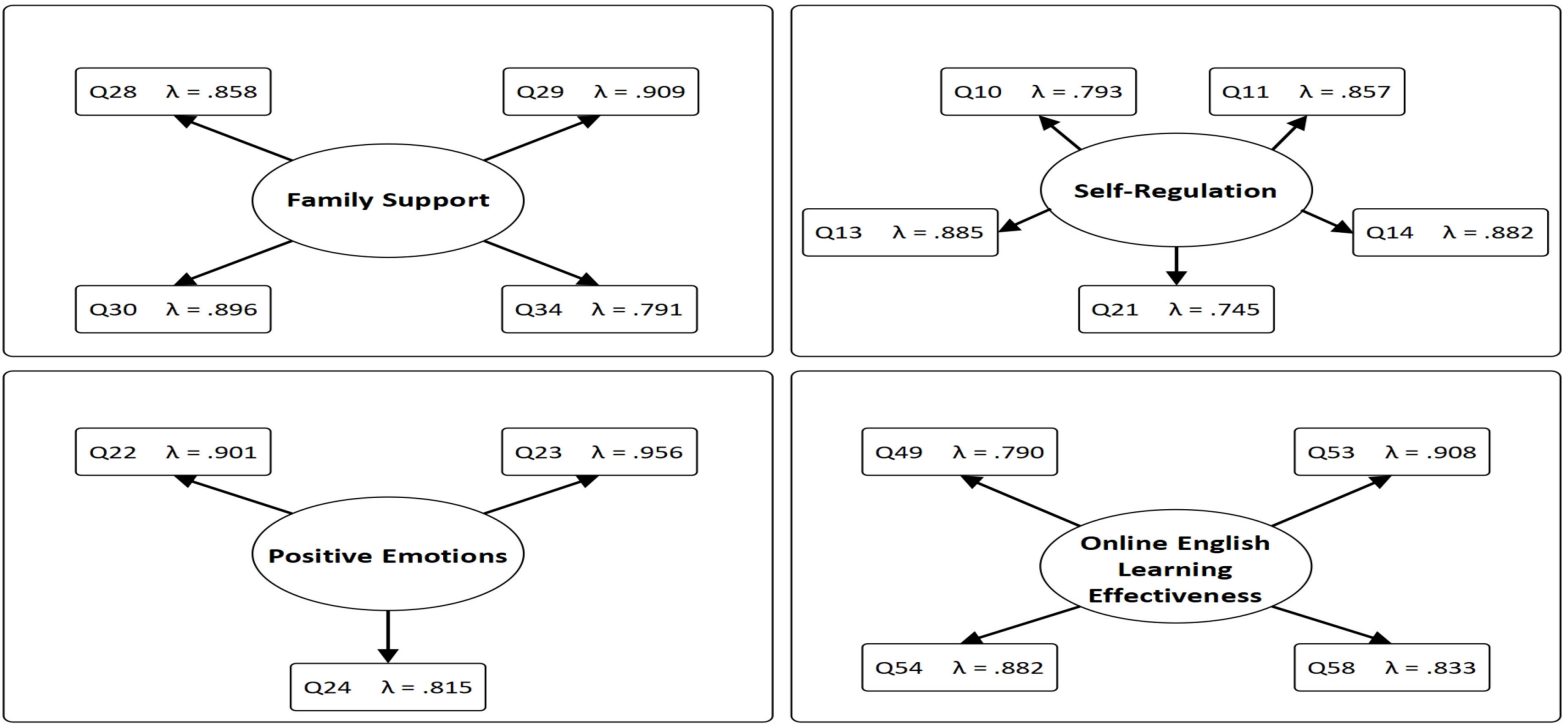
Retained measurement indicators and standardized factor loadings.

Figure 1 and Table 3 present the retained indicators and their measurement coefficients. Standardized factor loadings ranged from 0.745 to 0.956, and squared multiple correlations ranged from 0.555 to 0.914. Composite reliability values ranged from 0.915 to 0.922, and average variance extracted values ranged from 0.696 to 0.797. All non-fixed factor loadings were statistically significant at *p* < 0.001.

As shown in Table 4, the square roots of the average variance extracted values were 0.865 for family support, 0.834 for self-regulation, 0.893 for positive emotions, and 0.854 for online English learning effectiveness. Each value exceeded the corresponding correlations with the other constructs, providing evidence consistent with discriminant validity.

Table 5 presents the model-implied standardized associations among the four constructs. These associations are distinct from the measurement-model correlations reported in Table 4.

**TABLE 5:** Model-implied standardized associations in the final structural model.

Construct pair	Standardized association
Family support and self-regulation	0.562
Self-regulation and positive emotions	0.733
Self-regulation and online English learning effectiveness	0.661
Family support and positive emotions	0.509
Positive emotions and online English learning effectiveness	0.661
Family support and online English learning effectiveness	0.596

Values represent model-implied standardized associations calculated from the final structural model and are presented for descriptive reference. They are distinct from the measurement-model correlations reported in Table 4.

### 4.2 Structural model results

The final structural model showed acceptable fit: CMIN/DF = 1.95, RMSEA = 0.050, GFI = 0.970, AGFI = 0.950, IFI = 0.980, and TLI = 0.970 (Table 6).

**TABLE 6:** Fit indices for the final structural model.

Fit index	Model value
CMIN/DF	1.95
RMSEA	0.050
GFI	0.970
AGFI	0.950
IFI	0.980
TLI	0.970

CMIN/DF, chi-square divided by degrees of freedom; RMSEA, root mean square error of approximation; GFI, goodness-of-fit index; AGFI, adjusted goodness-of-fit index; IFI, incremental fit index; TLI, Tucker–Lewis index.

The approximate explained variance was 31.6% for self-regulation (*R*^2^ ≈ 0.316), 55.1% for positive emotions (*R*^2^ ≈ 0.551), and 55.7% for online English learning effectiveness (*R*^2^ ≈ 0.557).

**FIGURE 2:**
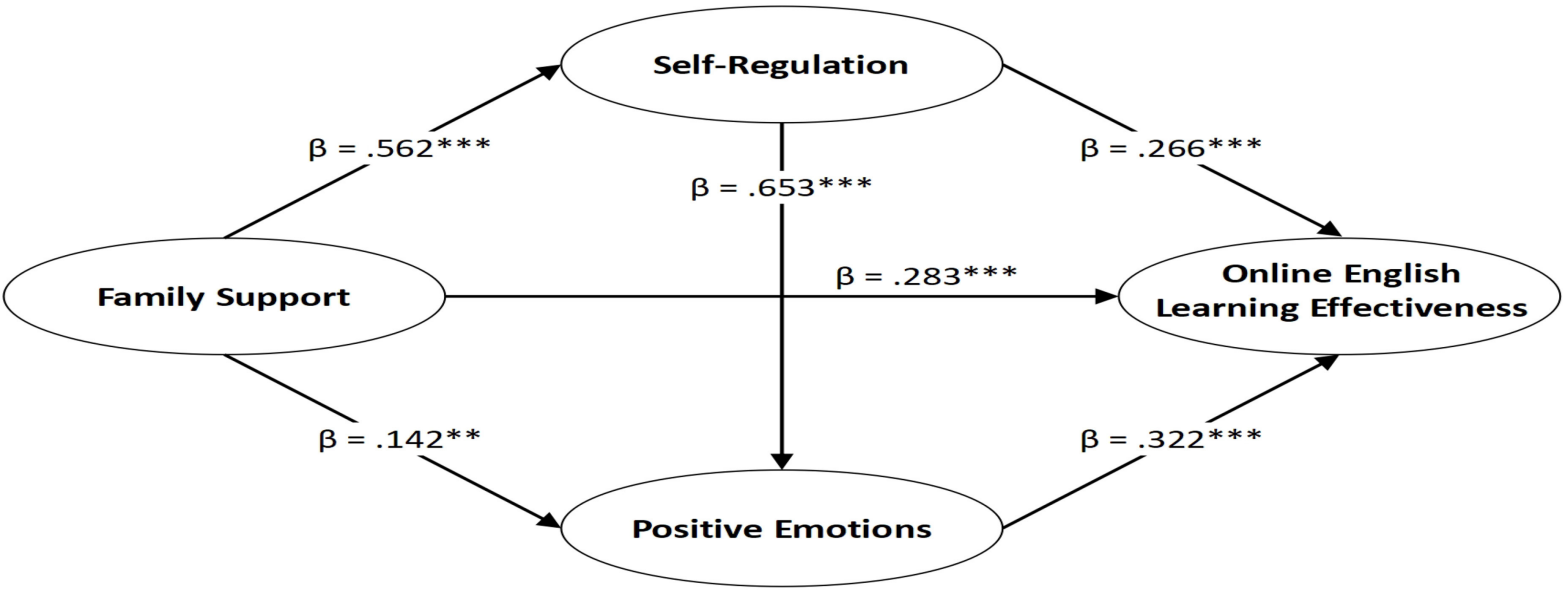
Final structural model with standardized path coefficients. **p < 0.01; ***p <0.001.

The standardized path coefficients are presented in Table 7, and the final structural model is shown in Figure 2. Family support was positively associated with self-regulation, β = 0.562, *p* < 0.001; positive emotions, β = 0.142, *p* = 0.003; and online English learning effectiveness, β = 0.283, *p* < 0.001. Self-regulation was positively associated with positive emotions, β = 0.653, *p* < 0.001, and online English learning effectiveness, β = 0.266, *p* < 0.001. Positive emotions were positively associated with online English learning effectiveness, β = 0.322, *p* < 0.001.

**TABLE 7:** Direct associations and approximate explained variance in the final structural model.

Path relationship	B	β	S.E.	C.R.	*p*
Family support → self-regulation	0.568	0.562	0.053	10.618	< 0.001
Family support → positive emotions	0.150	0.142	0.050	2.986	0.003
Family support → online English learning effectiveness	0.300	0.283	0.052	5.783	< 0.001
Self-regulation → positive emotions	0.680	0.653	0.059	11.449	< 0.001
Self-regulation → online English learning effectiveness	0.280	0.266	0.068	4.130	< 0.001
Positive emotions → online English learning effectiveness	0.326	0.322	0.062	5.260	< 0.001

B, unstandardized coefficient; β, standardized coefficient; S.E., standard error; C.R. critical ratio. Approximate *R*^2^ values are reported in the text and were reconstructed as described in section 3.3.

### 4.3 Specific indirect associations

Table 8 presents the estimates and bias-corrected confidence intervals for the specific indirect associations.

**TABLE 8:** Bootstrap estimates of specific indirect associations.

Association	Estimate	S.E.	Z	Bias-corrected 95% CI		Result
				Lower	Upper	
Specific indirect associations						
Family support → self-regulation → online English learning effectiveness	0.169	0.063	2.683	0.064	0.315	Significant
Family support → positive emotions → online English learning effectiveness	0.046	0.036	1.278	−0.001	0.141	Not significant
Self-regulation → positive emotions → online English learning effectiveness	0.173	0.055	3.145	0.074	0.294	Significant
Family support → self-regulation → positive emotions → online English learning effectiveness	0.110	0.037	2.973	0.050	0.200	Significant

S.E., standard error; BC CI, bias-corrected confidence interval. The self-regulation → positive emotions → online English learning effectiveness pathway is reported to contextualize the proposed sequence; it is not an indirect association originating from family support.

The indirect association between family support and online English learning effectiveness through self-regulation was statistically significant, estimate = 0.169, 95% BC CI [0.064, 0.315].

The indirect association between family support and online English learning effectiveness through positive emotions alone was not statistically significant, estimate = 0.046, 95% BC CI [-0.001, 0.141].

The indirect association between self-regulation and online English learning effectiveness through positive emotions was statistically significant, estimate = 0.173, 95% BC CI [0.074, 0.294].

The sequential indirect association from family support to self-regulation, then to positive emotions, and finally to online English learning effectiveness was statistically significant, estimate = 0.110, 95% BC CI [0.050, 0.200]. This result was consistent with H1.

Taken together, family support was associated with online English learning effectiveness directly, indirectly through self-regulation, and indirectly through the sequential self-regulation–positive-emotions pathway. The indirect association through positive emotions alone was not statistically significant.

## 5 Discussion

### 5.1 Discussion of the findings

This study examined how family support was associated with Chinese university students’ self-reported online English learning effectiveness, both directly and indirectly through self-regulation and positive emotions. It also tested the focused hypothesis concerning the sequential indirect association through self-regulation and positive emotions. Overall, the observed direct and indirect associations were consistent with the proposed environment–cognition–affect–outcome framework, and the significant sequential indirect association was consistent with H1. Given the cross-sectional and self-reported nature of the data, these findings represent theory-consistent associations rather than evidence of causal or temporal processes.

First, family support was positively associated with self-regulation and online English learning effectiveness. Recent research on home-based online English learning has shown that family members can shape learning routines, access to digital tools, and the practical conditions under which online language learning occurs (Qiu et al., 2026). Because that evidence was obtained from parents of young children, it should not be treated as directly comparable with the present university sample. Nevertheless, both studies indicate that family-related resources remain relevant when English learning extends beyond formal classrooms. The present findings suggest that, among university students, family support may be associated with learning through emotional encouragement, autonomy-supportive communication, and access to learning-related resources rather than through direct parental instruction or supervision.

Second, self-regulation was strongly associated with both positive emotions and online English learning effectiveness. This finding is consistent with recent evidence from Chinese university foreign-language learners showing that goal setting, task strategies, and help-seeking strategies are positively associated with online learning engagement (Hui and Wang, 2025). The convergence of these findings supports the interpretation of self-regulation as a proximal process through which students organize goals, strategies, effort, and available resources in flexible digital learning environments. The present study extends this evidence by showing that self-regulation was associated not only with students’ evaluations of learning effectiveness but also with their positive emotional experiences.

Third, positive emotions were directly associated with online English learning effectiveness and formed part of the significant sequential indirect association involving self-regulation. At the same time, the indirect association through positive emotions alone was not statistically significant. This differentiated pattern is compatible with recent meta-analytic evidence showing that psychological-affective outcomes in technology-supported foreign-language learning do not exhibit uniform effects. Whereas learning engagement, motivation, and willingness to communicate showed relatively strong effects, the reported effect on learning enjoyment was smaller (Wang et al., 2026). The present findings similarly indicate that positive emotions are relevant to online English learning but may be more closely associated with favorable learning evaluations when they occur alongside effective self-regulatory management.

The indirect-association results provide a more differentiated account of the role of family support. The significant indirect association through self-regulation indicates that the observed data were consistent with a pathway linking family support to online English learning effectiveness through students’ management of their learning activities. The sequential indirect association through self-regulation and positive emotions was also statistically significant and was consistent with the focused hypothesis. This pattern suggests that family support may correspond with stronger learning management, which in turn is associated with more positive emotional experiences and more favorable evaluations of online English learning effectiveness.

By contrast, positive emotions alone did not account for a statistically significant indirect association between family support and online English learning effectiveness. This result does not imply that positive emotions are unimportant. Positive emotions were directly associated with online English learning effectiveness and were also implicated in the significant association linking self-regulation with online English learning effectiveness. Rather, the differentiated findings suggest that positive emotions may be more closely connected with favorable learning evaluations when they are embedded within students’ self-regulatory management of online learning.

One interpretation is that family support represents a relatively distal socio-contextual resource, whereas self-regulation constitutes a more proximal process for managing online learning demands. Emotional encouragement from family members may correspond with favorable affective experiences, but its association with learning evaluations may be stronger when students translate supportive conditions into concrete learning-management activities, including goal setting, progress monitoring, time management, and strategy adjustment. This interpretation is consistent with the observed sequential indirect association but should remain theory-informed rather than causal.

Overall, the findings underscore the relevance of family support, self-regulation, and positive emotions to understanding university students’ self-reported online English learning effectiveness. In particular, the results position self-regulation as a proximal regulatory process within the observed associations among family support, students’ emotional experiences, and evaluations of online learning. The temporal direction and potential reciprocity of these relationships require further examination through longitudinal, experimental, and multi-source research.

### 5.2 Theoretical contributions

This study offers several theoretical contributions to educational psychology and online language learning. First, it integrates family support, self-regulation, positive emotions, and online English learning effectiveness within a theory-specified environment–cognition–affect–outcome framework. Rather than treating environmental, cognitive, and affective factors as isolated predictors, the framework organizes them as interrelated components of students’ online English learning experiences. Family support was conceptualized as an environmental resource, self-regulation as a cognitive-regulatory process, positive emotions as an affective process, and online English learning effectiveness as a self-reported outcome (Bandura, 1989; Pekrun, 2006; Zimmerman, 2008).

Second, the study highlights family support as a potentially relevant distal socio-contextual resource for university students. Online English learning frequently extends beyond formal classroom settings and requires students to manage learning activities with limited immediate supervision. Under these conditions, perceived emotional encouragement, autonomy-supportive communication, and learning-related resources from family members may be associated with students’ independent management of learning. This contribution does not imply that family support is more important than teacher or peer support. Rather, the study identifies family support as one contextual resource that may extend across courses and learning settings. Future research should examine how family, teacher, peer, and technology-mediated support jointly relate to university students’ online language-learning experiences.

Third, the findings refine the conceptual role of positive emotions in online English learning. Positive emotions were directly associated with online English learning effectiveness and accounted for a significant indirect association between self-regulation and online English learning effectiveness. However, the indirect association from family support to online English learning effectiveness through positive emotions alone was not statistically significant. This pattern suggests that positive emotions may be more closely associated with students’ learning evaluations when they are embedded in self-regulatory processes such as goal management, progress monitoring, effort regulation, and strategy adjustment. Self-regulation may therefore represent a more proximal learning-management process connecting supportive contextual conditions with positive emotional experiences and favorable evaluations of online English learning effectiveness (Pekrun, 2006; Zimmerman, 2008).

These contributions should be interpreted cautiously. The proposed environment–cognition–affect–outcome pathway is theoretically specified and consistent with the observed cross-sectional associations, but it does not establish temporal order or causal direction. Longitudinal and experimental research is required to examine whether these relationships persist over time and whether self-regulation and positive emotions operate reciprocally (Maxwell and Cole, 2007).

### 5.3 Practical implications

The practical implications of this study should be interpreted as theory-informed recommendations rather than causal prescriptions because the findings were based on cross-sectional self-report data.

First, universities and online English instructors may strengthen explicit support for students’ self-regulation. In the present study, self-regulation was strongly associated with both positive emotions and online English learning effectiveness. This finding is consistent with recent evidence from Chinese university foreign-language learners showing that goal setting, task strategies, and help-seeking strategies are positively associated with online learning engagement (Hui and Wang, 2025). Online learning environments may therefore incorporate goal-setting templates, structured study plans, progress-monitoring tools, self-reflection prompts, learning reminders, and accessible help-seeking channels. These forms of support should be designed to develop students’ independent regulation of learning rather than replace it.

Second, instructors may attend to students’ positive emotional experiences while recognizing that affective outcomes vary across digital learning contexts. The present findings showed that positive emotions were associated with online English learning effectiveness and formed part of the significant sequential indirect association involving self-regulation. Recent evidence indicates that technology-supported foreign-language learning has been associated with learning engagement, motivation, enjoyment, willingness to communicate, and self-efficacy, although the magnitude of these relationships varies across outcomes and learning conditions (Fathi et al., 2024; Ouyang et al., 2024; Wang et al., 2026). Practical strategies may include timely and constructive feedback, manageable task sequencing, meaningful opportunities for interaction, recognition of learning progress, and materials aligned with students’ learning goals. Such practices should support students’ perceived control and competence rather than seek only to generate short-term enjoyment.

Third, families may provide emotional encouragement and learning-related resources in ways that respect university students’ autonomy. Recent mixed-methods evidence from home-based online English learning indicates that family practices can shape learning routines, access to digital tools, and the conditions under which online language learning occurs (Qiu et al., 2026). Because that study concerned young children, its findings should not be treated as direct evidence concerning university students. Nevertheless, it demonstrates that family resources can remain relevant when language learning takes place outside formal classrooms. In the university context, appropriate support may include respecting students’ learning schedules, providing reassurance when difficulties arise, and facilitating access to stable internet connections, suitable devices, or appropriate study conditions where possible. Family involvement should support, rather than substitute for, students’ independent learning responsibility.

Fourth, communication between universities and families concerning university students should respect students’ adult status, autonomy, privacy, and informed consent. Universities may provide families with general information about available learning resources, online learning support services, and constructive forms of encouragement. However, information concerning an individual student’s academic performance, online learning behavior, attendance, or emotional experiences should not be disclosed to family members without the student’s explicit consent and in accordance with institutional policies and applicable privacy requirements.

Finally, professional development for online English instructors may include training in the pedagogical use of contemporary digital and artificial-intelligence-supported tools. Recent studies have examined automated and generative feedback in relation to writing engagement, feedback literacy, metacognitive awareness, task motivation, and language-learning outcomes (Rad et al., 2024; Shi et al., 2025; Teng, 2025; Zare et al., 2025). Instructor training may therefore address the design of mastery-oriented tasks, feedback that supports perceived competence, identification of disengagement, and the use of digital tools that promote planning, monitoring, reflection, and persistence. Technology should be treated as a pedagogical resource within a structured learning process rather than as an automatic substitute for instructor guidance or learner self-regulation (Liu and Yao, 2026).

### 5.4 Limitations and future directions

Several limitations should be considered when interpreting the findings. First, the cross-sectional design does not permit conclusions about temporal ordering or causal relationships among family support, self-regulation, positive emotions, and online English learning effectiveness (Maxwell and Cole, 2007). Although the observed associations were consistent with the theory-specified model, self-regulation and positive emotions may operate reciprocally in actual learning processes. Future research should use longitudinal, cross-lagged, diary-based, or experimental designs to examine temporal dynamics and reciprocal relationships more directly.

Second, the study used convenience sampling and snowball referrals. Although participants were recruited from multiple regions of China, the sample should not be regarded as statistically representative of all Chinese university students engaged in online English learning. The number of individuals exposed to the survey invitation was unavailable, and a conventional response rate or formal nonresponse-bias comparison could therefore not be calculated. Selection bias remains possible because students with greater interest in online English learning or greater willingness to participate may have been overrepresented.

Third, first-year students were excluded because the study focused on students with prior university-level online English learning experience. The findings should therefore be generalized cautiously to students at the beginning of university study or learners with limited experience of autonomous online English learning.

Fourth, online English learning effectiveness was assessed through students’ self-reported appraisals of perceived knowledge acquisition, learning progress, goal attainment or application, and satisfaction. The study did not include objective English proficiency tests, course grades, teacher evaluations, or platform-recorded learning behavior. In addition, the measurement model used context-adapted item pools and a retained set of indicators. Although the retained indicators demonstrated satisfactory reliability and validity in the present sample, further validation in independent samples and across different online English learning contexts is required (Boateng et al., 2018).

Finally, all substantive variables were measured using self-report questionnaires completed by the same participants at a single time point. Common method bias and social desirability effects therefore cannot be ruled out, and some observed associations may have been inflated. Future studies should combine self-report measures with teacher evaluations, peer reports, platform-based learning records, objective learning indicators, and temporally separated measurement designs.

## 6 Conclusion

This study examined how family support was associated with Chinese university students’ self-reported online English learning effectiveness, both directly and indirectly through self-regulation and positive emotions. Drawing on social cognitive theory, self-regulated learning theory, and control-value theory of achievement emotions, the study evaluated a theory-specified environment–cognition–affect–outcome model using cross-sectional questionnaire data from 430 participants.

Family support was positively associated with self-regulation, positive emotions, and online English learning effectiveness. Self-regulation was positively associated with both positive emotions and online English learning effectiveness, while positive emotions were positively associated with online English learning effectiveness. The indirect association through self-regulation was statistically significant, and the focused sequential indirect association through self-regulation and positive emotions was also statistically significant, supporting the focused hypothesis. By contrast, the indirect association through positive emotions alone was not statistically significant.

Taken together, the findings identify self-regulation as a proximal regulatory process within the theory-specified model. Family support was associated with students’ evaluations of online English learning both directly and indirectly through self-regulation. Positive emotions were also relevant because they were directly associated with online English learning effectiveness and formed part of the significant sequential indirect association.

The findings highlight the potential relevance of autonomy-supportive family encouragement, explicit support for students’ self-regulation, and online English learning environments that facilitate constructive emotional experiences. Because the study used cross-sectional self-report data and a non-probability sample, the findings should be interpreted as theory-consistent associations rather than evidence of causal or temporal processes. Longitudinal, experimental, and multi-source research is needed to examine the temporal and reciprocal relationships among family support, self-regulation, emotions, and online English learning effectiveness.

## Data Availability

The original contributions presented in the study are included in the article/Supplementary material, further inquiries can be directed to the corresponding author.
