# Supplementary material for: Family support and online English learning effectiveness among Chinese university students: indirect associations through self-regulation and positive emotions
Source: Front Psychol. 2026 Jul 29;17:1866298. doi: 10.3389/fpsyg.2026.1866298 (PMC13462027; doi:10.3389/fpsyg.2026.1866298)
Supplement: Supplementary file 1 [file Supplementary_file_1.docx]

**Appendix 1: Calculation Codes for the total and Indirect Effects Based on family support, self-regulation, and positive emotional pathways**

Function Value( groupNumber As Integer, bootstrapSampleNumber As Integer, v As CValue) As Object Implements IUserValue.Value

Dim x(5) As Double

x(0) = v.ParameterValue("a1") * v.ParameterValue("a2") ' Family support -> Self-regulation -> Online learning effect

x(1) = v.ParameterValue("b1") * v.ParameterValue("b2") ' Family support -> Positive emotions -> Online learning effect

x(2) = v.ParameterValue("a3") * v.ParameterValue("b2") ' Self-regulation -> Positive emotions -> Online learning effect

x(3) = v.ParameterValue("a1") * v.ParameterValue("a3") * v.ParameterValue("b2") ' Family support -> Self-regulation -> Positive emotions -> Online learning effect

' Calculate the direct effect（Family support -> The direct path to the effectiveness of online learning a4）

Dim directEffect As Double = v.ParameterValue("a4")

' Calculate the total effect（Direct effect + All indirect effects）

x(4) = directEffect + x(0) + x(1) + x(2) + x(3)

' Calculate the path difference

Dim diff01 As Double = x(0) - x(1)

Dim diff02 As Double = x(0) - x(2)

Dim diff03 As Double = x(0) - x(3)

Dim diff12 As Double = x(1) - x(2)

Dim diff13 As Double = x(1) - x(3)

Dim diff23 As Double = x(2) - x(3)

Console.WriteLine("Family support -> Self-regulation -> Online learning effect: " & x(0))

Console.WriteLine("Family support -> Positive emotions -> Online learning effect: " & x(1))

Console.WriteLine("Self-regulation -> Positive emotions -> Online learning effect: " & x(2))

Console.WriteLine("Family support -> Self-regulation -> Positive emotions -> Online learning effect: " & x(3))

Console.WriteLine("Direct effect (Family support -> Online learning effect): " & directEffect)

Console.WriteLine("Total effect (Direct effect + Indirect effect): " & x(4))

Console.WriteLine("Difference (x(0) - x(1)): " & diff01)

Console.WriteLine("Difference (x(0) - x(2)): " & diff02)

Console.WriteLine("Difference (x(0) - x(3)): " & diff03)

Console.WriteLine("Difference (x(1) - x(2)): " & diff12)

Console.WriteLine("Difference (x(1) - x(3)): " & diff13)

Console.WriteLine("Difference (x(2) - x(3)): " & diff23)

Dim results(11) As Double

results(0) = x(0) ' Intermediary path 1

results(1) = x(1) ' Intermediary path 2

results(2) = x(2) ' Intermediary path 3

results(3) = x(3) ' Intermediary path 4

results(4) = directEffect

results(5) = x(4)

results(6) = diff01

results(7) = diff02

results(8) = diff03

results(9) = diff12

results(10) = diff13

results(11) = diff23

Return results

**#Region "Advanced"**

Dim labels(11) As String

labels(0) = "Family support -> Self-regulation -> Online learning effect"

labels(1) = "Family support -> Positive emotions -> Online learning effect"

labels(2) = "Self-regulation -> Positive emotions -> Online learning effect"

labels(3) = "Family support -> Self-regulation -> Positive emotions -> Online learning effect"

labels(4) = "Direct effect (Family support -> Online learning effect)"

labels(5) = "Total effect (Direct effect + All indirect effects)"

labels(6) = "Difference (x(0) - x(1))"

labels(7) = "Difference (x(0) - x(2))"

labels(8) = "Difference (x(0) - x(3))"

labels(9) = "Difference (x(1) - x(2))"

labels(10) = "Difference (x(1) - x(3))"

labels(11) = "Difference (x(2) - x(3))"

Return labels

# **Appendix 2:** Interview Materials on the Impact of Family Support on College Students' Online English Learning Outcomes

## Interviewee 1: LN

- **Interview Time**: September 23, 2024
- **Interview Method**: Face-to-face interview
- **Interviewer**: Gao Muyang

**Gao Muyang**:

This interview aims to hear about your experiences and feelings regarding online English learning in the recent semester. The entire process will be anonymous and only used for academic research. You can agree to the recording or request to stop at any time during the interview. Please try to share specific events, such as when they happened, who was involved, what you did, and what the final outcome was.

**Gao Muyang**:

The first question is, could you spend 2 to 3 minutes talking about how you studied English online in the recent semester? Where did you mainly study? What methods did you use? And what is your core goal of learning English?

**LN**:

Regarding the first question, I mainly studied English online in the recent semester through the following methods. First, I memorized about 200 to 300 English words every day. As for the study location, I usually chose the library. I would go there in the morning and start memorizing the required number of words. In terms of learning methods, I initially attended Liu Xiaoyan's English vocabulary courses. Later, I used vocabulary apps on my mobile phone. My core goals for learning English are, first, to prepare for the postgraduate entrance examination, and second, to facilitate my future development, such as pursuing a master's or doctoral degree later on.

**Gao Muyang**:

Okay, what are the most commonly used online English learning platforms, courses, or foreign teacher services for you? And why did you choose them for your study?

**LN**:

The most commonly used platforms for me are vocabulary apps, especially "Bu Bei Dan Ci" (Don't Memorize Words). For courses, I take those taught by Teacher Liu Xiaoyan on Xiao'e Tong, and there are also many foreign teacher services and English courses related to education and training on Bilibili. The reason I chose them is that they are free and offer a large amount of resources.

**Gao Muyang**:

Well, how about your equipment, network connection, and study environment? How do these conditions affect your learning?

**LN**:

My equipment and network do not hinder my learning. The study environment is also very quiet, which has a positive impact on my learning.

**Gao Muyang**:

Looking back on this semester, have there been any incidents where your family's support significantly improved your learning outcomes? Please tell me about it according to the cause of the incident, what your family did, what you did in response, and the final result.

**LN**:

There were both cases where my family provided support and cases where it was more or less negligible this semester. My family gave me financial support and told me to ask them for money whenever I needed it. This relieved me of any financial pressure. Therefore, what did I do? I just buried myself in preparing for the postgraduate entrance examination every day, that's all.

**Gao Muyang**:

In terms of emotion, how can your family's attitude or companionship make you more motivated or persistent in your studies? When did such situations occur, and what changes did you see in your final grades, assignments, or oral English output?

**LN**:

Emotionally, I am quite an independent person, so I don't pay much attention to this aspect. However, my family's attitude has indeed become a motivation for my study, and I rely on myself for the rest.

**Gao Muyang**:

In terms of materials or tools, has your family provided you with equipment, network connection, a dedicated study space, or course funds? Which one has the most direct impact on your learning outcomes? Please give an example.

**LN**:

In terms of materials or tools, I rarely study at home. I usually only study in the school library, so my family didn't provide me with any equipment at home. If we talk about equipment, my learning tools are just a mobile phone, a computer, and some books. There is no dedicated study space at home except for my bedroom. My family did provide support for course funds. Which one has the most direct impact on my learning outcomes? Let me think. Well, the living expenses have the most direct impact.

**Gao Muyang**:

In terms of learning methods or plans, has your family made study plans with you, checked your progress, or practiced oral English with you? Tell me a story where a change in learning methods led to a change in learning outcomes.

**LN**:

In terms of learning methods and plans, my family has not made any plans with me because they have relatively low educational backgrounds and don't know much about these things. However, they usually ask me about my review progress and how many questions I have done when we talk on the phone. This more or less plays a supervisory role. But it didn't change my learning methods or outcomes.

**Gao Muyang**:

In terms of rules, are there any family agreements regarding time management or mobile phone use? How are these rules implemented? What impact do they have on your learning?

**LN**:

There are no family agreements on time management or mobile phone use. If there were any, they would date back to my junior high school and high school years, when my family might have restricted my mobile phone use, but they couldn't really control me. Now, there are even fewer such rules, so they have no impact on me at all.

**Gao Muyang**:

If you hadn't received this support or if the support had been reduced, how do you think the outcome would have been different? Why?

**LN**:

I don't think the outcome would have been much different. Because I believe that learning mainly depends on oneself, and these things don't have a particularly significant impact.

**Gao Muyang**:

Have you noticed that differences in family support among classmates or friends have led to gaps in their learning? Please give an example from your身边.

**LN**:

I think there are such cases. For example, one of my junior high school classmates, whose parents are teachers and whose father holds the position of county secretary. He was almost the top student in the school during junior high school. Then, he continued to achieve excellent grades throughout high school and was admitted to a very prestigious university. I think this gap does exist to some extent and can make up for certain gaps in educational resources.

**Gao Muyang**:

Please talk about a complete process of your preparation for an exam or learning a certain program. How did you set goals, arrange time, check progress, and review knowledge?

**LN**:

Actually, I don't have a well - thought - out plan for my exam preparation process. As a science student, my general plan is to finish my exercise book and complete the first round of practice by a certain month. On a daily basis, I set a goal of finishing a certain number of pages, but sometimes it's hard to achieve because the amount of questions is really large. In terms of time arrangement, I spend almost the whole day studying. Sometimes I spend a lot of time on English learning, and sometimes I spend almost no time on it. I spend the whole day doing math exercises. When it comes to checking progress and reviewing knowledge, I think the sooner I finish a round of study, the better. Because the real gains come in the second and third rounds of review. For knowledge review, I first review a unit and then go through the knowledge of that unit again. That's my way of reviewing knowledge.

**Gao Muyang**:

What are the three most common distractions you encounter while studying? For example, scrolling through your phone, noise, or a backlog of tasks. How do you specifically deal with them?

**LN**:

Among the three types of distractions, scrolling through my phone is not a distraction for me. I scroll through my phone mainly when I'm too sleepy to stay awake. Scrolling helps me stay awake. During my postgraduate exam preparation, I have trouble sleeping, so sometimes I don't want to sleep in the library. That's why I choose to scroll through my phone to help me fall asleep quickly at night. Noise is relatively rare. A backlog of tasks does affect my study. For example, the tasks arranged by the school and the postgraduate exam preparation will definitely have an impact. How do I deal with them? For scrolling through my phone, I sometimes restrain myself by deleting some video apps on my phone. For the backlog of tasks, I try my best to avoid them. I communicate online as much as possible. If a problem can be solved online, I solve it directly. If it can't be solved online, I solve it quickly offline. That's how I handle it.

**Gao Muyang**:

Have you ever had an experience where you had good self - control and achieved significantly better results? What methods did you use at that time, how long did you use them, and what changes did you finally achieve?

**LN**:

I don't have much experience in this aspect. If there is an experience where the result was slightly better, it might be when I was preparing for the Teacher Qualification Examination. I went through all the questions of the past five years, including both objective and subjective questions. Then, I passed the exam on my first try. So, this might be an experience of achieving better results, and the method was just doing a lot of practice questions.

**Gao Muyang**:

Please also talk about an experience where you had poor self - control. What happened to you at that time? How did you adjust yourself later?

**LN**:

As for me, I have always had relatively poor self - control. However, I think exercising is a good way for me to adjust myself. From March to June, I went to the gym almost every day. This helped me relieve the pressure of study. This may also be something I persisted in and controlled well for myself.

**Gao Muyang**:

When learning English online, when do you clearly feel that it is interesting, engaging, enjoyable, or makes you confident? What situations usually trigger these emotions? For example, anonymous chatting, being able to watch replays, setting your own pace, or receiving feedback from teachers.

**LN**:

When learning English online, I clearly feel it is interesting when the teacher tells some interesting stories and shares his experiences. I think these are inspiring for us learners. Second, when I am doing exercises and find that my correct rate is relatively high, it also gives me positive feedback.

**Gao Muyang**:

Talk about an event where positive emotions emerged, what you did as a result, and how your learning outcomes changed in the end.

**LN**:

This kind of situation is relatively rare for me, but if I have to talk about one, I can say a little. It was when I was doing practice questions that positive emotions emerged. For example, when the teacher affirmed me and had expectations for me, I would move towards the goal of getting into the school I wanted. For example, on a certain day, I finally completed the heavy task volume. This was also a change in my learning outcomes at a small stage.

**Gao Muyang**:

Did you become more willing to participate or speak up because online learning didn't require face - to - face interaction? What did you do then, and what differences did you see in your assignments, tests, or oral English performance?

**LN**:

I hardly speak up during online learning because I only watch replays. So, I didn't do anything in this aspect.

**Gao Muyang**:

On the contrary, what situations make you feel anxious, bored, or tired? How do you usually adjust yourself to get back to a good learning state?

**LN**:

There are some situations that make me feel tired. First, I almost can't finish the daily task volume because it's really large. So, I feel tired. Second, I feel quite anxious every day now. The countdown to December 20th is less than 99 days. So, I feel anxious every day. How do I usually adjust? If I feel too anxious one day, my learning effect the next day will decrease. I usually play a few games with my roommates, my elementary school classmates, and my postgraduate exam study partners from 11 p.m. to 12 p.m. in the dormitory. This allows me to release some pressure and chat with them.

**LN**:

Second, I learn to give myself a holiday. I usually give myself a holiday after studying for a week. During the summer vacation, I often gave myself holidays. At that time, I almost took two holidays a week. Usually, I studied until about 5 p.m. on the holiday, and then I didn't study from 5 p.m. to night. I gave myself a holiday.

**Gao Muyang**:

Please tell a complete chain story for each of the following two scenarios. The first one is about how family support leads to changes in self - control ability and then affects your learning outcomes. The second one is about how family support leads to an improvement in positive emotions and then affects your learning outcomes.

**LN**:

I don't really have a story for the first one. For the second one, family support does bring about an improvement in positive emotions. As for its impact on my learning outcomes, we can only wait and see in a few months.

**Gao Muyang**:

Based on your experience, which of these two chains is more important to you? Why?

**LN**:

Based on my experience, which chain is more important to me? First of all, family support should be the most important. If your family opposes you taking the postgraduate entrance exam or doing other things, you will definitely have negative emotions, which will definitely have a negative impact on your learning outcomes. Second, I think self - control ability is also very important. Because without self - control, you will keep playing. Next, equally important are sticking to your goals and having the determination to keep going.

**Gao Muyang**:

Will these two chains affect each other? For example, being more self - disciplined first makes you feel better, or having a better mindset first makes it easier to persist. Please use a specific event to explain.

**LN**:

I think these two chains will affect each other. On the one hand, your family's support will definitely promote your learning. On the other hand, your self - discipline, the firmness of your goals, and your determination will be further consolidated and improved with the promotion of your family. Therefore, it will definitely play a guiding role in your learning goals. Let's take a specific example. For instance, when my family calls me and asks about my study progress and how many questions I have done. Then they talk about my mother's colleague whose daughter was admitted to Wuhan University of Technology and other 211 universities. This certainly has a promoting effect on me. It also affects my choice of schools for the postgraduate entrance exam, and then I have some thoughts about wanting to get into 985 or 211 universities.

**Gao Muyang**:

If we summarize your experience as "family support affects self - control ability and positive emotions, which in turn affect the effectiveness of online English learning", do you think this is in line with your situation?

**LN**:

I don't think it is quite in line with my situation. Throughout my learning journey, I have basically relied on myself. My family has given me a lot of freedom and doesn't restrict me much. Family support does bring some positive effects, but it is not the most important factor.

**Gao Muyang**:

Are there any key factors in your experience that we haven't mentioned yet? For example, classmates, teacher feedback, the difficulty of the courses, or how you evaluate these factors. How do they affect your learning?

**LN**:

I think there are such factors. For example, classmates. The learning progress of my postgraduate exam study partners will cause me to feel anxious and also promote my learning. In addition, the experience posts of students who have been admitted to postgraduate programs can help me avoid detours on my learning path. Teacher feedback is also crucial. If you have a good teacher, it's like having a good beacon. I regard my teacher as a guide for my future path.

**LN**:

I think the difficulty of our courses is at the top level. How to evaluate this? I think if the courses are difficult, you mainly have to think more and try to figure out the problems. When you encounter difficult things, don't avoid them. After overcoming them, go back and solve those difficult problems. Because dealing with these course - related problems is also a reflection of how we will solve problems in our future lives, such as encountering difficulties in scientific research and solving them.

**Gao Muyang**:

If you were to rank them, what are the three most critical factors for you? And please explain your reasons.

**LN**:

If I have to rank the three most critical factors, the first one should be my own life planning. The second factor is family support, and the third factor is the desire to gain recognition.

**Gao Muyang**:

What online English platforms or functions have you used? For example, self - paced learning, personalized recommendations, live foreign teacher classes, small - group interactive learning, or gamified rewards. Please explain why you chose them.

**LN**:

On the one hand, I like personalized recommendations because they are more flexible and free. I think my self - control is quite good, so I think personalized recommendations are suitable for me. Live foreign teacher classes are not suitable for me. Sometimes I get distracted when watching live classes. Moreover, if I can't watch the replay of the live class, I think the effect is not as good as that of personalized recommendations. Although online learning provides convenience, there are many sources of interference in the online learning environment, such as social media and entertainment content, which can distract me and affect my learning effect.

**Gao Muyang**:

Choose a function that you use most frequently or that leaves the deepest impression on you. Talk about how it changed your goals, progress, mood, or way of participation, and what learning outcomes it finally brought about.

**LN**:

The function that I use most frequently and that has left the deepest impression on me and may have changed my learning goals is the various experience posts on my mobile phone. These experience posts have indeed helped me better plan my goals and progress. They also remind me to stay focused when I feel slack, and make me more actively involved in my study.

**Gao Muyang**:

Have these technologies ever brought about side effects? Are there any drawbacks? For example, distracting your attention, having too much information, or causing anxiety. How do you balance their advantages and disadvantages?

**LN**:

I think the disadvantage of having too much information is that it reduces my learning concentration. Sometimes I spend a lot of time reading these experience posts, which really affects my learning focus. However, I don't think anxiety is a disadvantage for me. I believe that people will pursue progress more only when they are under pressure. For example, a fountain can spray water because it is under pressure, and a waterfall is the same.

**Gao Muyang**:

Compared with your classmates around you, what advantages or limitations do you have in terms of equipment, network connection, study location, access to courses or foreign teacher services, and your family's attitudes?

**LN**:

I think I have no limitations in terms of courses, network connection, and study location, but I have limitations in terms of access to foreign teacher services.

**Gao Muyang**:

How do these differences lead to gaps in learning outcomes at critical times, such as during exam preparation, important assignments, or projects? Please give an example.

**LN**:

I think there will be such gaps. For example, access to foreign teacher services will definitely make your English, at least your oral English, more fluent. This will affect your English communication in the interview part of the postgraduate entrance exam re - examination.

**Gao Muyang**:

Have you observed any cases where learning gaps were made up through effective methods or support? How was this achieved?

**LN**:

I think there are such cases. For example, one of my high school classmates signed up for an online training course on Gaotu (a well - known online education platform) and spent tens of thousands of yuan. Originally, his grades were average, but he achieved good results in the college entrance examination later.

**Gao Muyang**:

Based on your experience, please give one most practical suggestion that students, parents, schools, or platforms can try immediately. Explain what to do, how to do it, and how often to do it.

**LN**:

For students, the suggestion is to exercise regularly. After all, good health is the foundation of all endeavors. So, I think this should be a suggestion: do more exercise, such as running or going to the gym. You can try to exercise three or four times a week, which won't have a significant impact on your study. I don't have any particularly good suggestions for parents. If there must be one, it is that if your child is self - disciplined, conscious, and motivated, you should give them more freedom. For schools, I think they can better improve the construction of public service facilities to make it more convenient for students. I don't have much to say about platforms.

**Gao Muyang**:

If you were to learn English online again next semester, what three things would you change first? Why would you change them?

**LN**:

I don't think I would change anything about my online English learning. Because I basically rely on some ordinary learning methods to learn English, so I don't have anything to change in online learning.

**Gao Muyang**:

Well, let me briefly repeat the key points you shared today. When learning English, you mainly learn through online English replays or vocabulary apps. You believe that family support has a promoting effect on you, but you mainly rely on yourself.

**Tao Junlong**:

Yes, that's correct.

**Gao Muyang**:

Finally, after the interview record is completed, if needed, would you be willing to help check the important content or supplement materials?

**Tao Junlong**:

Of course, I am willing.

## Interviewee 2: Student Li

- **Interview Time**: September 23, 2024
- **Interview Method**: Face-to-face interview
- **Interviewer**: Gao Muyang

**Gao Muyang**:

The purpose of this interview is to hear about your experiences and feelings of learning English online in the recent semester. The whole process will be anonymous and only used for academic research. You can agree to the recording or stop at any time during the interview. Please try to use specific events to explain, such as when it happened, who was involved, what you did, and what the final result was.

**Gao Muyang**:

The first question: could you spend 2 to 3 minutes talking about how you learned English online in the recent semester? Where did you mainly study? What methods did you use? And what is your core goal of learning English?

**Student Li**:

I mainly learned English online in my dormitory. The methods I used included listening to English listening materials, doing reading exercises, and occasionally reading oral English aloud along with PPT. Later, my parents saw that I liked English and enrolled me in an online English course. My core goal is to improve my comprehensive English ability. After all, although the civil service examination mainly focuses on Administrative Aptitude Test and申论 (a type of essay - writing test), some positions and examinations may involve English. Moreover, I think learning English will be beneficial for my future work and help me make preparations.

**Gao Muyang**:

What are the most commonly used online English learning platforms, courses, or foreign teacher services for you? Why did you choose them?

**Student Li**:

The most commonly used ones are the "Meiri Ting Yingyu Tingli" (Daily English Listening) app and "Bohe Yuedu" (Mint Reading).

**Student Li**:

I use "Daily English Listening" because it has abundant resources, including various listening materials such as BBC, and it allows adjusting the playback speed. I chose "Mint Reading" because it helps me accumulate vocabulary and improve my reading ability, which is very convenient.

**Gao Muyang**:

How about your equipment, network connection, and study location? How do these conditions affect your learning?

**Student Li**:

My equipment includes my mobile phone and a tablet computer. The network connection is either my mobile phone's mobile hotspot or the campus network, which is relatively stable. I study in the dormitory or the study room of the library, which is very quiet. These conditions are quite good. A stable network allows me to learn English and prepare for the civil service examination more smoothly. A quiet environment also helps me concentrate more on my studies, and my equipment can meet the needs of online learning.

**Gao Muyang**:

Looking back on this semester, have there been any incidents where your family's support significantly improved your learning outcomes? Please tell me about it according to the cause of the incident, what your family did, what you did in response, and the final result.

**Student Li**:

The cause was that I was under great pressure while preparing for the civil service examination. However, I had already enrolled in an English course, and it would be a waste if I didn't study it. Sometimes, I couldn't focus on learning English and felt irritable. After I shared this with my family, they would give me psychological counseling and chat with me occasionally when we made phone calls every day. As a result, I felt that my pressure was relieved, and I could calm down more when learning English.

**Gao Muyang**:

In terms of emotion, how can your family's attitude or companionship make you more motivated or persistent in your studies? When did such situations occur, and what changes did you see in your final grades, assignments, or oral English output?

**Student Li**:

My family's companionship and encouragement can make me more motivated. Once, when I went home during the holiday, I was practicing my oral English, but I always got stuck and felt frustrated. My parents encouraged me, saying that I was already doing a great job and that I would definitely get better with practice. Both my father and mother were very supportive. Sometimes, my mother even practiced oral English with me. Since then, I have persisted in practicing oral English every day. A week later, my oral English became significantly more fluent in the online oral English test.

**Gao Muyang**:

In terms of materials or tools, has your family provided you with equipment, network connection, a dedicated study space, or course funds? Which one has the most direct impact on your learning outcomes? Please give an example.

**Student Li**:

My family has provided me with equipment, network connection, and course funds. I have enrolled in both online and offline English courses. The course funds have the most direct impact on my learning outcomes. Because after enrolling in the course, I can get guidance from more professional teachers and learn in a more targeted way. For example, the teacher will make a special study plan for me according to my situation and teach me some exam - taking skills. I followed the teacher's learning methods, and my learning effect improved rapidly. Moreover, it is very convenient to consult teachers now. I can book language teachers from all over the world through online platforms.

**Gao Muyang**:

In terms of learning methods or plans, has your family made study plans with you, checked your progress, or practiced oral English with you? Tell me a story where a change in learning methods led to a change in learning outcomes.

**Student Li**:

I didn't have a specific study plan for learning English before, but the course I enrolled in had a plan, so my family didn't make one for me.

**Gao Muyang**:

In terms of rules, are there any family agreements regarding time management or mobile phone use? How are these rules implemented? What impact do they have on your learning?

**Student Li**:

There is a family agreement on time management. I fix my English learning time between 1 and 2 hours every day. I can't use my mobile phone for non - study purposes except when searching for materials during study time. My mother will supervise me, but when I am at school, I can only supervise myself. These rules about mobile phone use help me concentrate more on learning English and preparing for the civil service examination. I won't be distracted by playing with my mobile phone, and my learning efficiency has improved.

**Gao Muyang**:

If you hadn't received this support or if the support had been reduced, how do you think the outcome would have been different? Why?

**Student Li**:

If I hadn't received this support, I think my learning effect would have been much worse. Because my family's support can give me motivation and help. For example, the course funds allow me to receive better education, the time management agreement helps me become more self - disciplined, and my family's companionship can relieve my pressure. Without these, I might not be able to focus on my studies.

**Gao Muyang**:

Have you noticed that differences in family support among classmates or friends have led to gaps in their learning? Please give an example from your身边.

**Student Li**:

One of my classmates' families doesn't support him in learning English, thinking that it's useless for the civil service examination. He himself also lacks motivation, so he hasn't made much progress in English. With my family's support, I have learned English well. There is a gap between us. Even if I don't pass the civil service examination, I can still have a competitive advantage in other positions. That's why I keep persisting in learning English.

**Gao Muyang**:

Please talk about a complete process of your preparation for an exam or learning a certain program. How did you set goals, arrange time, check progress, and review knowledge?

**Student Li**:

While preparing for the civil service examination and learning English at the same time, my goal is to complete the planned tasks every day. In terms of time arrangement, I fix 1 to 2 hours every day for English learning. I spend one hour on listening and oral English in the morning, and one hour on reading and writing in the evening. However, there are sometimes special circumstances. To check my progress, I take a mock test every week to see if my score has improved. For review, after finishing the exercises each time, I sort out the wrong questions, analyze the reasons, review the relevant knowledge points, and mark the unfamiliar words for review.

**Gao Muyang**:

What are the three most common distractions you encounter while studying? For example, scrolling through your phone, noise, or a backlog of tasks. How do you specifically deal with them?

**Student Li**:

In fact, the most common distractions I encounter are my family asking me to do things at home sometimes, the urge to scroll through my phone, and occasional gatherings with friends. To deal with these, I usually control my study time well. Sometimes I make use of fragmented time for learning to avoid wasting time. When studying, I turn off my mobile phone or put it in another place and enable the focus mode. If I have other things I want to do, I write them down first and do them after finishing my study.

**Gao Muyang**:

Have you ever had an experience where you had good self - control and achieved significantly better results? What methods did you use at that time, how long did you use them, and what changes did you finally achieve?

**Student Li**:

During the summer vacation, I enrolled in an offline training course for the civil service examination. I studied according to the course schedule and plan made by the class every day, got up and went to bed on time. I felt that my physical condition improved, and my learning efficiency also became much better. Although the course only lasted for half a month, I learned many useful methods, which were more beneficial to my study.

**Gao Muyang**:

Please also talk about an experience where you had poor self - control. What happened to you at that time? How did you adjust yourself later?

**Student Li**:

Once, I had poor self - control. While studying, I couldn't help but scroll through short videos on Douyin. I picked up my mobile phone to watch videos every now and then. Later, I uninstalled the Douyin app from my mobile phone. Then I used the Tomato Timer app to force myself to study, recorded my effective study time, and joined a study group where we could supervise each other. Gradually, I adjusted myself back to a good learning state.

**Gao Muyang**:

When learning English online, when do you clearly feel that it is interesting, engaging, enjoyable, or makes you confident? What situations usually trigger these emotions? For example, anonymous chatting, being able to watch replays, setting your own pace, or receiving feedback from teachers.

**Student Li**:

When learning English online, I clearly feel happy, engaged, and confident when I communicate with other learners in anonymous chats, especially when I can communicate smoothly in English. In addition, when the teacher gives me timely feedback, points out my problems, and praises my progress, I will have these positive emotions.

**Gao Muyang**:

Talk about an event where positive emotions emerged, what you did as a result, and how your learning outcomes changed in the end.

**Student Li**:

Once during the holiday, my cousin came to my home. He had some English questions that he couldn't answer, so I taught him. I felt a great sense of accomplishment and thought that my English was quite good, which made me very happy.

**Gao Muyang**:

Did you become more willing to participate or speak up because online learning didn't require face - to - face interaction? What did you do then, and what differences did you see in your assignments, tests, or oral English performance?

**Student Li**:

I think yes. Because I am not a very outgoing person. At school, I usually wait for the teacher to call my name before answering questions. But online, since all the classmates are strangers and I can communicate by typing, I am more willing to interact.

**Gao Muyang**:

On the contrary, what situations make you feel anxious, bored, or tired? How do you usually adjust yourself to get back to a good learning state?

**Student Li**:

When I encounter very difficult articles or have a hard time memorizing things while studying, I will feel anxious or very tired. I usually do some other relaxing things first, such as listening to music or going out for a run. After relaxing, I go back to study. Usually, this way I can get back to a good learning state.

**Gao Muyang**:

Please tell a complete chain story for each of the following two scenarios. The first one is about how family support leads to changes in self - control ability and then affects your learning outcomes. The second one is about how family support leads to an improvement in positive emotions and then affects your learning outcomes.

**Student Li**:

First, let's talk about the first chain. My family provided me with a comfortable learning environment. Although I usually study at school, going home during holidays is also a good opportunity to make progress beyond others. With the support of my parents, my self - control ability has become stronger, and I can concentrate more on my studies. Second, for the second chain, sometimes I encounter a bottleneck in my study and feel frustrated. I will call my parents, and they will always encourage me, telling me not to be too anxious and that they will always be there for me. Then my mood improves, I become more positive, and I have more motivation to study.

**Gao Muyang**:

Based on your experience, which of these two chains is more important to you? Why?

**Student Li**:

The chain of improving positive emotions is more important to me. Because when I have positive emotions, I will have more motivation to study and can persist longer. And family support is an important source of my positive emotions.

**Gao Muyang**:

Will these two chains affect each other? For example, being more self - disciplined first makes you feel better, or having a better mindset first makes it easier to persist. Please use a specific event to explain.

**Student Li**:

They will affect each other. For example, once I controlled the time I spent on my mobile phone. I made a rule that I could only use my mobile phone after completing my tasks, and I also locked my mobile phone. So, my learning effect that day was very good, and I felt even better. When I am in a good mood, it is easier for me to persist in studying, and I can maintain a good level of self - control.

**Gao Muyang**:

If we summarize your experience as "family support affects self - control ability and mood, which in turn affect the effectiveness of online English learning", do you think this is in line with your situation?

**Student Li**:

I think it is quite in line with my situation. Because my family's support makes me more self - disciplined in my studies and in a better mood, which both promote the effectiveness of my online English learning.

**Gao Muyang**:

Are there any key factors in your experience that we haven't mentioned yet? For example, classmates, teacher feedback, the difficulty of the courses, or how you evaluate these factors. How do they affect your learning?

**Student Li**:

The mutual communication between classmates is also very crucial. I set up a study communication group with several classmates. We often discuss problems and share learning materials together. This allows me to learn from others' learning methods and consolidate my own knowledge through communication, which is of great help to my study.

**Gao Muyang**:

If you were to rank them, what are the three most critical factors for you? And please explain your reasons.

**Student Li**:

The first one is family support, because it provides me with motivation and security. The second one is a suitable learning method, because a good learning method can help me study efficiently. The third one is communication between classmates, which allows us to learn from each other and make progress together.

**Gao Muyang**:

What online English platforms or functions have you used? For example, self - paced learning, personalized recommendations, live foreign teacher classes, small - group interactive learning, or gamified rewards. Please explain why you chose them.

**Student Li**:

I have used the personalized recommendation function of "Daily English Listening" and online live courses. I chose the personalized recommendation function of "Daily English Listening" because it can recommend suitable listening materials according to my learning situation, saving me the time of looking for materials. I chose online live courses because I can communicate with foreign teachers in real time to practice my oral English and listening skills. When I don't have time to watch the live broadcast, I will watch the replay.

**Gao Muyang**:

Choose a function that you use most frequently or that leaves the deepest impression on you. Talk about how it changed your goals, progress, mood, or way of participation, and what learning outcomes it finally brought about.

**Student Li**:

In terms of English learning, it is mainly the personalized recommendation function of "Daily English Listening". I really think it is very good. It can not only be used for learning listening but also for listening to civil service examination courses. In the past, I spent a lot of time looking for English listening materials, but now the materials it recommends are very suitable for me, which enables me to study more efficiently.

**Gao Muyang**:

Are there any drawbacks to these technologies? For example, distracting your attention, having too much information, or causing anxiety. How do you balance their advantages and disadvantages?

**Student Li**:

These technologies do have drawbacks. For example, in "Daily English Listening", there is too much information, and sometimes I am distracted by it. My way of balancing the advantages and disadvantages is to focus on only one learning task at a time. For example, I only listen to listening materials and turn off the app when I don't listen, so as to avoid being distracted by other information.

**Gao Muyang**:

Compared with your classmates around you, what advantages or limitations do you have in terms of equipment, network connection, study location, access to courses or foreign teacher services, and your family's attitudes?

**Student Li**:

In terms of equipment and network connection, I am almost the same as my classmates around me, and we can all meet the needs of online learning. In terms of access to courses or foreign teacher services, I have enrolled in an online course with the support of my family, which provides me with better learning resources. This is my advantage. My family's attitude is to support my study and hope that I can improve myself. They support my ideas every time.

**Gao Muyang**:

How do these differences lead to gaps in learning outcomes at critical times, such as during exam preparation or important projects? Please give an example.

**Student Li**:

I think there are such gaps. Some classmates' parents support them in taking the civil service examination, but they don't provide much practical support. This is a waste of time and energy, and their learning conditions are more difficult, so their learning efficiency may not be as high as mine. However, with the support of my parents, I have more abundant learning resources. I think this is my advantage.

**Gao Muyang**:

Have you observed any cases where learning gaps were made up through strategies or support? How was this achieved?

**Student Li**:

One of my classmates found it very difficult to study at first, which made him hate studying and he was in a slump for a period of time. Later, I found out that his parents wanted him to work in his relative's factory directly after graduation, but he wanted to take the civil service examination. So he didn't have his parents' support. Later, his parents finally respected his opinion. After learning about the civil service examination, they became more and more supportive of him. So he quickly caught up with the progress later.

**Gao Muyang**:

Based on your experience, please give one most practical suggestion that students, parents, schools, or platforms can try immediately. Explain what to do, how to do it, and how often to do it.

**Student Li**:

For students, make a study plan every day and strictly implement it. For example, fix 2 hours every day for English learning and make a summary once a week. For parents, encourage and support their children more. When their children encounter difficulties in study, give them care and help, and communicate with their children about their study situation once a week. For platforms, optimize the personalized function to recommend more suitable learning content for learners more accurately.

**Gao Muyang**:

If you were to learn English online again next semester, what three things would you change first? Why?

**Student Li**:

First, I will improve my study plan to make it more in line with my learning rhythm. Second, I will be more bold and increase the frequency of communication with foreign teachers to improve my oral English. Finally, I will make more reasonable use of the resources of online learning platforms to improve my learning efficiency. Because I think there is still room for optimization in my current English study plan, my oral English needs to be strengthened, and there are still many resources on the platform that I haven't fully utilized.

**Gao Muyang**:

Now, let me briefly repeat the key points you shared today. Please see if there is anything incorrect or missing. The key points you summarized are probably as follows: family support is very important for online English learning, self - control and positive emotional value also affect learning outcomes, and you also mentioned the help of communication with classmates.

**Student Li**:

There is nothing missing.

**Gao Muyang**:

After the interview record is completed, if needed, would you be willing to help check the important content or supplement materials?

**Student Li**:

I am very willing to help if needed.

**Gao Muyang**:

Okay, thank you. Wish you a happy life!

## Interviewee 3: Student Han

- **Interview Time**: September 21, 2024
- **Interview Method**: Face-to-face interview
- **Interviewer**: Gao Muyang

**Gao Muyang**:

The purpose of this interview is to hear about your experiences and feelings of learning English online in the recent semester. The whole process will be anonymous and only used for academic research. You can refuse the recording or request to stop at any time during the interview. Please try to answer with specific events, such as when it happened, who was involved, what you did, and what the final result was.

**Gao Muyang**:

The first question: could you spend 2 to 3 minutes talking about how you learned English online in the recent semester? Where did you mainly study? What methods did you use? And what is your core goal of learning English?

**Student Han**:

In the recent semester, I mainly studied English online through online courses and apps. I mainly studied in the school dormitory or the library. As an English major student, my goal is to pass the TEM - 8 (Test for English Majors - Band 8) examination.

**Gao Muyang**:

What are the most commonly used online English learning platforms, courses, or foreign teacher services for you? Why did you choose them?

**Student Han**:

The most commonly used one is the "Bu Bei Dan Ci" (Don't Memorize Words) app. Because it has a pronunciation correction function and many scenario - based vocabulary exercises, which allows me to know where my pronunciation is wrong. It can also make a vocabulary plan for me according to my memory forgetting curve. In addition, I also enrolled in an online English intensive course. Because the TEM - 8 examination is very difficult, and I want to get a higher score, which will be of great help to my job hunting.

**Gao Muyang**:

How about your equipment, network connection, and study location? How do these conditions affect your learning?

**Student Han**:

I use a laptop. I study in the study room of the library or at a desk outside. When the network is stable, the classes go very smoothly. If there is a network jam, I will connect to my mobile phone's mobile hotspot. These learning conditions are very good. The study room also has air conditioning and is very quiet.

**Gao Muyang**:

Looking back on this semester, have there been any incidents where your family's support significantly improved your learning outcomes? Please tell me about it according to the cause of the incident, what your family did, what you did in response, and the final result.

**Student Han**:

Yes. I participated in a teaching speech contest this semester and was not confident at first. After my family found out, my parents always encouraged me and told me not to be too nervous. In the end, I didn't want to let them down and got a pretty good ranking.

**Gao Muyang**:

In terms of materials or tools, has your family provided you with equipment, network connection, a dedicated study space, or course funds? Which one has the most direct impact on your learning outcomes? Please give an example.

**Student Han**:

My family provided me with a laptop, network connection, and an independent study space. They also enrolled me in an online course for me. This has the most direct impact on my learning, because the course content is very useful for my study, and the price is a bit expensive. Without their support, it would be difficult for me to have this opportunity.

**Gao Muyang**:

In terms of learning methods or plans, has your family made study plans with you, checked your progress, or practiced oral English with you? Tell me a story where a change in learning methods led to a change in learning outcomes.

**Student Han**:

Since my mother is also a teacher, once she made a vocabulary memorization plan with me and supervised me to memorize 100 words every day. On weekends, we would review the words learned before. I used to memorize words on and off. After implementing the plan, my vocabulary test score increased by 100 correct answers after one week.

**Gao Muyang**:

In terms of rules, are there any family agreements regarding time management or mobile phone use? How are these rules implemented? What impact do they have on your learning?

**Student Han**:

When I go home on holidays, my mother will urge me to study and not let me play with my mobile phone all the time. Knowing that I have an exam to take, my parents will supervise me. I will consciously control myself and delete some entertainment apps. This prevents me from being distracted by mobile phone messages while studying.

**Gao Muyang**:

If you hadn't received this support or if the support had been reduced, how do you think the outcome would have been different? Why?

**Student Han**:

If I hadn't received this support, I might not have had so many learning resources and would have found it difficult to stick to my study plan. For example, if my parents hadn't helped me enroll in the English course, my progress in grammar, reading, and translation would not have been so fast. Without their supervision, I might have played with my mobile phone frequently, and my learning efficiency would not have been so high.

**Gao Muyang**:

Have you noticed that differences in family support among classmates or friends have led to gaps in their learning? Please give an example from your身边.

**Student Han**:

I have a classmate. We used to have similar scores, but his family didn't care much about his study and didn't enroll him in any extra - curricular classes. So he was not self - disciplined enough and didn't have his family's care. However, with my family's support, my English score this semester is 20 points higher than his.

**Gao Muyang**:

Please talk about a complete process of your preparation for an exam or learning a certain program. How did you set goals, arrange time, check progress, and review knowledge?

**Student Han**:

When preparing for the final exam or CET - 4 and CET - 6, I would listen to 20 minutes of English news every morning or evening and do reading exercises to check my progress. Then I would identify my weak points through the wrong questions and conduct targeted practice. This also helped me pass CET - 4 and CET - 6 at one time.

**Gao Muyang**:

What are the three most common distractions you encounter while studying? For example, scrolling through your phone, noise, or a backlog of tasks. How do you specifically deal with them?

**Student Han**:

The most common problems I encounter are scrolling through my phone and the hot weather. My solutions are to lock my mobile phone and study in an air - conditioned room.

**Gao Muyang**:

Have you ever had an experience where you had good self - control and achieved significantly better results? What methods did you use at that time, how long did you use them, and what changes did you finally achieve?

**Student Han**:

When preparing for CET - 4 and CET - 6, I controlled the time I spent on my mobile phone every day and spent most of my time doing practice questions. After persisting for that period of time, I achieved an ideal score in the exam.

**Gao Muyang**:

Please also talk about an experience where you had poor self - control. What happened to you at that time? How did you adjust yourself later?

**Student Han**:

Once in class, I received a message notification and scrolled through my mobile phone for a while, which made me fail to keep up with the course content. Later, I adjusted myself by asking my classmates for notes, making up for the missed class, and setting my mobile phone to the do - not - disturb mode during study.

**Gao Muyang**:

When learning English online, when do you clearly feel that it is interesting, engaging, enjoyable, or makes you confident? What situations usually trigger these emotions? For example, anonymous chatting, being able to watch replays, setting your own pace, or receiving feedback from teachers.

**Student Han**:

When practicing oral English on the English app, I will be very happy when I receive praise from the AI foreign teacher. The teacher will also use interesting methods to arouse our interest in class. I think it is very interesting to listen to, and it is also helpful for my study.

**Gao Muyang**:

Talk about an event where positive emotions emerged, what you did as a result, and how your learning outcomes changed in the end.

**Student Han**:

When studying, I would communicate with my friends, which made me very happy. We made progress together, which gave me full motivation. When reviewing for the final exam, we often reviewed and discussed knowledge points together. Finally, we all got high scores.

**Gao Muyang**:

Did you become more willing to participate or speak up because online learning didn't require face - to - face interaction? What did you do then, and what differences did you see in your assignments, tests, or oral English performance?

**Student Han**:

Yes, I am more willing to speak up online. Once after an online course, I left a message on the teacher's official account, and the teacher replied to me. I felt very happy, and my class performance also became much better, so I made rapid progress, and the correct rate of the homework assigned by the teacher also increased.

**Gao Muyang**:

On the contrary, what situations make you feel anxious, bored, or tired? How do you usually adjust yourself to get back to a good learning state?

**Gao Muyang**:

Encountering grammar points that I can't understand, online courses that are too boring, or studying for a long time continuously will make me feel anxious and bored. I will pause my study, scroll through my mobile phone for a few minutes, or walk outdoors for a while, and then continue studying.

**Gao Muyang**:

Please tell a complete chain story for each of the following two scenarios. The first one is about how family support leads to changes in self - control ability and then affects your learning outcomes. The second one is about how family support leads to an improvement in positive emotions and then affects your learning outcomes.

**Student Han**:

First, regarding family support. Before, when I was at home, my parents saw that I was always playing, so they set a study time for me and made rules for mobile phone use. Gradually, I developed the habit of not touching my mobile phone while studying, and the completion rate of my homework also improved. Second, family support enhances my positive emotions. My family often encourages me, which makes me more enthusiastic about learning English. I actively participate in interactions with the teacher in every class, and my oral English expression becomes more and more fluent.

**Gao Muyang**:

Based on your experience, which of these two chains is more important to you? Why?

**Student Han**:

I think the first chain is more important to me. Because the improvement of self - control ability allows me to study continuously, makes me more self - disciplined, and enables me to implement the plan more effectively.

**Gao Muyang**:

Will these two chains affect each other? For example, being more self - disciplined first makes you feel better, or having a better mindset first makes it easier to persist. Please use a specific event to explain.

**Student Han**:

There will be an impact. For example, because I completed my study tasks through self - discipline, I felt better. When I was in a good mood, it was also easier for me to stick to my study plan. Once, because I was in a good mood, I completed the vocabulary memorization task beyond the target. In the following days, my enthusiasm for memorizing words was very high, and my self - control was also better.

**Gao Muyang**:

If we summarize your experience as "family support affects self - control ability and mood, which in turn affect the effectiveness of online English learning", do you think this is in line with your situation?

**Student Han**:

It is in line with my situation. Family support enables me to be more self - disciplined and maintain a more positive mood in my study, which improves my learning effect.

**Gao Muyang**:

Are there any key factors in your experience that we haven't mentioned yet? For example, classmates, teacher feedback, the difficulty of the courses, or how you evaluate these factors. How do they affect your learning?

**Student Han**:

Teacher feedback is very crucial. Once, the teacher corrected my English composition online, pointed out the grammatical errors and the expressions that could be improved. After I revised it according to the teacher's suggestions, my composition level improved.

**Gao Muyang**:

If you were to rank them, what are the three most critical factors for you? And please explain your reasons.

**Student Han**:

The first one is self - control ability, because it allows me to take the initiative to study. The second one is family support, which can provide me with help and motivation when I encounter difficulties. The third one is teacher feedback, which helps me know my shortcomings and make improvements.

**Gao Muyang**:

What online English platforms or functions have you used? For example, self - paced learning, personalized recommendations, live foreign teacher classes, small - group interactive learning, or gamified rewards. Please explain why you chose them.

**Student Han**:

I have used the AI foreign teacher function of "English Fluency" and the picture - text vocabulary memorization function of "Bu Bei Dan Ci" (Don't Memorize Words). I chose "English Fluency" because it can correct my pronunciation, and "Bu Bei Dan Ci" because the combination of pictures and text makes it easier for me to remember words.

**Gao Muyang**:

Choose a function that you use most frequently or that leaves the deepest impression on you. Talk about how it changed your goals, progress, mood, or way of participation, and what learning outcomes it finally brought about.

**Student Han**:

The AI foreign teacher function of "English Fluency" allows me to practice oral English at any time without making an appointment with a real foreign teacher. In this way, I can practice every day and express myself more confidently and freely.

**Gao Muyang**:

Are there any drawbacks to these technologies? For example, distracting your attention, having too much information, or causing anxiety. How do you balance their advantages and disadvantages?

**Student Han**:

There are such situations. Because there is a lot of information on online platforms, it is easy for me to be distracted. So I will make a plan in advance for what I need to do every day.

**Gao Muyang**:

Compared with your classmates around you, what advantages or limitations do you have in terms of equipment, network connection, study location, access to courses or foreign teacher services, and your family's attitudes?

**Student Han**:

My advantages are that I have an independent study space, family support, and a laptop. My limitation is that I don't have a tablet, which is more convenient for taking notes.

**Gao Muyang**:

How do these differences lead to gaps in learning outcomes at critical times, such as during exam preparation, important assignments, or projects? Please give an example.

**Student Han**:

Once, I needed to make an English PPT presentation, which required drawing and typesetting. My laptop was not as convenient as my classmates' tablets for this task. They completed their presentations quickly and beautifully, but I spent a lot of time on mine.

**Gao Muyang**:

Have you observed any cases where learning gaps were made up through methods or support? How was this achieved?

**Student Han**:

I have a classmate who was not good at English translation, but he didn't give up. He read articles and learned translation every day, and went to the library to study every day, making use of the library's network and resources. Finally, he got a high score in the translation exam.

**Gao Muyang**:

Based on your experience, please give one most practical suggestion that students, parents, schools, or platforms can try immediately. Explain what to do, how to do it, and how often to do it.

**Student Han**:

For students, set a specific small goal for English learning every day, such as memorizing 60 words every day, and check the progress once a week. For parents, spend a few minutes every day communicating with their children in English and asking them what English content they have learned that day. For platforms, add more interactive games, such as English vocabulary matching games.

**Gao Muyang**:

If you were to learn English online again next semester, what three things would you change first? Why?

**Student Han**:

First, I will increase the frequency of interaction with foreign teachers. Because I feel that I didn't communicate enough with foreign teachers this semester, and my oral English progress is slow. Second, I will optimize my study time. I will use the time in the evening when my efficiency is low for simple review, and use the time when my efficiency is high for learning key and difficult points. Finally, I will change to a more portable device, such as a tablet, which is convenient for taking notes and studying at any time.

**Gao Muyang**:

Let me briefly repeat the key points you shared today. Please see if it is correct. Today, you mainly talked about the support your family provided in terms of emotion, material, and methods when you learned English online this semester. This made you feel that your self - ability has improved and your emotions have accumulated, thus improving your learning effect.

**Gao Muyang**:

After the interview record is completed, if needed, would you be willing to help check the important content or supplement materials?

**Student Han**:

Yes, if needed, I can help check and supplement the materials.

**Gao Muyang**:

Thank you. Wish you a happy day!

## Interviewee 4: Student Qin

- **Interview Time**: September 21, 2024
- **Interview Method**: Face-to-face interview
- **Interviewer**: Gao Muyang

**Gao Muyang**:

The purpose of this interview is mainly to hear about your feelings and experiences of online English learning recently. The whole process will be anonymous and only used for academic research. You can agree to the recording or request to stop at any time during the interview. Please try to use specific events to explain, such as when it happened, who was involved, what you did, and what the final result was.

**Gao Muyang**:

Could you spend 2 to 3 minutes talking about how you learned English online in the recent semester? Where did you mainly study? What methods did you use? And what is your core goal of learning English?

**Student Qin**:

In the recent semester, I have been learning English online by using the English vocabulary app "Momo Bei Dan Ci" (Momo Memorizes Words) and the online courses on Kuaike Cloud Disk. My core goal of learning English is to get a good score in the postgraduate entrance examination English.

**Gao Muyang**:

What are the most commonly used online English learning platforms, courses, or foreign teacher services for you recently? Why did you choose them?

**Student Qin**:

The most commonly used online English learning platforms for me are "Momo Bei Dan Ci" and the online courses on Kuaike Cloud Disk. I usually watch Teacher Tian Jing's grammar courses and Teacher Tang Chi's reading methodology courses. I chose them because they are free courses and can help me improve my English score.

**Gao Muyang**:

How about your equipment, network connection, and study location? How do these conditions affect your learning?

**Student Qin**:

My equipment includes a tablet and a mobile phone, and I mainly use the tablet for learning. The network connection is very good, and the study location is also very cool with air conditioning, which is the school library. These conditions are all helpful for my learning.

**Gao Muyang**:

Looking back on this semester, have there been any incidents where your family's support significantly improved your learning outcomes? Please tell me about it according to the cause of the incident, what your family did, what you did in response, and the final result.

**Student Qin**:

This semester, my family mainly provided me with a lot of financial support. Without their support, I would not have been able to buy many books and courses. Their support made my learning attitude better, and I could learn more knowledge. Therefore, I became more diligent in my studies, and my academic performance was much better than before.

**Gao Muyang**:

In terms of emotion, how can your family's attitude or companionship make you more motivated or persistent in your studies? When did such situations occur, and what changes did you see in your final grades, assignments, or oral English input?

**Student Qin**:

Emotionally, my family mainly gives me encouragement and support. When I am very tired from studying, they will encourage me not to be too anxious and nervous, and let me relax. Finally, my grades will become better because I am not so nervous.

**Gao Muyang**:

In terms of materials or tools, has your family provided you with equipment, network connection, a dedicated study space, or course funds? Which one has the most direct impact on your learning outcomes? Please give an example.

**Student Qin**:

In this aspect, my family has provided me with equipment and network connection. Since I mainly study in the school library, they have not provided me with a study space at home. They have also provided me with course funds, and their support for my course funds is very strong. If I need money for anything related to study, I can ask them for it. I think the most important thing for me is the course funds, because without money, I would not be able to do many things related to study.

**Gao Muyang**:

In terms of learning methods or plans, has your family made study plans with you, checked your progress, or practiced oral English with you? Tell me a specific example where a change in learning methods led to a change in learning outcomes.

**Student Qin**:

In terms of learning methods or plans, my family has not made any plans with me because they have been working in other places. Regarding the progress check, they only know about my learning progress through the learning results that the teacher sends in the class group. The change in my learning method mainly happened because there was a period of time when I was not interested in learning and did not listen carefully in class. But later, I realized that this was wrong, so I started to listen carefully in class, and finally my academic performance improved from failing to a very good level.

**Gao Muyang**:

In terms of rules, are there any family agreements regarding time management or mobile phone use? How are these rules implemented? What impact do they have on your learning?

**Student Qin**:

In terms of rules, my family has a rule that I cannot use my mobile phone after 11 o'clock at night, and for time management, I have to get up before 8 o'clock in the morning. I strictly implement these rules, which are very helpful for my learning and prevent me from sleeping in class.

**Gao Muyang**:

If you hadn't received this support or if the support had been reduced, how do you think the outcome would have been different? Why?

**Student Qin**:

If my parents had not provided this support or had reduced the support, my learning effect would have been much worse, because many learning conditions would not have been met.

**Gao Muyang**:

Have you noticed that differences in family support among classmates or friends have led to gaps in their learning? Please give an example from your身边.

**Student Qin**:

Yes, I have. For example, I have two classmates. They initially had similar academic performance. One classmate's family is relatively wealthy, and his parents often hire foreign teachers to tutor him, and also provide him with timely tutoring for his weak subjects. The other classmate's parents often work in other places, so no one supervises his study, and he does not have access to more learning resources. As a result, the first classmate's academic performance has been among the top, and finally went abroad to study, while the other classmate's academic performance has become worse and worse.

**Gao Muyang**:

Please talk about a complete process of your preparation for an exam or learning a certain program. How did you set goals, arrange time, check progress, and review knowledge?

**Student Qin**:

In the process of preparing for the postgraduate entrance examination, my goal is to be admitted to a postgraduate program. In terms of time arrangement, I get up at 6 o'clock in the morning and return to the dormitory at 10 o'clock in the evening. Regarding the progress, I follow my own learning progress without too much pressure. For review, after finishing the exercises every night, I conduct targeted practice on the questions I answered incorrectly, and extract the words I don't know to learn separately.

**Gao Muyang**:

What are the three most common distractions you encounter while studying? For example, scrolling through your phone, noise, or a lot of things to do. How do you specifically deal with them?

**Student Qin**:

The three most common distractions I encounter in my study are going to bed late at night, being unable to get up in the morning, and being addicted to scrolling through my phone. To deal with these, after returning to the dormitory at night, I put my mobile phone under the bed after the specified time and then go to bed. In the morning, I strictly get up on time and control the time I spend on my mobile phone. When studying, I put my mobile phone in another place.

**Gao Muyang**:

Have you ever had an experience where you had good self - control and achieved significantly better results? What methods did you use at that time, how long did you use them, and what changes did you finally achieve?

**Student Qin**:

Because I didn't study much at home during the summer vacation, when I returned to school, I put my mobile phone on the desk and went to another place to recite without taking any electronic products. As a result, my efficiency in reciting improved greatly. I used this method for one day, and finally, I no longer wanted to play with my mobile phone when reciting, and my reciting efficiency also improved.

**Gao Muyang**:

Please also talk about an experience where you had poor self - control. What happened to you at that time? How did you adjust yourself later?

**Student Qin**:

Because I have always had poor self - discipline, I can't concentrate on my studies and always want to play or do other things. As a result, my academic performance has plummeted. Others have finished the first round of study, but I haven't even started. Finally, I adjusted myself when I saw that the time left was getting less and less.

**Gao Muyang**:

When learning English online, when do you clearly feel that it is interesting, engaging, enjoyable, or makes you confident? What situations usually trigger these emotions? For example, anonymous chatting, being able to watch replays, setting your own pace, or receiving feedback from teachers.

**Student Qin**:

When learning English online, for example, when I watch Teacher Liu Xiaoyan's vocabulary courses, she uses a very humorous way to help us remember words. For example, she splits a word into different meanings, which makes me feel very happy. After listening to her courses and remembering those words, I will become very confident. These emotions are usually triggered by the teacher's good teaching methods and the ability to set my own learning pace.

**Gao Muyang**:

Talk about an event where positive emotions emerged, what you did as a result, and what changes occurred in your learning outcomes.

**Student Qin**:

When my desire to be admitted to a postgraduate program reached its peak, I would study very hard. I wanted to make up for the progress I had fallen behind. Finally, my learning outcomes became very good, and my progress became faster and faster.

**Gao Muyang**:

Did you become more willing to participate or speak up because online learning didn't require face - to - face interaction? What did you do then, and what differences did you see in your assignments, tests, or oral English performance?

**Student Qin**:

The fact that online learning does not require face - to - face interaction makes me feel more comfortable, and I can learn without facing the teacher directly, which makes me more willing to participate. When I was learning online, I actively responded to the teacher. Later, the correct rate of my homework increased, and my oral English became more fluent.

**Gao Muyang**:

On the contrary, what situations make you feel anxious, bored, or tired? How do you usually adjust yourself to get back to a good learning state?

**Student Qin**:

For example, after listening to a long reading course, I need to do reading exercises. When I find that I have made many mistakes, I will feel very tired and anxious. I usually adjust my mood first and then conduct targeted review of the questions I answered incorrectly. In this way, I can get back to a good learning state.

**Gao Muyang**:

Please tell a complete chain story for each of the following two scenarios. The first one is about how family support leads to changes in self - control ability and then affects your learning outcomes. The second one is about how family support leads to an improvement in positive emotions and then affects your learning outcomes.

**Student Qin**:

Family support is very important to me. First, for example, when I am studying, my family often calls me to encourage me and tell me not to be too anxious. As my self - control ability has improved, my habit of playing with my mobile phone has gradually decreased, and I have become more interested in learning. This has also made my learning effect better. Second, through family support, I can feel the motivation to move forward, so my positive emotions will increase more and more, which will make me more motivated to learn and be very helpful to my learning effect.

**Gao Muyang**:

Based on your experience, which of these two chains is more important to you? Why?

**Student Qin**:

I think the first chain is more important to me. Because not only do I need family support, but self - control ability is also very important. The combination of these two is perfect.

**Gao Muyang**:

Will these two chains affect each other? For example, being more self - disciplined first makes you feel better, or having a better mindset first makes it easier to persist.

**Student Qin**:

These two chains do affect each other. For example, if I am more self - disciplined, I will complete my learning tasks better and finish the learning tasks accordingly. In this way, my mood will become better. When I am in a good mood, I will be more willing to study and not be so anxious, so it will become easier to persist. For example, when doing English reading exercises, I think it is necessary to adjust my mindset first. Don't be unwilling to do the exercises because there are too many of them. Only by adjusting my mindset can I have the motivation to persist.

**Gao Muyang**:

If we summarize your experience as "family support affects self - control ability and mood, which in turn affect the effectiveness of online English learning", do you think this is in line with your situation?

**Student Qin**:

I don't think it is in line with my situation. Because I think learning mainly depends on self - control ability. Family support is not the most important factor, but it can give me motivation.

**Gao Muyang**:

Are there any key factors in your experience that we haven't mentioned yet? For example, classmates, teacher feedback, the difficulty of the courses, or evaluation methods.

**Student Qin**:

I think there are. For example, the difficulty of the courses and teacher feedback have a great impact on me. Because through teacher feedback, I can know my shortcomings. In addition, if the courses are too difficult, I will lose some confidence.

**Gao Muyang**:

If you were to rank them, what are the three most critical factors for you? And please explain your reasons.

**Student Qin**: The first factor is self-control ability, the second is mindset, and the third is time. Only with a good mindset can one have the motivation to learn; stronger self-control improves learning efficiency; and sufficient time provides more room for preparation.

**Interviewer**: What online English platforms or features have you used, such as self-adjustable learning, personalized recommendations, live foreign teacher classes, small-group interactive learning, or gamified rewards?

**Student Qin**: I have used Momo Word (Momo Bei Danci)’s situational word-memorization feature. It makes memorizing words more interesting for me, so I highly recommend it—it also helps deepen my impression of words. Additionally, the app uses the Ebbinghaus Forgetting Curve for daily word learning, which helps me remember words more firmly.

**Interviewer**: Are there any differences in these technologies? For example, do they cause distractions, information overload, or anxiety? Choose the feature you use most frequently or find most memorable, and talk about how it changed your goals, sense of progress, mood, or participation methods, and finally what results it brought.

**Student Qin**: The feature I use most often is Momo Word’s dictation function. After memorizing words, it allows me to review them, helping me identify which words I still struggle with.

**Interviewer**: Are there any downsides to these technologies—like distractions, information overload, or anxiety? How do you balance the pros and cons?

**Student Qin**: Yes, there are downsides. Since they are all electronic apps, I sometimes get distracted while studying and want to scroll through Xiaohongshu or Douyin. However, once my self-control improved, these negative impacts disappeared.

**Interviewer**: Compared with your classmates, what advantages or limitations do you have in terms of equipment, internet access, study space, opportunities for courses or foreign teachers, and family attitudes?

**Student Qin**: I think I have an advantage in family attitudes—my parents don’t put too much pressure on me and fully support my ideas. But I face limitations in opportunities for foreign teachers due to my family’s financial situation.

**Interviewer**: How do these differences lead to gaps in learning outcomes at critical times, such as during exam preparation, important assignments, or projects? Please give an example.

**Student Qin**: The advantages made me more confident and focused when preparing for exams, but I don’t think the limitations caused any gaps.

**Interviewer**: Have you seen examples where gaps were filled through methods or support? How was it done?

**Student Qin**: There was a classmate whose English score was very low—only 30-40 points. But later, through his own efforts and extra tutoring, his score improved significantly and stabilized at 70-80 points.

**Interviewer**: Based on your experience, give one immediate, actionable suggestion each for students, parents, schools, and platforms. Explain what to do, how to do it, and how often.

**Student Qin**:

- For students: Put down your phone, read more books, and exercise more. For example, exercise after studying, three times a week.
- For parents: Don’t put too much pressure on your children; give them free time to study independently.
- For schools: Allocate more time for students’ self-directed learning.
- For platforms: Add more engaging, high-quality teaching content.

**Interviewer**: If you take online English courses again next semester, which three things will you prioritize changing? Why?

**Student Qin**: First, I will research the content of online courses in advance and choose a teacher that suits my learning needs. Second, I will make myself more focused on studying. Third, I will ensure more sufficient study time. Only by doing these can I better improve my grades.

**Interviewer**: Let me briefly recap the key points you shared today. Please tell me if there’s anything to add. You chose online English learning for postgraduate exam preparation; you think family support helped you a lot, but self-control is the most important; you recommend Momo Word as an online English learning platform; and positive emotions definitely had a good impact on your final results. Is there anything to add?

**Student Qin**: No, that’s all.

**Interviewer**: After the interview record is organized, would you be willing to help verify key summaries or supplement materials if needed?

**Student Qin**: I am very willing.

**Interviewer**: Thank you. Wish you a pleasant life!

## Interviewee 2: CZH

**Interview Time**: September 21, 2024**Interview Method**: Face-to-face**Interviewer**: Gao Muyang

**Interviewer**: This interview aims to understand your experiences and feelings about online English learning in the past semester. It is completely anonymous and only for academic research. You can refuse recording or stop the interview at any time. Please try to use specific stories/situations to explain—when it happened, who was involved, what you did, and what the result was.

**Interviewer**: First question: Could you spend 2-3 minutes talking about how you studied English online in the past semester? Where did you mainly study, what methods did you use, and what was your core goal?

**CZH**: In the past semester, I mainly studied English online using a tablet, usually in the library, and I used online courses from cloud storage drives. My core goal for learning English is to prepare for the postgraduate exam.

**Interviewer**: What are your most frequently used English learning platforms, courses, or foreign teacher services? Why did you choose them?

**CZH**: I use Kuake (Quark). It has many courses, such as those for postgraduate exams and TEM-8. I don’t use foreign teacher services. I chose it because it’s cost-effective and the courses are well-taught.

**Interviewer**: How is your equipment, internet access, and study space? How do these conditions affect your learning?

**CZH**: My equipment, internet, and study space are all good. They have a positive impact on my learning, with no negative effects.

**Interviewer**: Looking back at this semester, has there been a time when family support significantly changed your learning outcomes? Please explain it in the order of "cause—what your family did—what you did as a result—outcome".

**CZH**: No, there wasn’t. There were no particularly obvious changes; everything was consistent.

**Interviewer**: Emotionally, how can your family’s attitude or companionship make you more motivated or persistent in learning? When did this happen, and what changes did it bring to your final grades, assignments, or oral English output?

**CZH**: For example, when I study at home, my mom not scolding me is the greatest support she can give. And my grades were pretty good in the end.

**Interviewer**: In terms of materials or tools, has your family provided you with equipment, internet access, a dedicated study space, or course fees? Which one had the most direct impact on your learning outcomes? Please give an example.

**CZH**: My family provided all these things. The one that had the most direct impact on my learning outcomes is probably the courses. For example, my family provided money to buy online courses, and I absorbed what was taught in the courses, which helped my studies a lot. However, during the pandemic, when I lived in my hometown, there was no desk or computer at home—I could only take online courses on my phone in the bedroom, which affected my learning effectiveness.

**Interviewer**: In terms of learning methods or plans, has your family worked with you to make plans, check your progress, or practice oral English with you? Tell a story where a change in learning method led to a change in learning outcomes.

**CZH**: No, I’ve always studied on my own. My family only provides financial support and emotional support.

**Interviewer**: Regarding rules, does your family have agreements on time management or phone use? How are these rules implemented, and what impact do they have on your learning?

**CZH**: The time management rule is that I must go to bed before 11 PM, and for phone use, the rule is to use it as little as possible. How are these rules implemented? My family reminds me of them anytime, anywhere. They don’t have much impact on my learning; the biggest impact is that I get enough sleep.

**Interviewer**: If you didn’t have this support, or if the support was reduced, how do you think the outcome would be different? Why?

**CZH**: The outcome would definitely be different. Without my family’s support, I couldn’t do anything. I’m not financially independent at this stage.

**Interviewer**: Have you noticed gaps in learning between classmates or friends due to differences in family support? Please give an example.

**CZH**: I don’t think there are such examples around me.

**Interviewer**: Tell a complete process of your exam preparation or learning for a project. How did you set goals, arrange time, check progress, and review knowledge?

**CZH**: Setting goals—I don’t usually set goals, but sometimes I get inspired and list what I need to do the next day the night before. For time arrangement, I don’t have a clear schedule either; I just decide what to do in the morning, afternoon, and evening. For progress checks, I don’t really do that. For review, I might review once a week, but that’s rare.

**Interviewer**: What are the three most common distractions you encounter while studying—like scrolling through your phone, noise, or piled-up tasks? How do you specifically deal with them?

**CZH**: First, scrolling through my phone is definitely a distraction. My solution is to delete all video apps (like Douyin) and TV-watching apps from my phone. Second, I used to have a lot of tasks when I had classes; my solution was to study more after class. Third, I can be lazy—for example, I can’t focus when studying at home. My solution is to come to school to study.

**Interviewer**: Also, please share an experience where good self-control led to significantly better results. What method did you use, how long did you use it, and what changes did it finally bring?

**CZH**: It was probably when I first came back to school from home. The days when I studied well during that period can be considered a time of good self-control, and the results were obviously better. At home, I might study 3-4 hours a day, but at school, I studied more than 10 hours a day. The method was just sitting in the library.

**Interviewer**: Also, share an experience where your self-control failed. What happened then? How did you adjust and get back on track later?

**CZH**: The bad experience was when I went home; later, I came back to school, and that’s how I adjusted.

**Interviewer**: During online English learning, when do you clearly feel interested, engaged, happy, or confident? What situations usually trigger these emotions—such as anonymous chat, being able to watch replays, setting your own pace, or getting feedback from teachers?

**CZH**: The most interesting part of online English learning for me is when the teacher explains a method in class, and I apply it to my studies—like solving problems. If I successfully use the method and see obvious results, I find it interesting, become more engaged, feel happy, and gain confidence.

**Interviewer**: Tell a story where positive emotions emerged, what actions you took because of them, and finally how your learning outcomes changed.

**CZH**: The emergence of positive emotions probably came when I was in my senior year and needed to prepare for the postgraduate exam. It was a shift from a very negative state to a positive one for me. What did I do? I made many plans and researched a lot of information. Since these were things I wanted to do myself, my final learning outcomes were pretty good.

**Interviewer**: Did the fact that online learning doesn’t require in-person interaction make you more willing to participate or speak up? What did you do, and how did it change your assignments, quizzes, or oral English performance?

**CZH**: I think it definitely did a little. Without face-to-face interaction, I feel less awkward. What I did was have a conversation with the teacher, and I feel my oral English improved a bit.

**Interviewer**: On the contrary, what situations make you feel anxious, bored, or tired? How do you usually adjust to get back to effective learning?

**CZH**: I feel anxious when I’m playing, or when I have a tight schedule but feel like I have nothing to do—even though there are still many things left undone. My adjustment method is to tell myself to study for another hour, no matter what I study. Then I can usually get back to good learning habits.

**Interviewer**: Please tell a complete "chain story" for each of the following: 1. Family support leads to changes in self-control, which affects learning outcomes; 2. Family support leads to improved positive emotions, which affects learning outcomes.

**CZH**:

For family support—take my postgraduate exam preparation as an example. If my family didn’t support me, I wouldn’t have the money. For instance, I wanted to buy a tablet for studying. My mom was worried I would just play with it and refused to buy it, but my dad said, “I believe in you; you can definitely control yourself.” So my dad bought it for me. To live up to his trust, my self-control improved in this aspect, and the final outcome was better.

Also, because I’m preparing for the postgraduate exam, my family is very supportive. They call me from time to time to ask how I’m doing and tell me not to be nervous or anxious, saying the final result isn’t the most important thing. These words helped relieve my anxiety. As for learning outcomes, we’ll have to wait until after the exam to see, but my phased results are pretty good.

**Interviewer**: Based on your experience, which chain is more important to you?

**CZH**: The second chain is definitely more important. Because I think self-control doesn’t have much to do with the support my family gives me; what really matters is the emotional support they provide—it makes me want to do something and put more effort into doing it well.

**Interviewer**: Do these two chains influence each other? For example, does being more self-disciplined first make you feel better, or does having a better mindset first make it easier to persist? Use a specific story to explain.

**CZH**: They definitely influence each other. A better mindset makes it easier to persist. It’s easy to feel anxious while studying—even if I have self-control, I need a good mindset first to keep going. If I’m studying but can’t calm down, I won’t learn well. But if my mindset is good, I can keep studying effectively.

**Interviewer**: If we summarize your experience as “family support affects self-control and mood, which in turn affects the effectiveness of online English learning”, do you think this fits your situation?

**CZH**: I don’t think it fits.

**Interviewer**: Are there any key factors in your experience that we haven’t mentioned yet—such as classmates, teacher feedback, course difficulty, or evaluation methods? How do they affect your learning?

**CZH**: I think a key factor is the teacher’s teaching style. I’m the kind of person who won’t want to listen if a class is very dull and has no interesting parts. But the online courses I’m taking now have teachers who make classes interesting—they use fun content related to the lessons to engage us. So I think this is crucial.

**Interviewer**: If you had to rank them, what are the three most critical factors for you? Explain your reasons.

**CZH**: The three most critical factors are, first, my own mindset; second, family support; and third, self-control. Mindset is important because the long and boring learning process can feel difficult. Second, family support gives me more motivation. Finally, self-control allows me to get rid of distractions and focus on studying.

**Interviewer**: What online English platforms or features have you used—such as self-adjustable learning, personalized recommendations, live foreign teacher classes, small-group interactive learning, or gamified rewards? Explain why you chose them.

**CZH**: There are quite a few online English learning platforms, like Keke English (Coco English). One thing I like about it is that when I used it to memorize words, it had a “level-breaking” mode instead of letting me decide how many words to memorize each day. Sometimes I found it quite interesting. There’s also a dubbing app that’s fun—it helps improve oral English.

**Interviewer**: Choose the feature you use most often or find most memorable. Talk about how it changed your goals, progress, mood, or participation methods, and finally what learning results it brought.

**CZH**: The one I use most often is English Fun Dubbing. Since it’s a dubbing app, it scores my dubbing after I finish. It lets me know where I have problems, so I can adjust accordingly. My progress speed has always been slow, so it didn’t change that much. For mood—I feel happy when I get a high score, but I don’t feel bad when I get a low score because my oral English has always been poor. The final learning result is that my oral English improved a little.

**Interviewer**: Are there any downsides to these technologies—like distractions, information overload, or anxiety? How do you balance the pros and cons?

**CZH**: I think a major downside is that they can distract me. Since they’re phone apps, I balance this by setting a daily time limit for using them—I set a timer on my phone, and it shuts down the app when the time is up. So the impact is relatively reduced.

**Interviewer**: Compared with your classmates, what advantages or limitations do you have in terms of equipment, internet access, study space, opportunities for courses or foreign teachers, and family attitudes?

**CZH**: I don’t have limitations in equipment, internet access, or study space. But I have few opportunities for foreign teachers due to family reasons. My main study space is at home—I’m not sure, but this probably relates to my self-control. I have a dedicated study space, but since it’s at home, I can’t focus. I think other aspects are similar among everyone.

**Interviewer**: How do these differences lead to gaps in learning outcomes at critical times, such as during exam preparation, important assignments, or projects? Please give an example.

**CZH**: For example, if I study at home, I’ll definitely be much worse than others. My learning outcomes will be very poor because I can’t focus at home.

**Interviewer**: Have you seen examples where gaps were filled through methods or support? How was it done?

**CZH**: Taking the example I mentioned earlier—by coming to school, the gap can be filled. For foreign teacher opportunities, my parents would need to earn more money for me to have that chance.

**Interviewer**: Based on your experience, give one immediate, actionable suggestion each for students, parents, schools, and platforms. Explain what to do, how to do it, and how often.

**CZH**:

- For students: Spend 10 minutes each day doing English morning reading, using a good online app. Following the app’s readings helps with more standard pronunciation and better language sense.
- For parents: Help your children develop English skills from an early age—for example, using English cartoons.
- For platforms: Develop daily English task templates, such as word spelling and short sentence translation, and give rewards to students after they complete the tasks.
- For schools: Provide more opportunities for students to communicate and learn from each other.

**Interviewer**: If you take online English courses again next semester, which three things will you prioritize changing? Why?

**CZH**:

Familiarize myself with all features of the online English learning platform in advance to avoid fumbling during class and missing key points from the teacher (I’ve had this problem before).

Make a more detailed online study plan, clarifying the learning goals and post-class tasks for each session (I previously lacked planning and had low efficiency).

Find online classmates to supervise each other’s learning and discuss problems together (I used to study alone, couldn’t solve problems in time, and easily slacked off).

**Interviewer**: Let me briefly recap the key points you shared today. Please tell me if it’s accurate and if there’s anything missing. You said you make better progress when studying in the library or at school, but your efficiency drops at home. Family support is important, as is self-control, and positive emotions promote your learning.

**CZH**: Yes, that’s correct.

**Interviewer**: After the interview record is organized, would you be willing to help verify key summaries or supplement materials if needed?

**CZH**: I am very willing.

**Interviewer**: Thank you. Wish you a happy day!

## Interviewee 3: Zhang Ke

**Interview Time**: September 11, 2024**Interview Location**: Library**Interviewer Name**: Zhang Ke**Age**: 20

### Interview Guidelines

**Interviewer**: 1. This interview aims to understand your experiences and feelings about online English learning in the past semester. It is completely anonymous and only for academic research. You may agree to recording and may stop the interview at any time.2. Please try to use specific stories/situations to explain—when it happened, who was involved, what you did, and what the result was.

**Interviewer**: Please use 2-3 minutes to summarize your online English learning experience in the past semester: where you mainly studied, what methods you used, and what your core goal was.

**Zhang Ke**: My recent online English learning experience mainly involves using the “Bubei Danci” (Don’t Memorize Words) app on a tablet to learn vocabulary. For reading, I learned reading methods from Teacher Chai Rong’s courses on Bilibili. My core goal is to achieve a satisfactory score in the 2026 postgraduate English exam—preferably above 50 points.

**Interviewer**: What are your most frequently used platforms, courses, or foreign teacher services? Why did you choose them?

**Zhang Ke**: My most frequently used platform is Bilibili, because most of its resources are free. My most frequently used course is Teacher Chai Rong’s reading course. I find his courses very easy to understand, and the methods are very useful for solving problems.

**Interviewer**: What is your equipment, internet access, and study space like? How do these conditions affect your learning experience?

**Zhang Ke**: I think equipment is quite important for learning. Since I need to prepare for the postgraduate exam, I bought a tablet specifically for studying. A good tablet allows me to engage more deeply in learning—the screen is large and the font is clear.

**Interviewer**: Looking back at this semester, has there been a time when family support significantly changed your learning outcomes? Please explain it in the order of "cause—what your family did—what you did as a result—outcome".

**Zhang Ke**: My family support mainly comes from my grandmother. She promised to give me a financial reward if I get into graduate school. Because of her support, I study harder. I think my current learning results are pretty good.

**Interviewer**: Emotionally, how does your family’s attitude or companionship make you more motivated or persistent in learning? When did this happen, and what did it bring to your final grades, assignments, or oral English output?

**Zhang Ke**: Emotionally, my family fully supports my postgraduate exam preparation. They think academic qualifications are important and hope I can further my studies. When I told them about my plan in July, they agreed that I should improve my academic background. At that time, my English score—since I was memorizing words every day and had just started practicing reading—my reading results were not good. For oral English, I plan to practice it after the preliminary postgraduate exam results come out, so my oral English is not good either at the moment.

**Interviewer**: In terms of materials or tools, has your family provided you with equipment, internet access, a dedicated study space, or course fees? Which one had the most direct impact on your learning output? Please give an example.

**Zhang Ke**: My family bought me a tablet and subsidized the cost of the study materials and online courses I purchased. I think the tablet had the most direct impact on my learning output.

**Interviewer**: In terms of academics or strategies, has your family worked with you to make plans, check your progress, or practice oral English with you? Tell a specific experience where a change in method led to a change in outcome.

**Zhang Ke**: They haven’t helped me make plans yet. They mainly let me make my own plans because they are farmers and don’t have much knowledge in this area.

**Interviewer**: Regarding rules, does your family have agreements on time management or phone use? How are these rules implemented, and what impact do they have on your learning outcomes?

**Zhang Ke**: My family hasn’t set rules for my time management or phone use—I set these for myself. I’ve made time management settings on my phone, and I think this has a great positive impact on my learning.

**Interviewer**: If you didn’t have this support, or if the support was reduced, how do you think the outcome would be different? Why?

**Zhang Ke**: I think the results would be less obvious, because I believe certain constraints are needed to push people to make more progress.

**Interviewer**: Have you noticed gaps in learning between classmates due to differences in family support? Can you give a comparative example?

**Zhang Ke**: Some families provide tablets for their children, while others only let their children use phones to study. I don’t think the gap is big, because a phone is also a learning tool—only with a smaller screen. It mainly depends on the individual. Also, some students choose to attend cram schools for postgraduate exams, while others study independently. I think there is a gap in this aspect.

**Interviewer**: Please describe a complete cycle of your exam preparation or project learning: how you set goals, arrange time, monitor progress, and review.

**Zhang Ke**: For goal-setting, my target scores are: above 230-240 for professional courses, above 50 for English, and 60 for politics. For time arrangement, during exam preparation, I go to the library at 8:30 AM every day, start studying, and leave after 9:30 PM. I eat lunch and dinner near the library entrance, and take a 20-minute nap on my desk. For progress monitoring and review, I rarely do formal progress checks, but I review the content from the previous day on the next day.

**Interviewer**: What are the three most common distractions you encounter while studying—such as social media, noise, or piled-up tasks? What specific strategies do you use to deal with them?

**Zhang Ke**: The most common distractions are:

My phone—it distracts me, and I often pick it up when listening to less important classes. So I give my phone to a friend to manage.

Noise—my major requires memorizing 2-3 textbooks, and my classmates’ recitation in the same area distracts me. So I find a suitable new place to avoid studying in the same area as them.

Piled-up tasks—I prioritize and break them down to reduce pressure.

**Interviewer**: Have you ever had an experience where good self-regulation led to a significant improvement in results? What strategy did you use, how long did you use it, and what changes did it finally bring?

**Zhang Ke**: The most obvious improvement from self-regulation was in the past two days. I found many good reading skills to learn, which greatly improved my reading skills.

**Interviewer**: Also, share an experience where your self-regulation failed. What happened then? How did you adjust and get back on track later?

**Zhang Ke**: After I finished learning all the postgraduate English words, I started doing reading exercises without learning any reading skills first. At that time, my error rate was extremely high. Later, I got back on track by watching skill courses on reading.

**Interviewer**: During online learning, when do you clearly feel interested, engaged, happy, or confident? What situations usually trigger these emotions—such as anonymous/semi-anonymous interaction, watching replays, setting your own pace, or getting feedback from teachers?

**Zhang Ke**: During online learning, I feel more interested in English reading skills. This interest comes from my desire to improve my problem-solving accuracy, and it is mainly triggered by watching replays.

**Interviewer**: Tell a story where positive emotions emerged, what actions you took because of them, and finally how your learning output changed.

**Zhang Ke**: The emergence of positive emotions was also based on the improvement in my reading skill accuracy. Because of this, I did many reading exercises to confirm if I had reached that level. Finally, my learning outcome improved—my accuracy really increased.

**Interviewer**: Did the "physical absence" of the online environment make you more willing to participate or speak up? What did you do, and how did it change your assignments, quizzes, or oral English performance?

**Zhang Ke**: For physical absence in the online environment—like live classes, I usually watch the replay after the class ends. I’m an introvert, so I don’t like to express my opinions online.

**Interviewer**: On the contrary, what situations make you feel anxious, bored, or tired? How do you usually adjust to get back to effective learning?

**Zhang Ke**: I feel anxious when I see my classmates studying while I’m resting or scrolling through my phone. At that time, I put down my phone and start doing exercises or reciting to get back on track.

**Interviewer**: Please tell a complete "chain story" for each of the following: 1. Family support leads to changes in self-regulation, which affects learning outcomes; 2. Family support leads to improved positive emotions, which affects learning outcomes.

**Zhang Ke**:

The material support from my family allowed me to buy reading skill courses, which improved my accuracy. Their emotional support made me feel supported in everything I do, so my motivation became stronger. This led to learning outcomes that are proportional to my efforts.

Thanks to my family’s emotional and material support, I became more strict with my own behavior. This led to my learning outcomes gradually improving.

**Interviewer**: Based on your experience, which chain is more important to you? Why?

**Zhang Ke**: I think it’s the second one, because positive emotions bring me more value. Emotional support is more important than material support for me.

**Interviewer**: Do these two chains influence each other? For example, does being more self-disciplined first make you feel better, or does having a better mindset first make it easier to persist? Use a specific story to explain.

**Zhang Ke**: I don’t think they influence each other. First, preparing for the postgraduate exam or learning English is a long process. You need to be self-disciplined first. If you feel frustrated or sad while solving problems due to a high error rate, you can adjust your mindset to learn better. I think that will lead to better results.

**Interviewer**: If we summarize your experience as “family support affects self-regulation and positive emotions, which in turn affects online learning outcomes”, how well do you think this framework fits your situation?

**Zhang Ke**: I think the fit is very high, because family support is a very important part of my learning. Only with family support can I supervise myself to study.

**Interviewer**: Are there any key factors in your experience that we haven’t mentioned yet—such as classmates, teacher feedback, course difficulty, or evaluation methods? How do they fit into your learning process?

**Zhang Ke**: I think a key factor is peers. Classmates communicate with each other and share what they think are better learning methods. For example, a friend recommended a mini-program for politics—it has questions from various teachers, and I think it really helps me make progress.

**Interviewer**: If you had to rank them, what are the three most critical factors for you? Explain your reasons.

**Zhang Ke**:

Peers: Competitive peers motivate me to study harder.

Course difficulty: If a course is too difficult, it may temporarily reduce my interest in learning, but after a few minutes, my inner perseverance pushes me to understand and apply that knowledge.

Environment: The environment has a great impact on me. A noisy environment greatly reduces my learning efficiency.

**Interviewer**: What online English platforms or features have you used—such as adaptive learning, personalized recommendations, live foreign teacher classes, small-group interactive learning, or gamified incentives? Explain why you chose them.**Zhang Ke**: I have used adaptive learning—I find online courses on my own, buy exam question sets, and study independently. I chose this because, unlike small-group learning, it doesn’t have external pressure to push me forward. I think learning step by step at my own pace leads to better results.

**Interviewer**: Choose the feature you use most often or find most memorable. Talk about how it changed your goal-setting, progress monitoring, emotional experience, or participation methods, and finally what learning results it brought.

**Zhang Ke**: The one I use most often is adaptive learning. It allows me to set my own goals—what I want to achieve today and tomorrow. For progress monitoring, I use a small notebook to write down my daily goals. I think it has brought me good learning results, and I highly recommend this type of adaptive learning.

**Interviewer**: Do these technologies have any side effects—such as interrupted attention, information overload, or anxiety? How do you balance the pros and cons?

**Zhang Ke**: For adaptive learning, I usually watch courses on Bilibili, which has bullet comments. When I see bullet comments saying others got 4 or 5 correct answers out of 5 for a reading passage, I feel very anxious. I think, “Is this question really that easy? Why did I struggle with it?” My solution is to turn off the bullet comments.

**Interviewer**: Compared with your classmates, what advantages or limitations do you have in terms of equipment, internet access, study space, opportunities for courses or foreign teachers, and family attitudes?

**Zhang Ke**: First, I think my equipment is quite good. For internet access, I actively look for free courses. In terms of study space, some classmates attend off-campus cram schools, but my family fully supports me in improving my academic background, so I have sufficient emotional and spiritual support.

**Interviewer**: How do these differences lead to gaps in learning outcomes at critical times, such as during exam preparation, important assignments, or projects? Please give an example.

**Zhang Ke**: I think online learning is very important for my assignments. For example, regarding study space—since I’m a normal education major, I needed to take the Teacher Qualification Exam. If I passed it earlier, I wouldn’t have to distract myself during postgraduate exam preparation.

**Interviewer**: Have you seen examples where gaps were filled through strategies or support? How was it done?

**Zhang Ke**: For example, my roommate is preparing for both the postgraduate exam and the Teacher Qualification Exam. This forces her to split her attention to prepare for the Teacher Qualification Exam. Although the content of the Teacher Qualification Exam is partially included in the postgraduate exam, I think it still has a certain impact.

**Interviewer**: Based on your experience, give one immediate, actionable suggestion each for students, parents, schools, and platforms. Explain what to do, how to do it, and how often.

**Zhang Ke**:

- For students: First, be self-disciplined. Start with the basics—memorize words first, then progress gradually.
- For parents: Provide students with more emotional support and material support.
- For schools: Offer more English-related lectures, such as how to learn English effectively.

**Interviewer**: If you take online courses again next semester, which three things will you prioritize changing? Why?

**Zhang Ke**:

Change my mindset: Previously, I didn’t think English was important, but after preparing for the postgraduate exam, I realize it’s crucial.

Strengthen my action: Action is needed to make gradual progress.

Find an empty classroom to study alone: This helps me focus better.

**Interviewer**: I will briefly recap the key points you shared today. Please confirm if it’s accurate and if there’s anything missing to add.

**Zhang Ke**: Okay.

**Interviewer**: After the interview record is organized, would you be willing to help verify key summaries or supplement materials if needed?

**Zhang Ke**: Yes.

（Note: Encourage interviewees to describe specific situations and timelines; when necessary, use the "cause-action-outcome" structure to follow up.）

## Interviewee 4: Jiang Zhiwei

**Interview Topic**: Interview on Factors Influencing College Students' Online English Learning Outcomes**Interview Time**: September 11, 2024**Interview Location**: Library**Interviewer Name**: Jiang Zhiwei**Age**: 21

### Interview Guidelines

**Interviewer**: 1. This interview aims to understand your experiences and feelings about online English learning in the past semester. It is completely anonymous and only for academic research. You may agree to recording and may stop the interview at any time.2. Please try to use specific stories/situations to explain—when it happened, who was involved, what you did, and what the result was.

**Interviewer**: Please use 2-3 minutes to summarize your online English learning experience in the past semester: where you mainly studied, what methods you used, and what your core goal was.

**Jiang Zhiwei**: Last semester, I mainly prepared for the postgraduate English exam at home through online platforms. My learning methods included attending live online courses (such as those from New Oriental and Kaocong), using apps like Momo Word (Momo Bei Danci) and Shanbei Reading for independent vocabulary and reading training, and regularly doing full sets of past exam papers. My core goal was to score above 65 in Postgraduate English II.

**Interviewer**: What are your most frequently used platforms, courses, or foreign teacher services? Why did you choose them?

**Jiang Zhiwei**: I use New Oriental’s postgraduate English live courses and Momo Word most often. I chose New Oriental’s courses because Teacher Qi explains the problem-solving skills for past exams very thoroughly. I chose Momo Word because its Ebbinghaus Forgetting Curve review strategy is very scientific and helps me memorize a large number of postgraduate exam vocabulary efficiently.

**Interviewer**: What is your equipment, internet access, and study space like? How do these conditions affect your learning experience?

**Jiang Zhiwei**: I have a laptop and an iPad. The internet at home is stable, and I have a dedicated study space. These conditions ensure that I can follow the live courses without interruptions and immerse myself in doing mock exams with past papers—this is crucial for developing the ability to focus for long periods, which is needed for exams.

**Interviewer**: Looking back at this semester, has there been a time when family support significantly changed your learning outcomes? Please explain it in the order of "cause—what your family did—what you did as a result—outcome".

**Jiang Zhiwei**: I struggled with unstable accuracy in reading, especially in analyzing long and complex sentences. My aunt is an English teacher, and she spent half an hour every Saturday morning helping me analyze the long and complex sentences I got wrong that week via video call, explaining the grammatical structure. As a result, I not only understood the wrong questions but also learned how to analyze long and complex sentences on my own, and I organized a mistake notebook. Two months later, my reading accuracy stabilized at over 80%, up from 60% before.

**Interviewer**: Emotionally, how does your family’s attitude or companionship make you more motivated or persistent in learning? When did this happen, and what did it bring to your final grades, assignments, or oral English output?

**Jiang Zhiwei**: Well, every time my mock exam results are not ideal, my mom never criticizes me—she always says, “Take it slow; identifying problems is progress.” This attitude helps me calm down from anxiety and analyze the exam papers more rationally. In the most recent mock exam, my accuracy in cloze tests improved by 15% because I was in a more stable mindset.

**Interviewer**: In terms of materials or tools, has your family provided you with equipment, internet access, a dedicated study space, or course fees? Which one had the most direct impact on your learning output? Please give an example.

**Jiang Zhiwei**: My family upgraded the broadband for me and paid the tuition for New Oriental’s online courses. The paid courses had the most direct impact—they provided a systematic teaching system, high-quality explanations of past exams, and Q&A services, which gave me a clearer direction for review and improved my efficiency.

**Interviewer**: In terms of academics or strategies, has your family worked with you to make plans, check your progress, or practice oral English with you? Tell a specific experience where a change in method led to a change in outcome.

**Jiang Zhiwei**: My mom and I used a calendar to make an 80-day sprint plan for postgraduate English. Every Sunday, she checks the number of days I’ve clocked in for word memorization and the number of past papers I’ve completed. This external supervision kept me from slacking off, and the plan execution rate was very high. As a result, my vocabulary expanded noticeably, and my ability to solve questions (speed and feel) improved.

**Interviewer**: Regarding rules, does your family have agreements on time management or phone use? How are these rules implemented, and what impact do they have on your learning outcomes?

**Jiang Zhiwei**: We agreed that during the 3-5 PM window when I do English mock exams every day, the whole family keeps quiet, and I put my phone in the living room. This rule is strictly enforced—it greatly improved my focus and the authenticity of the mock exams, which naturally boosted my question-solving accuracy.

**Interviewer**: If you didn’t have this support, or if the support was reduced, how do you think the outcome would be different? Why?

**Jiang Zhiwei**: I would probably get distracted more easily like checking my phone while solving questions. The bottleneck with long and complex sentences might never be broken. My score might stay around 55, making it hard to reach 65.

**Interviewer**: Have you noticed gaps in learning between classmates due to differences in family support? Can you give a comparative example ?

**Jiang Zhiwei**: For example, I have two classmates. Classmate A’s family has average financial conditions, so he can only find free resources on his own. His study materials are disorganized, and he lacks systematic guidance—his scores improved slowly. Classmate B’s family not only enrolled him in an expensive full-course cram school but also prepared a dedicated study room and the best equipment for him. He has a good study environment, makes fast progress, and his scores are very stable. This is a clear gap.

**Interviewer**: Please describe a complete cycle of your exam preparation or project learning: how you set goals, arrange time, monitor progress, and review.

**Jiang Zhiwei**: My goal was to carefully read the postgraduate English reading passages from 2010 to 2015 within 10 days. I arranged to carefully read 2 passages every day, analyze the long and complex sentences, and memorize new words. I used an Excel spreadsheet to track my daily completion and the number of unknown words. For review, I summarized which question types had a high error rate on weekends and focused on watching the corresponding sections of the online courses. Finally, I gained a deeper understanding of the question-setting style and common traps in postgraduate English reading.

**Interviewer**: What are the three most common distractions you encounter while studying—such as social media, noise, or piled-up tasks? What specific strategies do you use to deal with them?

**Jiang Zhiwei**: The distractions are:

WeChat group messages and notifications from entertainment apps—I lock my phone so I can’t open it for a short period.

Family noise—I wear noise-canceling headphones.

Unclear communication about my study time with family—I clearly tell my family my study schedule.

**Interviewer**: Have you ever had an experience where good self-regulation led to a significant improvement in results? What strategy did you use, how long did you use it, and what changes did it finally bring?

**Jiang Zhiwei**: The strategy I used was to strictly follow a plan of memorizing words from 6:30 to 8 AM every day without fail, and I used Momo Word’s statistical function to monitor myself. I persisted for half a semester. Finally, my vocabulary increased from about 4,000 to over 6,500, and I almost no longer encountered unknown words that hindered my reading.

**Interviewer**: Also, share an experience where your self-regulation failed. What happened then? How did you adjust and get back on track later?**Jiang Zhiwei**: During the May Day holiday, I relaxed completely and didn’t touch English for a week. When I got back to studying, I found I had lost my “feel” for the questions and got many reading questions wrong. I quickly did a set of familiar past papers to regain my confidence, refined my weekly plan, and asked my roommate to supervise me in executing it.

**Interviewer**: During online learning, when do you clearly feel interested, engaged, happy, or confident? What situations usually trigger these emotions—such as anonymous/semi-anonymous interaction, watching replays, setting your own pace, or getting feedback from teachers?

**Jiang Zhiwei**: I feel very confident and happy when I ask a good question in the anonymous bullet comments of a live course and get public praise from the teacher. Or when I finish reading a difficult article from The Economist on Shanbei Reading, I feel a great sense of accomplishment.

**Interviewer**: Tell a story where positive emotions emerged, what actions you took because of them, and finally how your learning output changed.

**Jiang Zhiwei**: In one mock exam, I got a high score on my essay, and the teacher commented, “Clear structure, authentic language.” This made me very confident, and I developed a strong interest in writing—from fearing writing to proactively writing one extra essay every week and comparing it with model essays online to learn. As a result, my essay went from being a weak point to a stable score booster.

**Interviewer**: Did the "physical absence" of the online environment make you more willing to participate or speak up? What did you do, and how did it change your assignments, quizzes, or oral English performance?

**Jiang Zhiwei**: Yes. In live courses with thousands of participants, I’m more willing to ask questions and answer the teacher’s questions in the anonymous bullet comments. This made me more engaged and helped me understand many questions I previously couldn’t figure out.

**Interviewer**: On the contrary, what situations make you feel anxious, bored, or tired? How do you usually adjust to get back to effective learning?

**Jiang Zhiwei**: I feel very anxious and tired when I keep solving questions but my accuracy doesn’t improve—even drops. At this point, I temporarily put down the past papers and switch to a different learning method, such as watching an English short video or listening to English songs to refresh my mind. I go back to studying after half an hour.

**Interviewer**: Please tell a complete "chain story" for each of the following: 1. Family support leads to changes in self-regulation, which affects learning outcomes; 2. Family support leads to improved positive emotions, which affects learning outcomes.

**Jiang Zhiwei**:

My aunt tutored me in long and complex sentences, which helped me learn how to understand them and organize a mistake notebook. Finally, my reading accuracy improved significantly.

My mom comforted me emotionally, helping me overcome anxiety and stabilize my mindset. As a result, my accuracy in cloze tests improved.

**Interviewer**: Based on your experience, which chain is more important to you? Why?

**Jiang Zhiwei**: I think the first chain (self-regulation) is more important for my learning. Because preparing for the postgraduate exam is a long battle, and in the end, you have to rely on yourself to master learning methods and rules.

**Interviewer**: Do these two chains influence each other? Use a specific story to explain.

**Jiang Zhiwei**: I think they do. First, I learned methods through my aunt’s tutoring, which led to improved grades and a better mood. This in turn gave me the motivation to stick to the original learning method. For example, after my aunt helped me analyze long and complex sentences, I successfully analyzed one on my own when solving a later question—I felt a great sense of accomplishment, which made me more willing to tackle even more difficult sentences.

**Interviewer**: If we summarize your experience as “family support affects self-regulation and positive emotions, which in turn affects online learning outcomes”, how well do you think this framework fits your situation?

**Jiang Zhiwei**: I think it fits my experience perfectly—my experience completely aligns with this chain.

**Interviewer**: Are there any key factors in your experience that we haven’t mentioned yet—such as classmates, teacher feedback, course difficulty, or evaluation methods? How do they fit into your learning process?

**Jiang Zhiwei**: Yes—my postgraduate exam study partners. My online study partners and I clock in for word memorization in a group chat every day to supervise each other. This peer pressure keeps me from slacking off.

**Interviewer**: If you had to rank them, what are the three most critical factors for you? Explain your reasons.

**Jiang Zhiwei**:

Self-regulation: The success of postgraduate exam preparation ultimately depends on oneself.

Family support: It provides me with material foundation and emotional support.

Quality of course platforms: Good guidance makes review more effective with less effort.

**Interviewer**: What online English platforms or features have you used? Explain why you chose them.

**Jiang Zhiwei**: I chose New Oriental Online’s live courses and Q&A services (because they offer online Q&A) and Momo Word (because it intelligently plans reviews, has a database of past exam questions, can generate intelligent reports).

**Interviewer**: Choose the feature you use most often or find most memorable. Talk about how it changed your goal-setting, progress monitoring, emotional experience, or participation methods, and finally what learning results it brought.

**Jiang Zhiwei**: Momo Word’s Ebbinghaus Forgetting Curve review feature. It changed my vocabulary memorization method from blind rote memorization to scientific review. Finally, it almost eliminated vocabulary obstacles in my reading.

**Interviewer**: Do these technologies have any side effects—such as interrupted attention, information overload, or anxiety? How do you balance the pros and cons?

**Jiang Zhiwei**: Sometimes, notification alerts from various apps interrupt my learning. Later, I set all my study apps to “study mode”—turning off notifications—and only handle messages at fixed times.

**Interviewer**: Compared with your classmates, what advantages or limitations do you have in terms of equipment, internet access, study space, opportunities for courses or foreign teachers, and family attitudes?

**Jiang Zhiwei**: My advantages are a stable study space, smooth internet access, and the ability to afford paid courses. My limitation is that compared with students in first-tier cities, I have fewer opportunities to attend high-end offline Q&A sessions or intensive in-person training camps.

**Interviewer**: How do these differences lead to gaps in learning outcomes at critical times, such as during exam preparation, important assignments, or projects? Please give an example.

**Jiang Zhiwei**: During the sprint phase, some classmates attended offline closed-door prediction camps and got more accurate essay prediction materials. I could only rely on online information, so there was a gap in the timeliness and pertinence of the information.

**Interviewer**: Have you seen examples where gaps were filled through strategies or support? How was it done?

**Jiang Zhiwei**: Of course. For example, a classmate didn’t attend a cram school either, but he memorized the model essays from the past ten years by heart and summarized universal templates and corpus on his own. In the end, he also got a high score on the essay. This is how he filled the resource gap through extreme effort and strategy.

**Interviewer**: Based on your experience, give one immediate, actionable suggestion each for students, parents, schools, and platforms.

**Jiang Zhiwei**:

- For students: Make a detailed review plan broken down to the weekly level as early as possible, and use free study apps for self-monitoring.
- For parents: Providing emotional support is far more important than putting pressure on material things. Listen more, be less anxious, and create a quiet, distraction-free study environment for your children.
- For schools: Provide online past exam explanation lectures and essay correction services for postgraduate exam students—this would be a great help.
- For platforms: Optimize the “study mode” of apps to block all entertainment functions with one click and help students focus.

**Interviewer**: If you take online courses again next semester, which three things will you prioritize changing? Why?

**Jiang Zhiwei**:

Start mock exams with past papers earlier instead of only doing section-specific exercises.

Form an online study group of 3-5 people to regularly share notes, discuss difficult questions, and supervise each other.

Install entertainment apps and study apps on separate devices to eliminate any potential distractions.

**Interviewer**: I will briefly recap the key points you shared today. Please confirm if it’s accurate and if there’s anything missing to add.

**Jiang Zhiwei**: Okay.

**Interviewer**: After the interview record is organized, would you be willing to help verify key summaries or supplement materials if needed?

**Jiang Zhiwei**: Yes.

（Note: Encourage interviewees to describe specific situations and timelines; when necessary, use the "cause-action-outcome" structure to follow up.）

## Interviewee 5: Wu Caihong

**Interview Topic**: Interview on Factors Influencing College Students' Online English Learning Outcomes**Interview Time**: September 11, 2024**Interview Location**: Library**Interviewer Name**: Wu Caihong**Age**: 21

### Interview Guidelines

**Interviewer**: 1. This interview aims to understand your experiences and feelings about online English learning in the past semester. It is completely anonymous and only for academic research. You may agree to recording and may stop the interview at any time.2. Please try to use specific stories/situations to explain—when it happened, who was involved, what you did, and what the result was.

**Interviewer**: Please use 2-3 minutes to summarize your online English learning experience in the past semester: where you mainly studied, what methods you used, and what your core goal was.

**Wu Caihong**: This semester, I mainly studied English online at home using a laptop and a phone. I attended classes via Tencent Meeting, and used apps like Baicizhan (Hundred Words Chopping) and Zhenti (Past Exam Questions) for independent practice. My core goals are to pass the CET-4 and CET-6 exams.

**Interviewer**: What are your most frequently used platforms, courses, or foreign teacher services? Why did you choose them?

**Wu Caihong**: I use Baicizhan and Shanbei English most often—they have word banks and mock exams with past papers. I also watch CET-4 tutorial videos on Bilibili. I chose them because they are free and offer targeted training.

**Interviewer**: What is your equipment, internet access, and study space like? How do these conditions affect your learning experience?

**Wu Caihong**: I have a laptop and a tablet. My room is quiet, and my equipment works well. But sometimes my family disturbs me while I’m studying. Overall, the environment allows me to attend classes and solve questions smoothly, but occasional noise distracts me.

**Interviewer**: Looking back at this semester, has there been a time when family support significantly changed your learning outcomes? Please explain it in the order of "cause—what your family did—what you did as a result—outcome".

**Wu Caihong**: The cause was that my CET-4 listening score was very low—I missed the passing line by 10 points in the last exam. My mom陪我 practiced listening for 30 minutes every night after dinner. Even though she doesn’t understand English, she helped me pause the audio and check answers. This made me more persistent in practicing every day and more focused. As a result, a month later, the number of wrong answers in my listening dropped to fewer than 5, and I finally passed CET-4.

**Interviewer**: Emotionally, how does your family’s attitude or companionship make you more motivated or persistent in learning? When did this happen, and what did it bring to your final grades, assignments, or oral English output?

**Wu Caihong**: My dad often says to me, “Just do your best; don’t put too much pressure on yourself.” This attitude helps me relax when I feel anxious before exams. In the last week before the exam, my grades became more stable, and my mindset was calmer.

**Interviewer**: In terms of materials or tools, has your family provided you with equipment, internet access, a dedicated study space, or course fees? Which one had the most direct impact on your learning output? Please give an example.

**Wu Caihong**: My family bought me a tablet and CET-4 past exam books, and let me study in a good environment without disturbing me. The tablet had the most direct impact—it made it easier to read questions and watch tutorial videos.

**Interviewer**: In terms of academics or strategies, has your family worked with you to make plans, check your progress, or practice oral English with you? Tell a specific experience where a change in method led to a change in outcome.

**Wu Caihong**: My mom doesn’t understand English, but she’s good at making plans. In the middle of my exam preparation, my reading progress stalled. She suggested making a four-week reading improvement plan with me. This changed my method—I wrote down the number of reading passages I completed each day in a schedule book instead of just keeping it in my mind. With my mom’s supervision, I was motivated to finish the task every day. After four weeks of persistence, my reading speed and accuracy improved significantly, and my reading score in the mock exam increased a lot.

**Interviewer**: Regarding rules, does your family have agreements on time management or phone use? How are these rules implemented, and what impact do they have on your learning outcomes?

**Wu Caihong**: My parents and I agreed that 7-9 PM every day is my study time. During these two hours, I put my phone in the living room to charge and can’t play with it. My family also doesn’t disturb me during this time, creating a quiet environment. This rule forced me to break the habit of checking my phone every few minutes while studying. At first, it was hard, and I always wanted to use my phone, but after a week, I noticed my focus and efficiency improved a lot. This rule directly ensured high-quality daily study time, laying a solid foundation for CET-4 preparation.

**Interviewer**: If you didn’t have this support, or if the support was reduced, how do you think the outcome would be different? Why?

**Wu Caihong**: The outcome would definitely be much worse—I might not have passed CET-4 on the first try. Because exam preparation is a long and boring process, especially for someone like me who lacks self-discipline. Without my family’s supervision, I would definitely be addicted to the internet and social media, unable to ensure efficient daily study. My grades would gradually drop due to repeated laziness.

**Interviewer**: Have you noticed gaps in learning between classmates due to differences in family support? Can you give a comparative example?

**Wu Caihong**: I’ve seen many such examples. A typical one is my roommate and a classmate from the next dorm. The first classmate has good family conditions—his parents bought him expensive live courses and one-on-one oral English tutoring with foreign teachers. But his family didn’t supervise him strictly—he often stayed up late playing games and slept in class. Those resources were useless to him. The other classmate is from a rural area—his internet signal is unstable, and he can’t afford to buy courses. But his parents attach great importance to his studies; even though they don’t understand English, they call to encourage him every day. He could only rely on the past exam papers provided by the school and free resources, but he was very self-disciplined and went to the library to study every day. As a result, the second classmate passed CET-4 with a high score on the first try, while the first classmate failed several times. This example made me realize that material support is a bonus, but emotional support and self-discipline are the real “hardware.”

**Interviewer**: Please describe a complete cycle of your exam preparation or project learning: how you set goals, arrange time, monitor progress, and review.

**Wu Caihong**: My overall goal was to pass CET-4, which I broke down into: first, expanding my vocabulary to 4,500 words; second, scoring over 160 in both listening and reading. My weekly goal was to complete one set of past papers (and understand them thoroughly) and memorize 200 new words. For time arrangement: I spent 30 minutes every morning using an app to review words, and did one set of past papers in the afternoon when I had no classes. I used a calendar to clock in—checking off tasks every day—and a table to record my accuracy in past papers. Every Sunday, I reviewed by analyzing wrong answers and summarizing which question types had a high error rate, then focused on practicing those types the next week.

**Interviewer**: What are the three most common distractions you encounter while studying—such as social media, noise, or piled-up tasks? What specific strategies do you use to deal with them?

**Wu Caihong**:

Social media distraction (especially Douyin and Kuaishou): I use physical isolation—putting my phone in another room to charge to avoid using it.

Environmental noise: I wear noise-canceling headphones and play music to block out distractions.

Anxiety from unfinished tasks: I break large tasks into small ones to lower the threshold for starting and reduce anxiety.

**Interviewer**: Have you ever had an experience where good self-regulation led to a significant improvement in results? What strategy did you use, how long did you use it, and what changes did it finally bring?

**Wu Caihong**: Yes. In the last month before the exam, I implemented a very strict sprint plan: 1) completing three targeted practice sessions a day (alternating between listening and reading); 2) reviewing wrong answers and new words for 30 minutes every night; 3) completely cutting off games and short videos. I persisted for four weeks. The effect was obvious—my mock exam score rose from around 300 to over 500, and I got a good score in the final exam. The biggest change was that I gained confidence and no longer felt flustered when seeing questions.

**Interviewer**: Also, share an experience where your self-regulation failed. What happened then? How did you adjust and get back on track later?

**Wu Caihong**: Once, my exam preparation wasn’t going well—I was in a bad mood and very anxious, so I gave up completely. I spent three days playing games and watching dramas, feeling guilty but unwilling to study. The direct cause was that I got many wrong answers in the past papers, which made me feel frustrated and think, “I’ll never get better, so I might as well rest.” Later, I started by accepting my emotions—telling myself that occasional laziness is normal and not blaming myself too much. Then I set a tiny goal: only doing one reading passage and memorizing ten words a day. After completing it, I felt a sense of accomplishment, and with that, I gradually increased my workload and got back into my study rhythm.

**Interviewer**: During online learning, when do you clearly feel interested, engaged, happy, or confident? What situations usually trigger these emotions—such as anonymous/semi-anonymous interaction, watching replays, setting your own pace, or getting feedback from teachers?

**Wu Caihong**: I feel very happy and confident when I receive positive feedback from teachers—for example, when the teacher writes comments like “accurate word usage, good progress” on my homework. I feel a great sense of accomplishment and engagement when I can understand a listening passage that I couldn’t before. I also feel happy and interested when I communicate with other classmates and realize I’ve made progress.

**Interviewer**: Tell a story where positive emotions emerged, what actions you took because of them, and finally how your learning output changed.

**Wu Caihong**: In one mock exam, my listening score exceeded 160 for the first time. I was very happy, full of confidence and joy. This good mood made me take the initiative to increase my listening practice—actively finding more difficult materials to practice. In the end, because I practiced more, my listening score stabilized in the high range and I got a high score in the final exam.

**Interviewer**: Did the "physical absence" of the online environment make you more willing to participate or speak up? What did you do, and how did it change your assignments, quizzes, or oral English performance?

**Wu Caihong**: I used to be very shy and afraid of speaking incorrectly. But in Tencent Meeting’s voice discussions, I became very active. I took the initiative to turn on my microphone to answer the teacher’s questions and was willing to share my opinions. Because I didn’t have to be in front of others, I participated more. I got corrections and feedback from the teacher, which improved my oral English. Later, my oral English homework scores were among the top in the class.

**Interviewer**: On the contrary, what situations make you feel anxious, bored, or tired? How do you usually adjust to get back to effective learning?

**Wu Caihong**: I feel anxious when I keep solving question sets but my score doesn’t improve—even drops. I feel bored or tired when I spend a long time on the same type of questions, especially memorizing words. My adjustment method is to put down my books immediately, leave my desk to drink water, listen to music, or take a walk. Then I switch tasks—changing from boring word memorization to watching interesting English short videos. I also use positive self-talk, telling myself that fluctuations are normal and persistence will pay off.

**Interviewer**: Please tell a complete "chain story" for each of the following: 1. Family support leads to changes in self-regulation, which affects learning outcomes; 2. Family support leads to improved positive emotions, which affects learning outcomes.

**Wu Caihong**:

My dad noticed that I stayed up late and had low efficiency, so he suggested I switch to early sleeping and early rising, and supervised me to do so. I accepted his suggestion and made a plan to get up at 6:30 AM to memorize English words. As a result, my morning efficiency was very high, and my memory of essays and words improved greatly.

After I failed a mock exam and felt frustrated, my mom didn’t criticize me—instead, she took me to eat my favorite food and encouraged me to “try again next time.” My family’s tolerance made me feel positive and calm. With a better mindset, I could analyze my wrong answers more calmly, and my subsequent learning efficiency was higher.

**Interviewer**: Based on your experience, which chain is more important to you? Why?

**Wu Caihong**: I think the first chain is more important. Because for a goal like passing CET-4— which requires a lot of review and practice—consistent and stable effort is more important than occasional emotional fluctuations. Family support directly strengthens my self-discipline, ensuring the accumulation of learning, which is the foundation for passing the exam.

**Interviewer**: Do these two chains influence each other? For example, does being more self-disciplined first make you feel better, or does having a better mindset first make it easier to persist? Use a specific story to explain.

**Wu Caihong**: They do influence each other. I persisted in studying for a week first, and the sense of accomplishment from that made me feel better, which made me more willing to continue studying.

**Interviewer**: If we summarize your experience as “family support affects self-regulation and positive emotions, which in turn affects online learning outcomes”, how well do you think this framework fits your situation?

**Wu Caihong**: It fits very well. Family support does affect my self-discipline and emotions, which indirectly affects my learning outcomes.

**Interviewer**: Are there any key factors in your experience that we haven’t mentioned yet—such as classmates, teacher feedback, course difficulty, or evaluation methods? How do they fit into your learning process?

**Wu Caihong**: There are many such factors. First, peer competition is very important. Seeing classmates post high scores in the group makes me anxious, which pushes me to study harder. Second, teacher evaluations—praise from teachers makes me happy and motivated.

**Interviewer**: If you had to rank them, what are the three most critical factors for you? Explain your reasons.

**Wu Caihong**:

Self-regulation: Self-supervision is the most important factor affecting learning efficiency.

Family support: It provides me with encouragement and more motivation.

Positive emotions: My interest and confidence in CET-4 English grow, which affects my learning efficiency.

**Interviewer**: What online English platforms or features have you used—such as adaptive learning, personalized recommendations, live foreign teacher classes, small-group interactive learning, or gamified incentives? Explain why you chose them.**Wu Caihong**: I’ve used many online English platforms, such as Baicizhan, Shanbei, English Listening apps, and Bilibili listening courses. I chose them because they are targeted and free.

**Interviewer**: Choose the feature you use most often or find most memorable. Talk about how it changed your goal-setting, progress monitoring, emotional experience, or participation methods, and finally what learning results it brought.

**Wu Caihong**: It’s the “Word TV” feature in Baicizhan. It uses videos to help memorize words, allowing me to make more associations and remember words more firmly. My vocabulary expanded, which helped with reading and listening.

**Interviewer**: Do these technologies have any side effects—such as interrupted attention, information overload, or anxiety? How do you balance the pros and cons?**Wu Caihong**: Yes, there are side effects. Sometimes, ads pop up and distract me. But later, I turned off notifications and only used the apps at fixed times, which balanced the side effects.

**Interviewer**: Compared with your classmates, what advantages or limitations do you have in terms of equipment, internet access, study space, opportunities for courses or foreign teachers, and family attitudes?

**Wu Caihong**: Advantages: Good home internet, a dedicated room, parents who don’t disturb me and even bought me live courses. Limitations: Sometimes studying alone in my room makes me lack self-discipline, and I feel like resting.

**Interviewer**: How do these differences lead to gaps in learning outcomes at critical times, such as during exam preparation, important assignments, or projects? Please give an example.

**Wu Caihong**: For example, some classmates have one-on-one oral English classes with foreign teachers, so their oral English and listening are better than mine. They express themselves more fluently in group discussions.

**Interviewer**: Have you seen examples where gaps were filled through strategies or support? How was it done?

**Wu Caihong**: Some classmates found free online resources to practice oral English on their own, and their oral English improved a lot. They also practiced listening repeatedly, and their listening scores increased. I think the key is finding the right method and persisting.

**Interviewer**: Based on your experience, give one immediate, actionable suggestion each for students, parents, schools, and platforms. Explain what to do, how to do it, and how often.

**Wu Caihong**:

- For students: Study at fixed times every day, check and reflect on learning outcomes regularly.
- For parents: Don’t put too much pressure on children; encourage them more and provide a quiet environment for studying.
- For schools: Provide more past exam resources and mock exam platforms.
- For platforms: Launch more free thematic courses.

**Interviewer**: If you take online courses again next semester, which three things will you prioritize changing? Why?

**Wu Caihong**:

Make a more detailed weekly plan, because my current plan doesn’t fit my personal situation well.

Join a study group to supervise each other, which will make me more self-disciplined.

Take a mock exam every day to test my learning outcomes and see if I’ve made progress.

**Interviewer**: I will briefly recap the key points you shared today. Please confirm if it’s accurate and if there’s anything missing to add.

**Wu Caihong**: It’s accurate; nothing is missing.

**Interviewer**: After the interview record is organized, would you be willing to help verify key summaries or supplement materials if needed?

**Wu Caihong**: I am willing.

**Interviewer**: Okay, thank you.

## Interviewee 6: Long Xuyi

**Interview Topic**: Interview on Factors Influencing College Students' Online English Learning Outcomes**Interview Time**: September 11, 2024**Interview Location**: Library**Interviewer Name**: Long Xuyi**Age**: 21

### Interview Guidelines

**Interviewer**: 1. This interview aims to understand your experiences and feelings about online English learning in the past semester. It is completely anonymous and only for academic research. You may agree to recording and may stop the interview at any time.2. Please try to use specific stories/situations to explain—when it happened, who was involved, what you did, and what the result was.

**Interviewer**: Please use 2-3 minutes to summarize your online English learning experience in the past semester: where you mainly studied, what methods you used, and what your core goal was.

**Long Xuyi**: I usually study in the library, dormitory, or classroom, using a phone, computer, or tablet for independent learning. My core goal is to master basic English vocabulary and be able to communicate simply with others.

**Interviewer**: What are your most frequently used platforms, courses, or foreign teacher services? Why did you choose them?

**Long Xuyi**: I usually use Baicizhan to learn English words—to expand my vocabulary and have more options when communicating with others. I also use Duolingo, which adds fun to English learning, making it less boring and helping me feel the joy of learning English.

**Interviewer**: What is your equipment, internet access, and study space like? How do these conditions affect your learning experience?

**Long Xuyi**:My devices are generally electronic, and the internet doesn’t have much to do with my study space. Because I can learn vocabulary even without an internet connection. I usually study alone when others are around, which motivates me more. Moreover, using fragmented time to learn English is more effective and time-saving.

**Gao Muyang**:Looking back on this semester, is there an incident where your family’s support significantly changed your learning outcome? Please tell the full story in terms of cause, what your family did, what you did as a result, and the outcome.

**Long Xuyi**:My family’s support increased my desire to learn English. They believed that mastering English is crucial in today’s society as part of my future planning. They would also casually ask me how to say daily items in English or share their work experiences involving English. Influenced by them, I realized the importance of English and began to review the knowledge I had previously neglected.

**Gao Muyang**:Emotionally, how did your family’s attitude and companionship make you more motivated or persistent? When did this happen, and what was the reflection in your final grades, assignments, or oral output?

**Long Xuyi**:I learn some English words and master difficult sentences every day. My family asks about my study progress daily, and I report it in detail. Their interest makes me more motivated and accomplished, helping me persist in learning English for a longer time.

**Gao Muyang**:Materially, did your family provide equipment, internet, a fixed study space, or course fees? Which one had the most direct impact on your learning output? Please give an example.

**Long Xuyi**:In today’s society, if you truly want to learn a language seriously, it’s not difficult. So, if the equipment or internet provided by my family is necessary for my needs, I will ask for money to take courses or buy materials.

**Gao Muyang**:Academically, did your family jointly制定 plans, check progress, or practice oral English with you? Please share a specific experience where a change in method led to a change in results.

**Long Xuyi**:After learning about my English learning experience, my family sometimes asks me questions in Chinese and requires me to answer in English. I then use AI to record our conversation, analyze the flaws in my speech, correct them, and try to express myself completely in the next oral practice.

**Gao Muyang**:Regarding rules or systems, did your family have agreements on time management or mobile phone use? How were these rules implemented, and what impact did they have on your learning outcomes?

**Long Xuyi**:Since I’m an adult now, my family isn’t very strict about my time management or mobile phone use. However, because they set strict rules when I was younger, I can now control these aspects myself. During my studies, I try to reduce time spent on games or videos and use it for learning or researching information instead.

**Gao Muyang**:If you hadn’t received or had less of the above support, how do you think the outcome might have been different? Why?

**Long Xuyi**:I think I might not have persisted in learning English and would have given up halfway. Because my self-control isn’t very strong, it’s only with the supervision of family and friends that I could maintain my daily English learning routine.

**Gao Muyang**:Have you observed learning gaps among classmates due to differences in family support? Can you give a comparative example from your surroundings?

**Long Xuyi**:Yes, I can clearly see such gaps. For example, when I was in primary and junior high school, my teacher’s children had already mastered all junior high school vocabulary and could converse fluently with foreigners. This was not only because their parents used English in daily communication but also because they specially spent money on extracurricular classes to support their learning.

**Gao Muyang**:Please describe a complete cycle of your exam preparation or project learning, including how you set goals, arrange time, monitor progress, and review.

**Long Xuyi**:For example, when preparing for CET-6, I set a complete timeline from the current month to the exam date. I divided my goals sequentially, allocated corresponding time for each goal, and monitored whether I completed the monthly targets. At the end of each month, I reviewed what I did well and what needed improvement.

**Gao Muyang**:What are the three most common distractions you encounter in learning, such as social media, noise, or task accumulation? What specific strategies have you adopted to cope with them?

**Long Xuyi**:For instance, when using my phone to memorize words, I might accidentally switch to other social platforms and forget about memorization, getting distracted by entertainment. To address this, I downloaded an app that locks all entertainment software until I finish my tasks.

Second, noise. When I’m memorizing words or reciting English articles outside and get distracted by classmates’ noise, I find another quiet place or put on noise-canceling headphones to immerse myself in learning. If tasks pile up, I prioritize them into key, secondary, and less important ones, finish the key tasks first, and then complete the rest if time permits.

**Gao Muyang**:Is there an experience where good self-regulation significantly improved your results? What strategy did you use, how long did it last, and what changes occurred in the end?

**Long Xuyi**:When preparing for an exam, in the last month, I refined my English learning plan to every hour. If I didn’t complete a task, there would be a penalty. Because time was limited, I strictly followed the plan from the last month until the exam, resulting in my ideal score.

**Gao Muyang**:Please also share an experience where self-regulation failed. What happened then? How did you recover and get back on track later?

**Long Xuyi**:When preparing for an English exam, due to the long timeline, I was inefficient. I didn’t finish half my daily tasks but thought there was still plenty of time, so I piled them up. Eventually, many tasks remained unfinished as time became tight. I then urgently categorized my strengths and weaknesses, spent less time consolidating my strengths, and more time learning and mastering my weaknesses.

**Gao Muyang**:In online learning, when do you clearly feel interested, engaged, happy, or confident? What situations usually trigger these emotions?

**Long Xuyi**:In online learning, I feel particularly interested when communicating with netizens. Discussing a problem together stimulates our motivation and interest in learning. Additionally, receiving feedback from teachers and turning unclear knowledge into my own also gives me a strong sense of accomplishment.

**Gao Muyang**:Please share an experience where positive emotions emerged, what actions you took as a result, and how your learning output changed.

**Long Xuyi**:When my friends ask me English questions and I can answer them completely with detailed explanations to help them understand, I feel more positive emotions. This makes me more motivated and interested in learning English.

**Gao Muyang**:Do you feel more willing to participate or speak up due to the physical absence in the online environment? What did you do then, and how did your subsequent assignments, tests, or oral performance differ?

**Long Xuyi**:Yes, I am more willing to participate in discussions online. If I miss part of the knowledge system, I will relearn, master, and consolidate what the teacher taught, and explore English learning methods on my own. My subsequent assignments, tests, and oral performance may not be very satisfactory, but I actively ask teachers, friends, and classmates for help to improve.

**Gao Muyang**:Conversely, what situations cause anxiety, boredom, or fatigue? How do you usually adjust and return to an effective learning state?

**Long Xuyi**:When I can’t remember or mix up certain English words for a week, I feel anxious, bored, and tired. At this point, I list the words, learn them in interesting contexts to enhance memory, then relax by listening to English songs to recover my effective learning state.

**Gao Muyang**:Please tell a complete chain story for each: 1. Family support → change in self-regulation → learning outcome. 2. Family support → improvement in positive emotions → learning outcome.

**Long Xuyi**:In daily life, my parents often discuss my future career plans and the important role of learning English. This makes me pay more attention to English learning methods and improve my mastery of English.

For the second chain, my family’s support has given me positive feedback in my English learning. My parents encourage me and actively ask for my advice, which greatly boosts my sense of accomplishment and satisfaction. Thus, I study harder and my English grades improve further.

**Gao Muyang**:From your experience, which chain is more critical to you? Why?

**Long Xuyi**:The second chain is more critical to me. Because learning English is a long-term process driven not only by personal motivation but also by family companionship and friends’ support. Integrating English learning into daily life through their support makes it an important part of daily life.

**Gao Muyang**:Do these two chains interact with each other? Please illustrate with a specific example.

**Long Xuyi**:Yes, they do. For example, when I reflexively pick up my phone to memorize words every morning, finishing the words gives me a great sense of accomplishment. This puts me in a good mood, making me feel I’ve taken another step toward English proficiency. A positive mindset strengthens my motivation, so I study English with greater interest, invest more energy, and persist more easily.

**Gao Muyang**:If your experience is summarized as "family support influences self-regulation and positive emotions, thereby affecting online learning outcomes," how well does this framework align with your situation?

**Long Xuyi**:I think the alignment is about 70%. Because family support is closely linked to self-regulation and positive emotions; they cannot exist independently but interact with each other. This has a deeper impact on English learning and becomes more subtle in daily life.

**Gao Muyang**:In your experience, are there any key factors not mentioned earlier, such as peers, teacher feedback, course difficulty, or evaluation methods? How do they enter your learning process?

**Long Xuyi**:A key factor not mentioned is peers. Because peers spend the most time with you at school. When peers share your interest in learning English, they integrate into daily life. Whether through daily communication or discussing problems, it’s easy for both to find correct answers together, boosting enthusiasm and improving English grades.

**Gao Muyang**:If you had to rank them, what are the three most critical factors for you, and why?

**Long Xuyi**:The three most critical factors are family support, peers, and course difficulty. First, family support provides the initial motivation to start learning English. Peers are the long-term motivation to persist. Third, course difficulty helps assess my progress at different stages of English learning.

**Gao Muyang**:What online English platforms or features have you used, such as adaptive learning, personalized recommendations, foreign teacher live broadcasts, small-group interaction, or gamified incentives? Why did you choose them?

**Long Xuyi**:I’ve used foreign teacher live broadcasts because they involve human interaction, enhancing the communicative aspect of English learning. Learning becomes dynamic rather than solitary reflection. External help makes English more understandable and conducive to mastering the subject.

**Gao Muyang**:Please select a feature you recently used frequently or were most impressed by. Explain how it changed your goal-setting, progress monitoring, emotional experience, or participation, and ultimately what learning outcomes it brought.

**Long Xuyi**:Foreign teacher live broadcasts dynamically monitor my learning process. Authoritative experts in English help me objectively assess my progress, improve my learning experience, and make me actively participate. This not only makes my English learning more enjoyable but also advances my knowledge system.

**Gao Muyang**:Have these technologies caused any side effects, such as interrupted attention, information overload, or anxiety? How do you balance the pros and cons?

**Long Xuyi**:Yes, they have. Information overload is the most common issue. Since my English level may not keep up with others, I don’t feel anxious or discouraged. Instead, I list the information I can’t understand or process separately, absorb part of it first, and learn the rest when I’m more capable.

**Gao Muyang**:Compared with classmates around you, what advantages or limitations do you have in terms of equipment, internet, study space, courses, foreign teacher opportunities, or family attitudes?

**Long Xuyi**:My advantages are equipment, internet, study space, and some courses, as my family fully supports my English learning with time, space, and money. My limitations are foreign teacher opportunities and daily English communication with native speakers, as my parents are not proficient in English.

**Gao Muyang**:How do these differences manifest as gaps in learning outcomes at key times, such as exam preparation or important assignments/projects? Please give an example.

**Long Xuyi**:I think the gap is smaller during exam preparation but more evident in projects. Because projects require not only English knowledge but also communication skills with others.

**Gao Muyang**:Have you observed cases where gaps were bridged through strategies or support? How?

**Long Xuyi**:Yes. Some classmates have weak English foundations but are proficient in daily communication. This is because their parents took them to live abroad, allowing them to communicate with native speakers daily without alienation. However, they lack systematic learning of Chinese-style English education and are less familiar with basic knowledge.

**Gao Muyang**:Based on your experience, please give one most worthwhile suggestion for students, parents, schools, or platforms respectively. Explain what to do, how to do it, and the frequency.

**Long Xuyi**:For students: Start with daily learning—memorize words first, clarify study plans, and persist in learning English every day.For parents: Provide a good learning environment with time, space, and financial support. Avoid disturbing study progress; check in once a week.For schools: Strengthen students’ English knowledge system and practical skills, such as oral English, 2-3 times a week.For platforms: Create communication groups for English learners to discuss deeply.

**Gao Muyang**:If you have online English learning next semester, what three things will you prioritize changing? Why?

**Long Xuyi**:

- Adjust study time: Use both fragmented and dedicated time to strengthen weaknesses.
- Enhance interaction: Improve oral skills through communication with others.
- Participate in competitions: Boost confidence in applying English.

**Gao Muyang**:I will briefly recap the key points you shared today. Please confirm if they are accurate and if anything is missing.

**Long Xuyi**:Accurate, no additions needed.

**Gao Muyang**:After organizing the interview records, would you be willing to assist in verifying key summaries or supplementing materials if needed?

**Long Xuyi**:Yes.

**Gao Muyang**:Thank you.

**Date**: 2024.9.11**Location**: Library**Interviewer**: Wang Xiaoguo, Age: 21

### Interview Guidelines:

**Gao Muyang**: 1. This interview aims to understand your online English learning experiences and feelings in the recent semester; it is completely anonymous and for academic research only; you may agree to recording and may terminate at any time.Please use specific stories/situations to illustrate (when it happened, who participated, what you did, what the result was).

**Gao Muyang**: Please summarize your online English learning experience in the recent semester in 2-3 minutes, including where you mainly studied, how you studied, and your core goal.

**Wang Xiaoguo**: My recent online English learning experience was mainly in the library. I listened to Liu Xiaoyan’s online full English course, and my core goal was to master basic grammar and sentence structure for future study abroad.

**Gao Muyang**: What are your most frequently used platforms/courses/foreign teacher services? Why did you choose them?

**Wang Xiaoguo**: I chose Bilibili because the course I bought included access to Bilibili content.

**Gao Muyang**: What is the general situation of your equipment, internet, and study space? How do these conditions affect your learning experience?

**Wang Xiaoguo**: I have three devices: a tablet, a phone, and a computer. Generally, the phone has the biggest impact on learning because I sometimes want to quit studying and do other irrelevant things. The tablet is good for learning, doesn’t interfere, allows zero head-down time, and is very efficient. For the internet, it’s usually stable; I download learning materials in advance.I only use the internet for actual exercises or when going out to eat. My study space is usually the library. Studying in the dorm is passive, but the library has a good environment that improves efficiency.

**Gao Muyang**: Looking back on this semester, is there an incident where your family’s support significantly changed your learning outcome? Please tell the full story in terms of cause, what your family did, what you did as a result, and the outcome.

**Wang Xiaoguo**: Yes. My old electric bike tire often broke, so my family spent a lot of money to buy me a new one. Now I can ride it conveniently every day, saving me from the trouble and time of repairing the bike. This makes it easier for me to go to the library to study every day, improving my learning efficiency.

**Gao Muyang**: Emotionally, how did your family’s attitude or companionship make you more motivated or persistent? When did this happen, and what was the reflection in your final grades, assignments, or oral output?

**Wang Xiaoguo**: Emotionally, my family’s attitude has always been my strongest support. My parents’ attitude toward my study is to do my best; they will always be my foundation and provide help within their means. This is not guilt-based education. I usually listen to them, study hard, and get good grades.

**Gao Muyang**: Materially, did your family provide equipment, internet, a fixed study space, or course fees? Which one had the most direct impact on your learning output? Please give an example.

**Wang Xiaoguo**: Materially, my family provided equipment, internet, a fixed study space, and course fees. I think course fees had the most impact on learning output. There is no upper limit on my course fees, and only without restrictions can I take risks and gain better platforms, materials, and methods.

**Gao Muyang**: Academically, did your family jointly制定 plans, check progress, or practice oral English with you? Please share a specific experience where a change in method led to a change in results.

**Wang Xiaoguo**: Academically, my family jointly制定 plans. Because my oral English was not good in junior high school, my parents found me a one-on-one tutor. I got good grades in the English exam in junior high school.

**Gao Muyang**: Regarding rules or systems, did your family have agreements on time management or mobile phone use? How were these rules implemented, and what impact did they have on your learning outcomes?

**Wang Xiaoguo**: Regarding systems, my family has no agreements on mobile phone use time. Because I rarely use my phone, usually for extracurricular reading or outdoor sports. Having a phone or not doesn’t affect my study much; I usually use a tablet for learning.

**Gao Muyang**: If you hadn’t received or had less of the above support, how do you think the outcome might have been different? Why?

**Wang Xiaoguo**: I think it would be different. Because every cause has an effect; changing the cause will inevitably change the effect—this is a philosophical principle.

**Gao Muyang**: Have you observed learning gaps among classmates due to differences in family support? Can you give a comparative example from your surroundings?

**Wang Xiaoguo**: Yes. In high school, a classmate of mine had divorced parents, which made him listless, depressed, and less enthusiastic about studying. His grades were at the bottom of the class. The teacher often talked to him alone, but he always made excuses about being from a single-parent family and refused to study.

**Gao Muyang**: Please describe a complete cycle of your exam preparation or project learning, including how you set goals, arrange time, monitor progress, and review.

**Wang Xiaoguo**: I’ll talk about my recent exam preparation plan. I usually get up at 7 a.m., review the previous day’s content in the library at 7:30 a.m., eat breakfast at 8 a.m., start the first study session at 9 a.m. until 11:30 a.m., eat lunch, study for a short period from 12 p.m. to 2 p.m., rest for five minutes and continue until 5:30 p.m., eat dinner, rest for half an hour, then summarize from 6 p.m. to 10 p.m. and make a brief plan for the next day.

**Gao Muyang**: What are the three most common distractions you encounter in learning, such as social media, noise, or task accumulation? What specific strategies have you adopted to cope with them?

**Wang Xiaoguo**: My three biggest distractions are: first, conflicting schedules with roommates; second, ineffective socializing; third, worrying about what to eat every day. To deal with conflicting schedules, I usually make up for sleep in the library if I don’t sleep well. For ineffective socializing, I gradually learn to refuse to avoid wasting time.

**Gao Muyang**: Is there an experience where good self-regulation significantly improved your results? What strategy did you use, how long did it last, and what changes occurred in the end?

**Wang Xiaoguo**: Yes. In high school, there was a placement exam at the end of the first year. To get into the best class, I pushed myself every day. I washed my face with cold water in the morning and evening, saving a lot of study time, and got good grades.

**Gao Muyang**: Please also share an experience where self-regulation failed. What happened then? How did you recover and get back on track later?

**Wang Xiaoguo**: Failure in self-regulation usually comes from too much pressure. When I’m under too much pressure, I often become sensitive and fragile. At this time, I go out for a walk, sit on the grass, listen to music, and adjust my mood.

**Gao Muyang**: In online learning, when do you clearly feel interested, engaged, happy, or confident? What situations usually trigger these emotions, such as anonymous/semi-anonymous interaction, recorded playback, autonomous pacing, or teacher feedback?

**Wang Xiaoguo**: In online learning, my attention is usually not focused. At this time, the extracurricular examples given by teachers or hosts are vivid and attractive, and the rhythm gradually returns to normal. My mood also becomes happy.

**Gao Muyang**: Please share an experience where positive emotions emerged, what actions you took as a result, and how your learning output changed.

**Wang Xiaoguo**: Positive emotions usually arise when I master a certain skill proficiently, leading to a virtuous cycle that constantly motivates me. I will then study that skill harder, improving my ability.

**Gao Muyang**: Do you feel more willing to participate or speak up due to the physical absence in the online environment? What did you do then, and how did your subsequent assignments, tests, or oral performance differ?

**Wang Xiaoguo**: Physical absence makes me less willing to participate in discussions because speaking will probably waste my time. I hope to use my time for review and study. Subsequent assignments and tests usually develop in a good direction.

**Gao Muyang**: Conversely, what situations cause anxiety, boredom, or fatigue? How do you usually adjust and return to an effective learning state?

**Wang Xiaoguo**: Some difficult learning content causes anxiety, boredom, and fatigue. Difficult content usually has playback, but if I still can’t understand it after multiple replays, I usually close the software, take a deep breath, play some music, and adjust my learning state.

**Gao Muyang**: Please tell a complete chain story for each: 1. Family support → change in self-regulation → learning outcome. 2. Family support → improvement in positive emotions → learning outcome.

**Wang Xiaoguo**: The first is family financial support → change in self-regulation → learning outcome. My family’s support is generally financial, making me realize the importance of learning and study wholeheartedly, thus improving my grades.The second is family support → improvement in positive emotions → learning outcome. Family financial support also makes me study in a happy mood. The result of happy learning is obviously different from forced learning.

**Gao Muyang**: From your experience, which chain is more critical to you? Why?

**Wang Xiaoguo**: I think the second chain is more critical because I am more emotional; I believe I should follow what my heart decides. Positive emotions generally have a positive impact on learning outcomes.

**Gao Muyang**: Do these two chains interact with each other, such as becoming more disciplined first and then in a better mood, or being in a more positive mindset first and thus easier to persist? Please illustrate with a specific example.

**Wang Xiaoguo**: I don’t think the two chains interact. Becoming more disciplined and then in a better mood is because you have goals; achieving them makes you happy. Being in a more positive mindset first and thus easier to persist is because you first coax yourself, constantly deluding yourself to persist. Fundamentally, one is goal-oriented, and the other is content-oriented.

**Gao Muyang**: If your experience is summarized as "family support influences self-regulation and positive emotions, thereby affecting online learning outcomes," how well does this framework align with your situation?

**Wang Xiaoguo**: I think the alignment is about 50%. Family support is only the first step in my learning; how, when, and what to learn are all explored by myself.

**Gao Muyang**: In your experience, are there any key factors not mentioned earlier, such as peers, teacher feedback, course difficulty, or evaluation methods? How do they enter your learning process?

**Wang Xiaoguo**: Yes, teacher feedback is crucial to me. When I was young, I was very naughty and always made strange moves to attract the teacher’s attention, thinking it would make me stand out. But after a private talk with the teacher, I realized this behavior was not only childish but also affected others. Since then, I have become quiet and studied hard at school.

**Gao Muyang**: If you had to rank them, what are the three most critical factors for you, and why?

**Wang Xiaoguo**: They are teacher feedback, internal learning motivation, and evaluation methods. Both teacher feedback and evaluation methods are feedback that can stimulate us to learn. Internal learning motivation is the fundamental source of our continuous learning.

**Gao Muyang**: What online English platforms or features have you used, such as adaptive learning, personalized recommendations, foreign teacher live broadcasts, small-group interaction, or gamified incentives? Please tell us why you chose them.

**Wang Xiaoguo**: I only use Bilibili and Duolingo for English learning. Generally, I study offline. English is a highly oral subject; cultivating oral skills and grammar requires face-to-face interaction. I think Duolingo is good because it allows real-time translation and dialogue interaction.

**Gao Muyang**: Please select a feature you use most frequently or were most impressed by. Explain how it changed your goal-setting, progress monitoring, emotional experience, or participation, and ultimately what learning outcomes it brought.

**Wang Xiaoguo**: The most impressive feature is connecting with foreign teachers. It not only helps us meet foreign friends but also exercises our oral English skills, improving our ability to speak without fear, hesitation, or mistakes.

**Gao Muyang**: Have these technologies caused any side effects, such as interrupted attention, information overload, or anxiety? How do you balance the pros and cons?

**Wang Xiaoguo**: Yes, they have. For example, they make me anxious. When I’m anxious, I usually close these software to avoid being upset.

**Gao Muyang**: Compared with classmates around you, what advantages or limitations do you have in terms of equipment, internet, study space, courses, foreign teacher opportunities, or family attitudes?

**Wang Xiaoguo**: Compared with classmates, my equipment is better. In terms of internet and study space, everyone is about the same at school. But in terms of foreign teacher opportunities and family attitudes, my dad has always wanted me to study abroad, but my想法 is different.

**Gao Muyang**: How do these differences manifest as gaps in learning outcomes at key times, such as exam preparation or important assignments/projects? Please give an example.

**Wang Xiaoguo**: Maybe only differences in equipment and internet affect learning outcomes. If equipment or internet is laggy, it will affect mood, thereby affecting learning efficiency and results.

**Gao Muyang**: Have you observed cases where gaps were bridged through strategies or support? How?

**Wang Xiaoguo**: Yes. My senior sister didn’t have good grades at first, but she studied hard in the library from 7 a.m. to 10 p.m. every day for postgraduate entrance exams. Diligence can make up for lack of talent, and she was eventually admitted to Guangxi Normal University.

**Gao Muyang**: Based on your experience, please give one most worthwhile suggestion for students, parents, schools, or platforms respectively. Explain what to do, how to do it, and the frequency.

**Wang Xiaoguo**: For students: Keep repeating what you got wrong.For parents: Understand what students need and how to provide the best environment.For schools: Listen to students’ voices and adjust policies in a timely manner.For platforms: Pay more attention to negative reviews because failure comes from communication.

**Gao Muyang**: If you have online English learning next semester, what three things will you prioritize changing? Why?

**Wang Xiaoguo**: During the CET-4 exam preparation, he formulated a detailed study plan, asked his family to help supervise him, and actively reflected on himself.

**Gao Muyang**: I will briefly recap the key points you shared today. Please confirm if they are accurate and if anything is missing.

**Wang Xiaoguo**: Okay.

**Gao Muyang**: After organizing the interview records, would you be willing to assist in verifying key summaries or supplementing materials if needed?

**Wang Xiaoguo**: Yes.

**Time:September 15, 2024**

**Venue:Athletics field**

**Interviewer’s name: Meng**

**Age:20**

Gao Muyang: Have you had any online English learning experiences this semester?

Meng: I use apps to study.

Gao Muyang: When you study English through apps, what do you specifically learn? Do you have any goals?

Meng: Initially, I wanted to study for the CET-4 exam, and then to help with postgraduate English studies.

Speaker 1 - 0:38

Gao Muyang: What apps or courses do you prefer, and why did you choose them?

Meng: First, with app-based learning, if you can’t keep up with video learning, you can’t adjust it. But with apps, you can repeatedly study, answer questions, and review, which is more convenient. It’s also more interesting than just watching videos and not as boring. For example, the English learning app I use is called Duolingo.

Gao Muyang: What about your study equipment, internet access, and learning space—where do you usually study, and what’s the general situation? Do you think these affect your learning experience?

Meng: I usually use my phone and tablet for studying. Sometimes I study in the dorm when I have time, and sometimes I do fragmented learning during free moments. As for the impact on learning, one issue is that fragmented learning may not lead to strong memory retention. However, if I set aside fixed time for study, the efficiency is much better.

Gao Muyang: Has your family done anything to support your recent studies, and have you noticed any changes?

Meng: My family has always supported my studies and tried their best to satisfy my needs for things I require. The results have been more or less the same.

Gao Muyang: In terms of your family’s attitude, have they provided companionship in your studies?

Meng: My family is usually very busy, so I mostly study alone. There’s not much companionship.

Gao Muyang: Has your family provided material support? If so, what do you think is the most direct impact?

Meng: Materially, they provide funding. The most direct impact is that I don’t have to worry about subsequent expenses, such as app memberships or study equipment. For example, if I need a tablet or a new computer, I don’t have to pay for it myself—my family supports me.

Gao Muyang: Have you discussed your English learning plan with your family, or do they ask about your progress?

Meng: I mostly study independently; my family doesn’t know much about it. They occasionally ask if I’ve read books today, but that’s about it.

Gao Muyang: Regarding time management and phone usage, does your family ask you to use your phone less or limit your screen time?

Meng: Not really. They don’t really interfere with my phone usage.

Gao Muyang: If you didn’t have your family’s support, do you think things would be different?

Meng: Definitely. There are things I might not be able to obtain, which could halve my learning efficiency or force me to change my study methods.

Gao Muyang: Have you observed gaps in academic performance among classmates due to differences in family support?

Meng: Yes, I have. For example, classmates without family support may have limited access to study materials, while those with support may have a broader perspective or more resources. They also have more time to explore additional information.

Gao Muyang: Can you share a complete study cycle or plan you use for exam preparation?

Meng: For English learning, I usually review words I learned the previous day for ten minutes in the morning. Then, during fragmented time, I learn new content, participate in app challenges, or do other interesting activities. I review my progress once a week. If I studied a lot that week, I set the same progress as a goal for the next week. If I had less time, I adjust the next week’s content to get closer to my ultimate goal.

Gao Muyang: Have you ever felt that your self-regulation was particularly good, leading to significant improvement during that period?

Meng: Definitely. When self-regulation is strong, I learn more content, which greatly improves my grades or reading skills.

Gao Muyang: What methods did you use, and how long did it last?

Meng: The longest was last semester, when I prepared for CET-4 and CET-6 using Duolingo. Although I didn’t pass CET-4, I got a relatively good score.

Gao Muyang: Can you share an experience where your self-regulation was unstable?

Meng: For example, when studying on the phone, I might get distracted and switch to other apps. By the time I realize it, a lot of time has passed, and I can’t finish my daily tasks. Later, I set restrictions on my phone—like limiting video or game apps to a certain time per day. Knowing I can’t use them again that day motivates me to finish my goals first before playing.

Gao Muyang: What are the three most common distractions you encounter while studying?

Meng: One is seeing others around me not studying, which makes it hard to focus. Another is the internet’s appealing content, which makes me involuntarily spend a lot of time on it, reducing learning effectiveness.

Gao Muyang: When do you feel particularly engaged or confident in online learning?

Meng: When I have a smooth study session with no difficulties. If I grasp a knowledge point easily without getting stuck, I feel happy and satisfied.

Gao Muyang: Can you share a time when you were highly motivated to study and the resulting behaviors and outcomes?

Meng: Sometimes, when I’m in the mood to study, I might spend half the day learning. Being fully engaged helps me consolidate knowledge through review, and I feel happy with the results.

Gao Muyang: Do you think you’re more willing to ask questions online because you’re not physically present? What’s the difference between online and offline in this regard?

Meng: Online, I feel less nervous because it’s not face-to-face, so I have more room to experiment. Offline, the presence of classmates can make me anxious and affect my willingness to speak. Online, since no one can see me, I can more freely clarify doubts.

Gao Muyang: When does online learning make you feel bored or anxious?

Meng: When I’ve studied hard for a long time online but find no improvement in offline tasks like doing exercises or reading. This makes me anxious and tired, thinking I’ve wasted my time.

Gao Muyang: How do you recover and continue studying after feeling this way?

Meng: I usually let myself rest for a while, then review my plans. If there are problems, I adjust them. If not, I try studying again. If I get good feedback, I continue; otherwise, I re-evaluate and adjust.

Gao Muyang: Can you share an experience where family support plus self-discipline led to positive results?

Meng: When my family supports me financially for learning, I become more self-disciplined. Even if the results aren’t always good, the process is valuable because I learn a lot.

Gao Muyang: How do you think emotional support from your family would affect your learning outcomes?

Meng: It would definitely be better. Emotional support puts me in a better mood, so I don’t feel burdened. I can study happily, leading to a more relaxed environment.

Gao Muyang: Which do you think is more critical—material or emotional support?

Meng: Both are important, but emotional support is key. Encouragement means more than just giving money.

Gao Muyang: Do you think these two types of support interact?

Meng: Yes. A good mood makes me want to study, so I’ll open my app and learn English. Being in a good state makes it easier to persist. If I’m in a bad mood, studying feels tiring and inefficient.

Gao Muyang: Does the framework “family support influences self-regulation and positive emotions, which in turn affect online learning outcomes” align with your experience?

Meng: Not entirely. My family provides financial support but little emotional companionship because they’re busy.

Gao Muyang: Are there any other critical factors we haven’t mentioned?

Meng: Yes, like app quality. I’ve used boring apps that didn’t help much. Also, teacher quality—if a teacher isn’t suitable, my progress is slow.

Gao Muyang: If you had to rank the top three most critical factors for your learning, what would they be and why?

Meng: First, study conditions. Without good conditions, you can’t access follow-up resources. Second, quality of learning (app/teacher quality). Third, difficulty progression, which varies by individual.

Gao Muyang: What online platforms or features have you used?

Meng: Duolingo, and I’ve watched some teachers’ live broadcasts, which I found interesting.

Gao Muyang: What’s the most memorable feature?

Meng: Duolingo’s ranking function. If I study a lot in a week, I can be at the top. Competition motivates me to learn harder to keep my position.

Gao Muyang: Have these technologies had any negative impacts?

Meng: Yes. Daily check-ins feel restrictive—missing a day breaks the streak, which frustrates me. Sometimes I rush to check in without really learning. Also, incorrect information in apps can’t be verified easily.

Gao Muyang: How do you balance these drawbacks?

Meng: I don’t be too hard on myself—occasional relaxation is okay. If I notice conflicting information, I use online resources to verify it.

Gao Muyang: Compared to classmates, do you think your equipment, internet, or learning space is an advantage or a limitation?

Meng: About the same, but slightly limiting. Some classmates have one-on-one tutors, which I’m not comfortable with—I prefer self-study.

Gao Muyang: Do these differences lead to gaps in learning outcomes?

Meng: A bit. Self-study makes it easy to be lazy. Classmates with supervision have better results and progress faster. I might be slower or want to give up, with no one to encourage me.

Gao Muyang: Have you seen cases where strategies or family support made up these gaps?

Meng: It mainly depends on self-regulation. If you truly want to learn, you’ll work hard. If not, even family support won’t help.

Gao Muyang: Based on your experience, what advice would you give to students, parents, or schools?

Meng: Students should ask themselves what they want to gain from studying. Parents should provide more companionship and encouragement, not criticism—too much pressure causes rebellion. Schools should help teachers learn new teaching methods to benefit students.

Gao Muyang: If you continue online English learning, what would you change first and why?

Meng: I might adjust study time and methods. Setting a regular daily schedule, not too long or short, based on last semester’s evaluation.

- Interview Time: September 11, 2025
- Interviewer: JTC
- Interviewee: Zeng Junjie

Interview Content:

JTC: This interview aims to understand your online English learning experiences and feelings this semester. It is completely anonymous and for academic research only. You may agree to be recorded and may terminate the interview at any time.

JTC: Please use specific stories/situations to illustrate (when it happened, who was involved, what you did, and what the result was).

Warm-up and Learning Profile

JTC: Please summarize your online English learning experiences this semester in 2-3 minutes: where you mainly studied, how you studied, and your core goal.

ZJJ: I mainly studied English through apps like “Bu Bei Dan Ci” (Don’t Memorize Words), Baidu Netdisk, and Bilibili. I used the Ebbinghaus forgetting curve for vocabulary memorization and learned vocabulary, grammar, and reading through online courses. My core goal was to prepare for postgraduate exams.

JTC: What are your most frequently used platforms/courses/tutor services, and why did you choose them?

ZJJ: I often used platforms like Qihang Education’s app and courses by teachers like Tang Chi and Liu Xiaoyan, focusing on vocabulary and reading. I planned to study translation and cloze tests later. These were popular among university students and had good teaching effects.

JTC: What is the general situation of your equipment, internet access, and learning space? How do these conditions affect your learning experience?

ZJJ: I used a tablet for studying, but its storage space was slightly insufficient. The device was lightweight and convenient for organizing materials, directly impacting learning efficiency.

Family Support → Learning Outcomes (Direct Effect)

JTC: Looking back at this semester, was there an incident where family support significantly changed your learning results? Please tell the full story: “cause—what your family did—what you did as a result—outcome.”

ZJJ: My family supported me in registering for a postgraduate exam tutoring class, solving problems related to learning paths and resources. This allowed me to focus more on studying, improving efficiency and confidence.

JTC: Emotionally, how has your family’s attitude or companionship motivated you or helped you persist? When did this happen, and how was it reflected in your final grades/homework/oral output?

ZJJ: My family always respected my learning choices and provided encouragement. For example, they supported me in buying CET-4 materials, helping me pass the exam smoothly.

JTC: Materially, did your family provide equipment, internet, a fixed learning space, or course funding? Which had the most direct impact on your learning output? Please give an example.

ZJJ: My family provided equipment (a tablet), funding for learning materials, etc. The tablet had the most direct impact on improving learning outcomes, as it facilitated material management and study scheduling.

JTC: Academically, did your family participate in making plans, checking progress, or practicing oral English with you? Please share a specific experience where “a change in method led to a change in results.”

ZJJ: My family didn’t participate in making specific study plans, only offering suggestions. I mainly formulated and implemented learning strategies on my own.

JTC: In terms of rules, did your family have agreements on time management or phone usage? How were these rules enforced, and what impact did they have on your learning results?

ZJJ: After entering university, my family was more tolerant of my phone usage, setting no strict restrictions and granting me high autonomy.

JTC: If you had not received or received less of the above support, how do you think the results might have been different? Why?

ZJJ: Without financial and equipment support from my family, my learning efficiency and progress would have decreased, and anxiety would have increased.

JTC: Have you observed gaps in learning among classmates due to differences in family support? Can you give a comparative example from your surroundings?

ZJJ: I feel that my classmates generally progress faster than me, but I don’t know about differences in their family support.

Self-Regulation → Learning Outcomes (Direct Effect)

JTC: Please describe a complete cycle of your exam preparation/project learning: how you set goals, arranged time, monitored progress, and reviewed.

ZJJ: I took one month as a learning cycle, studying six days a week and resting one day. Daily arrangements included memorizing professional courses, practicing English vocabulary and real exams, and studying politics courses and exercises. The schedule was tight.

JTC: What are the three most common distractions you encounter while studying (e.g., social media, noise, task accumulation)? What specific strategies have you adopted to cope with them?

ZJJ: Common distractions included phone usage, task accumulation, and lack of sleep. Coping strategies: placing the phone in another room to reduce interference, and gradually completing accumulated tasks through consistent study.

JTC: Has there been a time when good self-regulation led to a significant improvement in results? What strategies did you use, how long did it last, and what changes occurred?

ZJJ: I think my daily learning efficiency is relatively stable; there was no obvious experience where self-regulation improved learning outcomes.

JTC: Also, please share an experience where self-regulation failed: what happened? How did you recover and get back on track later?

ZJJ: After a seven-day holiday, I struggled to resume studying. I forced myself to study every day to gradually regain my rhythm.

Positive Emotions → Learning Outcomes (Direct Effect)

JTC: In online learning, when do you clearly feel interested, engaged, happy, or confident? What situations usually trigger these emotions (e.g., anonymous/semi-anonymous interaction, recorded lectures, autonomous pacing, teacher feedback)?

ZJJ: I felt interested, engaged, and confident when the learning content was familiar or the teacher’s teaching style was lively and humorous. The teacher’s style directly affected my emotional state and learning outcomes.

JTC: Please share an experience where “the emergence of positive emotions led to specific behaviors and changes in learning output.”

ZJJ: For example, when the teacher told interesting or educational jokes during online classes, it caught my attention, helping improve focus and learning efficiency.

JTC: Did the “physical absence” of the online environment make you more willing to participate or speak up? What did you do? How did your subsequent homework/quizzes/oral performance differ?

ZJJ: Online, I was usually unwilling to speak up, lacking the desire to participate.

JTC: Conversely, what situations cause anxiety, boredom, or fatigue? How do you usually adjust and return to an effective learning state?

ZJJ: (No response provided in the original text.)

Indirect Effect of Family Support: Via Self-Regulation/Positive Emotions

JTC: Please give a complete “chain story” for each: (1) family support → change in self-regulation → learning outcome; (2) family support → improvement in positive emotions → learning outcome.

ZJJ: With material and emotional support from my family, I became more proactive in learning, improving efficiency; family support also made me very optimistic, reducing boredom with studying and allowing me to focus without distraction.

JTC: From your experience, which chain is more critical for you? Why?

ZJJ: I think the first is most critical. Without self-discipline, I wouldn’t seek family support to study hard. Self-awareness leads to better learning outcomes—studying for oneself.

JTC: Do these two chains interact (e.g., becoming more disciplined first and then in a better mood, or being in a positive mindset first and thus easier to persist)? Please illustrate with a specific example.

ZJJ: I think they interact. Self-discipline brings engagement in learning, and a positive attitude helps with persistence. Self-regulation is the foundation of learning effectiveness; family support and positive emotions depend on self-motivation. Without the willingness to learn, external support and emotional motivation are ineffective.

Interaction of Multiple Factors and Framework Verification (Member Checking)

JTC: If your experience were summarized as “family support influences self-regulation and positive emotions, which in turn affect online learning outcomes,” how well do you think this framework aligns with your situation?

ZJJ: I think it aligns well. For me, family support indirectly promoted my learning efficiency by enhancing self-regulation and positive emotions.

JTC: In your experience, are there any other critical factors we haven’t mentioned (e.g., peers, teacher feedback, course difficulty, evaluation methods)? How do they enter your learning process?

ZJJ: I think peers and environment are closely related. Positive peers can drive learning motivation when studying together; conversely, they can easily distract.

JTC: If you had to rank them, what are the top three most critical factors for you, and why?

ZJJ: First, self-regulation; second, family support; third, positive emotions. I believe self-regulation is the reason for receiving family support. One must first have self-regulation to seek family support. If one doesn’t want to do something, no amount of support is voluntary.

Potential Impact of Smart Education Technology

JTC: What online English platforms or features have you used (e.g., adaptive learning, personalized recommendations, live tutor sessions, small-group interaction, gamified incentives)? Please explain why you chose them.

ZJJ: I used platforms like Duolingo and “Bu Bei Dan Ci,” valuing their personalized learning paths and adaptability, suitable for students from basic to intermediate levels.

JTC: Choose one of your most frequently used or memorable features. Explain how it changed your goal-setting, progress monitoring, emotional experience, or participation, and ultimately what learning outcomes it brought.

ZJJ: I don’t think it changed my big goal of postgraduate exams. For mid-term goals like vocabulary, the app recorded progress, ultimately greatly helping my learning outcomes.

JTC: Have these technologies had any side effects (e.g., interrupted attention, information overload, anxiety)? How do you balance the pros and cons?

ZJJ: I think learning apps have some side effects, sometimes distracting me to do irrelevant things. But I can manage myself by locking unrelated apps.

Resource Equity and Accessibility (Differences and Opportunities)

JTC: Compared to classmates, what advantages or limitations do you have in terms of equipment, internet, learning space, access to courses/tutors, or family attitudes?

ZJJ: I have no obvious advantages or limitations compared to classmates in these aspects.

JTC: How do these differences manifest as gaps in learning outcomes at critical times (e.g., exam preparation, important assignments, projects)? Please give an example.

ZJJ: I haven’t felt obvious differences in these aspects.

JTC: Have you observed cases where gaps were made up through strategies or support? How?

ZJJ: I have observed cases where classmates made up gaps by taking classes or hiring tutors, with obvious short-term improvement.

Recommendations and Improvements (Action-Oriented)

JTC: Based on your experience, please give one actionable recommendation each for students, parents, schools, or platforms (specify what to do, how to do it, and frequency).ZJJ: Students should strengthen communication with classmates, at least once a day, to obtain information and share experiences. Parents should pay more attention to students’ mental states, communicating regularly to prevent excessive study pressure. Schools should reduce ineffective activities and concentrate resources on encouraging learning behaviors.

JTC: If you were to engage in online learning again next semester, what three things would you change first? Why?

ZJJ: I would first identify suitable teachers, purchase course memberships, and prepare learning materials in advance. Matching teachers improve efficiency; memberships provide resources like courseware; advance preparation aids review and summary.

- Wrap-up and Member Checking

JTC: I will briefly recap the key points you shared today. Please confirm if they are accurate and if anything is missing.

ZJJ: I think your recap is accurate with no omissions.

JTC: After the interview is transcribed, would you be willing to assist in verifying key summaries or supplementing materials if needed?

ZJJ: I am willing to cooperate with subsequent summary verification and material supplementation.

- Interview Time: September 11, 2025
- Interviewer: JTC
- Interviewee: Li Linfeng

Interview Content:

JTC: This interview aims to understand your online English learning experiences and feelings this semester. It is completely anonymous and for academic research only. You may agree to be recorded and may terminate the interview at any time.

JTC: Please use specific stories/situations to illustrate (when it happened, who was involved, what you did, and what the result was).

Warm-up and Learning Profile

JTC: Please summarize your online English learning experiences this semester in 2-3 minutes: where you mainly studied, how you studied, and your core goal.

LLF: This semester, I mainly studied English through apps like Bai Ci Zhan (Hundred Words Chopping), Baidu Netdisk, and Shan Bei Postgraduate Exam. I spent about half an hour a day memorizing words and learned reading comprehension, translation, etc., through online course resources. My core goal was postgraduate exams.

JTC: What are your most frequently used platforms/courses/tutor services, and why did you choose them？

LLF: I often used Shan Bei Postgraduate Exam, Tang Chi’s reading courses, Shi Leipeng’s composition courses, and Liu Xiaoyan’s grammar courses. I planned to study translation and new question types later. I chose them because they aligned with my current learning plan and I believed they would improve my studies.

JTC: What is the general situation of your equipment, internet access, and learning space? How do these conditions affect your learning experience?

LLF: I used my phone and physical books for studying, but the phone screen was a bit small for online classes, making it tiring. Physical books were inconvenient to carry—a hassle—but I felt they helped me concentrate more.

Family Support → Learning Outcomes (Direct Effect)

JTC: Looking back at this semester, was there an incident where family support significantly changed your learning results? Please tell the full story: “cause—what your family did—what you did as a result—outcome.”

LLF: My family supported my decision to take postgraduate exams and provided substantial financial support. They let me buy relevant materials and books, allowing me to pursue exams with confidence.

JTC: Emotionally, how has your family’s attitude or companionship motivated you or helped you persist? When did this happen, and how was it reflected in your final grades/homework/oral output?

LLF: Yes, my family has always been there for me, often video-calling to check in and understanding and respecting my choices. They supported me when I studied for CET-4 and now encourage me not to be discouraged while retaking CET-6.

JTC: Materially, did your family provide equipment, internet, a fixed learning space, or course funding? Which had the most direct impact on your learning output? Please give an example.

LLF: My family provided funding for learning materials and a phone. I think funding had the most direct impact—without it, I couldn’t prepare for studies, and my learning plan would be hard to implement.

JTC: Academically, did your family participate in making plans, checking progress, or practicing oral English with you? Please share a specific experience where “a change in method led to a change in results.”

LLF: My family gave me many suggestions, telling me to learn more about postgraduate exam-related information, but didn’t participate much in plan-making, granting me high autonomy.

JTC: In terms of rules, did your family have agreements on time management or phone usage? How were these rules enforced, and what impact did they have on your learning results?LLF: No agreements on phone usage; it had no impact on my learning.

JTC: If you had not received or received less of the above support, how do you think the results might have been different? Why?

LLF: Without my family’s support, I might have felt anxious and uneasy about postgraduate exams, reducing my interest and efficiency in studying.

JTC: Have you observed gaps in learning among classmates due to differences in family support? Can you give a comparative example from your surroundings?

LLF: Most classmates around me progressed faster in postgraduate exam studies than I did. I started later, and I think classmates with good electronic equipment like tablets were more efficient.

Self-Regulation → Learning Outcomes (Direct Effect)

JTC: Please describe a complete cycle of your exam preparation/project learning: how you set goals, arranged time, monitored progress, and reviewed.

LLF: I generally took one month as a learning cycle, with no fixed rest schedule—if I completed tasks well, I’d take half a day off. Daily arrangements focused on memorizing professional courses, followed by English vocabulary and real exams, and finally politics online courses and practice. The schedule was tight, with high pressure.

JTC: What are the three most common distractions you encounter while studying (e.g., social media, noise, task accumulation)? What specific strategies have you adopted to cope with them?

LLF: Main distractions were phone usage, hot weather making rooftop memorization difficult, and late start leading to task accumulation. Coping strategies: placing the phone aside to control usage, and persisting in studying to reduce accumulated tasks.

JTC: Has there been a time when good self-regulation led to a significant improvement in results? What strategies did you use, how long did it last, and what changes occurred?

LLF: My daily learning was relatively stable—waking up around 7 a.m. and studying until about 10 p.m., lasting two months during summer vacation. Finally, progress accelerated and efficiency improved.

JTC: Also, please share an experience where self-regulation failed: what happened? How did you recover and get back on track later?

LLF: One day, I was in a bad mood due to many troubles and couldn’t focus on studying, resulting in very low efficiency. Later, I relieved stress by chatting with a study partner and listening to music.

Positive Emotions → Learning Outcomes (Direct Effect)

JTC: In online learning, when do you clearly feel interested, engaged, happy, or confident? What situations usually trigger these emotions (e.g., anonymous/semi-anonymous interaction, recorded lectures, autonomous pacing, teacher feedback)?

LLF: When listening to teachers for a long time, I would fully immerse myself, wanting to understand and master knowledge. Curiosity made me confident. I usually became engaged when teachers asked questions.

JTC: Please share an experience where “the emergence of positive emotions led to specific behaviors and changes in learning output.”

LLF: When the teacher asked a question, I would pause to think, then check the teacher’s answer against mine after figuring it out. Enjoying this process improved my understanding and retention of knowledge.

JTC: Did the “physical absence” of the online environment make you more willing to participate or speak up? What did you do? How did your subsequent homework/quizzes/oral performance differ?

LLF: In online environments, I was more willing to participate or speak up. I often interacted with teachers during live classes. Teachers’ responses made me feel involved, and I became more engaged in subsequent learning.

JTC: Conversely, what situations cause anxiety, boredom, or fatigue? How do you usually adjust and return to an effective learning state?

LLF: For example, boring, unengaging classes made me feel bored and less interested. I usually switched to another teacher’s online course.

Indirect Effect of Family Support: Via Self-Regulation/Positive Emotions

JTC: Please give a complete “chain story” for each: (1) family support → change in self-regulation → learning outcome; (2) family support → improvement in positive emotions → learning outcome.

LLF: With family support, I became more attentive to my studies, not wanting to live up to my choices or their expectations. This also increased my confidence and focus; family support kept me optimistic and positive about learning, improving efficiency.

JTC: From your experience, which chain is more critical for you? Why?

LLF: The second is most important. My family’s emotional support provided a strong pillar for my studies. Their encouragement kept me passionate about learning.

JTC: Do these two chains interact (e.g., becoming more disciplined first and then in a better mood, or being in a positive mindset first and thus easier to persist)? Please illustrate with a specific example.

LLF: I think a positive mindset makes persistence easier. During my low points, family and friends encouraged me not to be discouraged, giving me great confidence to keep going.

Interaction of Multiple Factors and Framework Verification (Member Checking)

JTC: If your experience were summarized as “family support influences self-regulation and positive emotions, which in turn affect online learning outcomes,” how well do you think this framework aligns with your situation?

LLF: I think it aligns well. Family support greatly satisfied my emotional needs, motivating me to study harder and improving learning outcomes.

JTC: In your experience, are there any other critical factors we haven’t mentioned (e.g., peers, teacher feedback, course difficulty, evaluation methods)? How do they enter your learning process?

LLF: Peers around me had a big impact. Studying with them allowed mutual supervision and progress.

JTC: If you had to rank them, what are the top three most critical factors for you, and why?

LLF: First, family support; second, positive emotions; third, self-regulation. Family support provided great help, leading to positive emotions and improved self-regulation.

Potential Impact of Smart Education Technology

JTC: What online English platforms or features have you used (e.g., adaptive learning, personalized recommendations, live tutor sessions, small-group interaction, gamified incentives)? Please explain why you chose them.

LLF: I used Shan Bei Postgraduate Exam, Bai Ci Zhan, etc. Shan Bei Postgraduate Exam included vocabulary and phrase memorization, long and difficult sentence learning, and analysis, which helped my studies.

JTC: Choose one of your most frequently used or memorable features. Explain how it changed your goal-setting, progress monitoring, emotional experience, or participation, and ultimately what learning outcomes it brought.

LLF: The most commonly used feature was vocabulary and phrase memorization. It provided a benchmark for setting vocabulary learning goals and better monitoring progress, giving me a sense of daily progress. This improved my participation and efficiency.

JTC: Have these technologies had any side effects (e.g., interrupted attention, information overload, anxiety)? How do you balance the pros and cons?

LLF: Yes, messages sometimes distracted me while using the phone. I turned on Do Not Disturb mode for immersive learning.

Resource Equity and Accessibility (Differences and Opportunities)

JTC: Compared to classmates, what advantages or limitations do you have in terms of equipment, internet, learning space, access to courses/tutors, or family attitudes?

LLF: I think I had slight limitations compared to classmates with tablets, but my open family attitude and their encouragement were great advantages.

JTC: How do these differences manifest as gaps in learning outcomes at critical times (e.g., exam preparation, important assignments, projects)? Please give an example.

LLF: During exam preparation, classmates with tablets may have found storing information and materials more convenient, leading to higher efficiency. My family also greatly improved my learning efficiency.

JTC: Have you observed cases where gaps were made up through strategies or support? How?

LLF: I observed classmates made up gaps by taking classes or seeking guidance from senior students who passed exams, leading to obvious short-term efficiency improvements.

Recommendations and Improvements (Action-Oriented)

JTC: Based on your experience, please give one actionable recommendation each for students, parents, schools, or platforms (specify what to do, how to do it, and frequency).

LLF: Students should spend more time studying, start as soon as possible, and communicate more with classmates to share experiences and information. Parents should pay attention to students’ physical and mental health, communicating regularly to prevent excessive pressure or health issues. Schools should provide a good learning environment and regular psychological counseling for students in need.

JTC: If you were to engage in online learning again next semester, what three things would you change first? Why?

LLF: I would first prepare needed learning materials, find suitable online course teachers, and make a full learning plan with timely revisions. Advance preparation improves efficiency; suitable teachers enhance knowledge mastery; a full plan standardizes learning and tracks progress.

Wrap-up and Member Checking

JTC: I will briefly recap the key points you shared today. Please confirm if they are accurate and if anything is missing.

LLF: I think your recap is accurate with no omissions.

JTC: After the interview is transcribed, would you be willing to assist in verifying key summaries or supplementing materials if needed?

LLF: I am very willing to assist with subsequent work.

Interview Time: September 11, 2025

Interviewer: JTC

Interviewee: Xu Cong

Interview Content:

JTC: This interview aims to understand your online English learning experiences and feelings this semester. It is completely anonymous and for academic research only. You may agree to be recorded and may terminate the interview at any time.

JTC: Please use specific stories/situations to illustrate (when it happened, who was involved, what you did, and what the result was).

Warm-up and Learning Profile

JTC: Please summarize your online English learning experiences this semester in 2-3 minutes: where you mainly studied, how you studied, and your core goal

.XC: This semester, I mainly studied online English in the dorm and at home. Since I’m from Chenzhou, I studied in the dorm when I had no classes and went home on weekends. The main methods were using phone apps to memorize words, watching English learning videos on Bilibili, and reading news on the China Daily app. My core goal was to pass the CET-4 exam.

JTC: What are your most frequently used platforms/courses/tutor services, and why did you choose them?

XC: I most often used Bai Ci Zhan and Shan Bei Dan Ci to memorize words because they had pictures and spaced repetition reminders, making it more interesting than memorizing from books alone. For listening, I used the Daily English Listening app, which had many free materials like CET-4 real exam listening. I didn’t buy many paid courses, mainly watching free CET-4 skill or grammar courses on Bilibili.

JTC: What is the general situation of your equipment, internet access, and learning space? How do these conditions affect your learning experience?

XC: Equipment was my phone and a pair of headphones. Generally, the phone fully met my learning needs, though there was occasional lag when listening to audio. Overall, my learning efficiency was good.

Family Support → Learning Outcomes (Direct Effect)

JTC: Looking back at this semester, was there an incident where family support significantly changed your learning results? Please tell the full story: “cause—what your family did—what you did as a result—outcome.”

XC: Near the CET-4 exam, I was reviewing at home. My mom noticed my anxiety and poor performance on practice questions, so she suggested I stop writing temporarily. She prepared many snacks and gave me emotional encouragement. After resting, I did a real exam and found my mind worked faster, with higher accuracy.

JTC: Emotionally, how has your family’s attitude or companionship motivated you or helped you persist? When did this happen, and how was it reflected in your final grades/homework/oral output?

XC: Although my parents don’t understand English, they never pressured me or asked, “Why haven’t you passed yet?” When I studied, they silently cared for me, occasionally bringing milk to my room. Their care motivated me to study harder.

JTC: Materially, did your family provide equipment, internet, a fixed learning space, or course funding? Which had the most direct impact on your learning output? Please give an example.

XC: My family bought me a phone, installed WiFi, and gave me ample time and space to study, providing various material support. The quiet home environment made me focus on finishing homework without distractions, improving efficiency.

JTC: Academically, did your family participate in making plans, checking progress, or practicing oral English with you? Please share a specific experience where “a change in method led to a change in results.”

XC: My mom reminded me to make plans, though she didn’t understand the content but occasionally checked if I was doing them seriously. No one at home spoke English, so there was no oral practice.

JTC: In terms of rules, did your family have agreements on time management or phone usage? How were these rules enforced, and what impact did they have on your learning results?

XC: At home, my parents asked me to take breaks after studying for a while to avoid burnout. This taught me to temporarily put aside studies to relax, allowing me to learn more effectively.

JTC: If you had not received or received less of the above support, how do you think the results might have been different? Why?

XC: If the internet was always poor or the learning environment noisy, I would have been more easily distracted, with insufficient practice and unaddressed knowledge gaps. Exam results would likely have been worse. Without emotional support, I might have felt more stressed and anxious, making it harder to learn.

JTC: Have you observed gaps in learning among classmates due to differences in family support? Can you give a comparative example from your surroundings?

XC: I haven’t discussed this with other CET-4 candidates and haven’t noticed gaps caused by differences in family support.

Self-Regulation → Learning Outcomes (Direct Effect)

JTC: Please describe a complete cycle of your exam preparation/project learning: how you set goals, arranged time, monitored progress, and reviewed.

XC: One month before the exam, I set a goal of doing 2 sets of real exams per week. Time arrangement: complete full sets on weekends, and analyze part of the wrong questions each weekday. Monitoring: I usually checked off tasks on a calendar. Review: focused on recurring mistakes in listening and unfamiliar words in reading.

JTC: What are the three most common distractions you encounter while studying (e.g., social media, noise, task accumulation)? What specific strategies have you adopted to cope with them?

XC: First, phone notifications—I used the Forest app to lock my phone to avoid distractions. Second, roommate chat—I wore noise-canceling headphones to reduce their voices. Third, task accumulation anxiety—I split large tasks into 15-minute daily subtasks.

JTC: Has there been a time when good self-regulation led to a significant improvement in results? What strategies did you use, how long did it last, and what changes occurred?

XC: Two weeks before the exam, I strictly practiced listening for 1 hour + memorized 30 words daily for 14 days. As a result, my listening score improved significantly compared to the mock exam at the beginning of the month, which made me very happy.

JTC: Also, please share an experience where self-regulation failed: what happened? How did you recover and get back on track later?

XC: One weekend, I planned to study but couldn’t resist scrolling short videos all afternoon. I felt regretful in the evening, so I immediately wrote a detailed plan for the next day and asked my roommate to supervise me. I got back on track the next day.

Positive Emotions → Learning Outcomes (Direct Effect)

JTC: In online learning, when do you clearly feel interested, engaged, happy, or confident? What situations usually trigger these emotions (e.g., anonymous/semi-anonymous interaction, recorded lectures, autonomous pacing, teacher feedback)?

XC: When watching Bilibili skill videos and suddenly understanding a grammar point, or getting all reading questions right, I feel particularly confident and happy.

JTC: Please share an experience where “the emergence of positive emotions led to specific behaviors and changes in learning output.”

XC: Once, I scored high on a speaking app, which made me happy. I took the initiative to practice for another half hour and tried more difficult materials. After that, I was no longer afraid of speaking.

JTC: Did the “physical absence” of the online environment make you more willing to participate or speak up? What did you do? How did your subsequent homework/quizzes/oral performance differ?

XC: Yes. I find it easier to type questions in online discussion forums than raise my hand in class. I asked once, and the teacher gave me a detailed reply. I remembered that knowledge point deeply and was very happy when it appeared on the exam.

JTC: Conversely, what situations cause anxiety, boredom, or fatigue? How do you usually adjust and return to an effective learning state?

XC: I feel anxious when I can’t understand listening passages. I pause, drink water, walk around, tell myself that wrong answers help identify weaknesses, then go back to listen with the transcript.

- Indirect Effect of Family Support: Via Self-Regulation/Positive Emotions

JTC: Please give a complete “chain story” for each: (1) family support → change in self-regulation → learning outcome; (2) family support → improvement in positive emotions → learning outcome.

XC: My mom reminded me to make plans. After listing weekly plans and implementing them self-disciplinedly, my review became more comprehensive; my parents never pressured me and cared for me deeply, making me feel relaxed and encouraged. I could focus better on studying, greatly improving efficiency.

JTC: From your experience, which chain is more critical for you? Why?

XC: I think the first is more critical. For me, specific plans and execution directly lead to learning outcomes more than emotional motivation.

JTC: Do these two chains interact (e.g., becoming more disciplined first and then in a better mood, or being in a positive mindset first and thus easier to persist)? Please illustrate with a specific example.

XC: Yes. For example, I persisted with my plan for 3 days, which gave me a strong sense of accomplishment. This good mood made it easier to stick to the plan on the fourth day, forming a positive cycle.

Interaction of Multiple Factors and Framework Verification (Member Checking)

JTC: If your experience were summarized as “family support influences self-regulation and positive emotions, which in turn affect online learning outcomes,” how well do you think this framework aligns with your situation?

XC: It aligns well. My family’s support really made me more self-disciplined and in a better mindset, allowing me to focus on learning and achieve satisfactory results.

JTC: In your experience, are there any other critical factors we haven’t mentioned (e.g., peers, teacher feedback, course difficulty, evaluation methods)? How do they enter your learning process?

XC: Course difficulty and teacher feedback. CET-4 real exams were quite difficult for me, but skill videos by Bilibili teachers were very helpful, teaching me how to cope.

JTC: If you had to rank them, what are the top three most critical factors for you, and why?

XC: First, self-regulation—it’s the internal factor determining whether I study. Second, family support—it’s the external factor providing a good environment and mindset. Third, learning platforms and resources—they are good tools for learning.

Potential Impact of Smart Education Technology

JTC: What online English platforms or features have you used (e.g., adaptive learning, personalized recommendations, live tutor sessions, small-group interaction, gamified incentives)? Please explain why you chose them.

XC: Picture-based vocabulary memorization on Bai Ci Zhan, spaced repetition on Shan Bei, massive materials on Daily English Listening, and free courses on Bilibili. I chose them because they are free, convenient, and effective.

JTC: Choose one of your most frequently used or memorable features. Explain how it changed your goal-setting, progress monitoring, emotional experience, or participation, and ultimately what learning outcomes it brought.

XC: Picture-based memorization on Bai Ci Zhan. It made vocabulary memorization less boring. I set a daily goal of 100 words, and the app automatically arranged reviews. After persisting for a long time, my vocabulary expanded significantly, and I could understand reading passages better.

JTC: Have these technologies had any side effects (e.g., interrupted attention, information overload, anxiety)? How do you balance the pros and cons?

XC: Yes. App notifications sometimes interrupted me. I turned on Do Not Disturb mode during study time and scheduled specific times to check notifications.

Resource Equity and Accessibility (Differences and Opportunities)

JTC: Compared to classmates, what advantages or limitations do you have in terms of equipment, internet, learning space, access to courses/tutors, or family attitudes?

XC: Advantage: better home internet and space than the dorm. Limitation: no paid courses, fewer oral practice opportunities than some classmates.

JTC: How do these differences manifest as gaps in learning outcomes at critical times (e.g., exam preparation, important assignments, projects)? Please give an example.

XC: I think my speaking is slightly worse than some classmates, and their learning efficiency may be better.

JTC: Have you observed cases where gaps were made up through strategies or support? How?

XC: Yes. A classmate didn’t take paid classes but persisted in finding language partners on free apps and recording her pronunciation to listen repeatedly. She made great progress through strategy and persistence.

Recommendations and Improvements (Action-Oriented)

JTC: Based on your experience, please give one actionable recommendation each for students, parents, schools, or platforms (specify what to do, how to do it, and frequency).

XC: Students should use Do Not Disturb or phone-locking apps while studying to concentrate. Parents should care more about their children’s mental and physical health. Schools should provide more free high-quality mock exams and analysis services, which are important for exam preparation.

JTC: If you were to engage in online learning again next semester, what three things would you change first? Why?

XC: First, make a detailed weekly plan in advance to avoid last-minute cramming. Second, bravely practice speaking using free app features, not fearing mistakes. Third, form an online study group with classmates for mutual supervision and resource sharing.

Wrap-up and Member Checking

JTC: I will briefly recap the key points you shared today. Please confirm if they are accurate and if anything is missing.

XC: I think your summary is accurate with no omissions.

JTC: After the interview is transcribed, would you be willing to assist in verifying key summaries or supplementing materials if needed?

XC: I am willing to assist with verification when needed.

- Interview Time: September 11, 2025
- Interviewer: JTCInterviewee: Li Dingzhuo

Interview Content:

JTC: This interview aims to understand your online English learning experiences and feelings this semester. It is completely anonymous and for academic research only. You may agree to be recorded and may terminate the interview at any time.

JTC: Please use specific stories/situations to illustrate (when it happened, who was involved, what you did, and what the result was).

Warm-up and Learning Profile

JTC: Please summarize your online English learning experiences this semester in 2-3 minutes: where you mainly studied, how you studied, and your core goal.LDZ: I mainly prepared for postgraduate exams and CET-4/CET-6 by memorizing vocabulary through English learning apps, studying online grammar courses, and watching English exam skill videos. My core goal was to improve my English level to cope with exams.

JTC: What are your most frequently used platforms/courses/tutor services, and why did you choose them?

LDZ: I most often used the “Bu Bei Dan Ci” (Don’t Memorize Words) app because it was convenient and offered free vocabulary memorization features. I didn’t use other platforms due to lack of understanding and peer recommendations.

JTC: What is the general situation of your equipment, internet access, and learning space? How do these conditions affect your learning experience?

LDZ: I used a tablet for studying with no additional funding. The tablet had a larger screen and higher learning efficiency, making it the most direct factor affecting my learning output.

- Family Support → Learning Outcomes (Direct Effect)

JTC: Looking back at this semester, was there an incident where family support significantly changed your learning results? Please tell the full story: “cause—what your family did—what you did as a result—outcome.”

LDZ: My family provided financial support, including equipment, internet, learning space, and course funding. This support gave me the confidence to focus on studying, boosting my confidence in learning.

JTC: Emotionally, how has your family’s attitude or companionship motivated you or helped you persist? When did this happen, and how was it reflected in your final grades/homework/oral output?

LDZ: My family always had an open attitude toward my learning ideas and cared about my studies, giving me great motivation to persist. I made significant progress in postgraduate English learning.

JTC: Materially, did your family provide equipment, internet, a fixed learning space, or course funding? Which had the most direct impact on your learning output? Please give an example.

LDZ: My family provided a mobile phone, tablet, and funding for studies. I think equipment had the biggest impact—tablets are more portable and enable intelligent learning.

JTC: Academically, did your family participate in making plans, checking progress, or practicing oral English with you? Please share a specific experience where “a change in method led to a change in results.”

LDZ: My family didn’t participate in making plans; I arranged and studied independently with high autonomy.

JTC: In terms of rules, did your family have agreements on time management or phone usage? How were these rules enforced, and what impact did they have on your learning results?

LDZ: My family didn’t impose any restrictions, which had little impact on my learning.

JTC: If you had not received or received less of the above support, how do you think the results might have been different? Why?

LDZ: I might have spent most of my time on other activities, reducing study time. Thus, efficiency would have been lower with poor results.

JTC: Have you observed gaps in learning among classmates due to differences in family support? Can you give a comparative example from your surroundings?

LDZ: Some classmates had good family conditions with sufficient equipment and resources, while others lacked funds for equipment and sometimes had to work part-time to earn money.

Self-Regulation → Learning Outcomes (Direct Effect)

JTC: Please describe a complete cycle of your exam preparation/project learning: how you set goals, arranged time, monitored progress, and reviewed.

LDZ: I usually made study plans 3-4 months in advance, scheduled daily tasks each night, monitored progress via learning apps, and reviewed regularly.

JTC: What are the three most common distractions you encounter while studying (e.g., social media, noise, task accumulation)? What specific strategies have you adopted to cope with them?

LDZ: Main distractions were social media, heavy task accumulation, and poor study environments. Coping strategies: flexible plan adjustments, controlled electronic device usage, and negotiating with classmates to reduce interference.

JTC: Has there been a time when good self-regulation led to a significant improvement in results? What strategies did you use, how long did it last, and what changes occurred?

LDZ: I once improved my grades through short-term focused review. Before an exam, I strictly demanded myself for a week, leading to better learning outcomes.

JTC: Also, please share an experience where self-regulation failed: what happened? How did you recover and get back on track later?

LDZ: Previously, I couldn’t study due to a bad mood and interrupted learning to play games with friends. Later, I made up for lost progress during rest time to re-enter the learning state.

Positive Emotions → Learning Outcomes (Direct Effect)

JTC: In online learning, when do you clearly feel interested, engaged, happy, or confident? What situations usually trigger these emotions (e.g., anonymous/semi-anonymous interaction, recorded lectures, autonomous pacing, teacher feedback)?

LDZ: I was more likely to feel interested and engaged in non-anonymous interactions and recorded lectures, allowing me to immerse myself in learning.

JTC: Please share an experience where “the emergence of positive emotions led to specific behaviors and changes in learning output.”

LDZ: When in a good mood, I extended study time, felt energized, and achieved better learning outcomes.

JTC: Did the “physical absence” of the online environment make you more willing to participate or speak up? What did you do? How did your subsequent homework/quizzes/oral performance differ?

LDZ: The online environment reduced speaking pressure—even incorrect answers didn’t cause guilt, encouraging me to participate more actively in learning and interactions.

JTC: Conversely, what situations cause anxiety, boredom, or fatigue? How do you usually adjust and return to an effective learning state?

LDZ: I often felt anxious about answering incorrectly. I usually recovered by taking deep breaths and comforting myself.

Indirect Effect of Family Support: Via Self-Regulation/Positive Emotions

JTC: Please give a complete “chain story” for each: (1) family support → change in self-regulation → learning outcome; (2) family support → improvement in positive emotions → learning outcome.

LDZ: Family financial support allowed me to improve my plans and learning content, enhancing learning outcomes; when I failed, family comfort and encouragement lifted my mood, enabling me to re-engage in studies quickly.

JTC: From your experience, which chain is more critical for you? Why?

LDZ: I think the second is most critical. A positive emotional state motivates me to learn actively and improve outcomes.

JTC: Do these two chains interact (e.g., becoming more disciplined first and then in a better mood, or being in a positive mindset first and thus easier to persist)? Please illustrate with a specific example.

LDZ: Yes, they interact. I believe a mindset shift is needed first to engage in learning and persist.

Interaction of Multiple Factors and Framework Verification (Member Checking)

JTC: If your experience were summarized as “family support influences self-regulation and positive emotions, which in turn affect online learning outcomes,” how well do you think this framework aligns with your situation?

LDZ: I think it aligns well. For me, family support influenced my emotions, making me more actively engage in learning.

JTC: In your experience, are there any other critical factors we haven’t mentioned (e.g., peers, teacher feedback, course difficulty, evaluation methods)? How do they enter your learning process?

LDZ: I think teacher feedback increased my learning interest by providing a sense of accomplishment.

JTC: If you had to rank them, what are the top three most critical factors for you, and why?

LDZ: First, teacher feedback—most direct, providing a sense of achievement. Second, peers—studying together boosts efficiency. Third, evaluation methods—reflecting the sense of accomplishment from learning outcomes.

Potential Impact of Smart Education Technology

JTC: What online English platforms or features have you used (e.g., adaptive learning, personalized recommendations, live tutor sessions, small-group interaction, gamified incentives)? Please explain why you chose them.

LDZ: I used apps like “Bu Bei Dan Ci” and Bai Ci Zhan, which enhanced learning motivation and participation through gamification.

JTC: Choose one of your most frequently used or memorable features. Explain how it changed your goal-setting, progress monitoring, emotional experience, or participation, and ultimately what learning outcomes it brought.LDZ: Most memorable was the PK mode on Bai Ci Zhan. It increased my motivation to learn, making me eager to win and thus acquire more English knowledge.

JTC: Have these technologies had any side effects (e.g., interrupted attention, information overload, anxiety)? How do you balance the pros and cons?LDZ: I don’t think there were any side effects.

Resource Equity and Accessibility (Differences and Opportunities)

JTC: Compared to classmates, what advantages or limitations do you have in terms of equipment, internet, learning space, access to courses/tutors, or family attitudes?LDZ: I think I had an advantage in equipment, leading to faster progress than classmates with poorer devices.

JTC: How do these differences manifest as gaps in learning outcomes at critical times (e.g., exam preparation, important assignments, projects)? Please give an example.LDZ: During exam preparation, I could link different devices to improve efficiency and boost grades.

JTC: Have you observed cases where gaps were made up through strategies or support? How?

LDZ: I don’t recall observing such cases.

Recommendations and Improvements (Action-Oriented)

JTC: Based on your experience, please give one actionable recommendation each for students, parents, schools, or platforms (specify what to do, how to do it, and frequency).

LDZ: Students should increase learning interest by rewarding themselves with physical activity after studying. Parents should affirm their children to motivate learning. Schools should organize study-related recreational activities to make learning enjoyable.

JTC: If you were to engage in online learning again next semester, what three things would you change first? Why?

LDZ: I would prepare study equipment, maintain enthusiasm, and participate actively. Good equipment improves efficiency; enthusiasm boosts motivation; active participation enhances learning outcomes.

Wrap-up and Member Checking

JTC: I will briefly recap the key points you shared today. Please confirm if they are accurate and if anything is missing.

LDZ: I think there’s nothing to add.

JTC: After the interview is transcribed, would you be willing to assist in verifying key summaries or supplementing materials if needed?

LDZ: I am willing to assist with verification and supplementation at any time.
